# Design, synthesis and biological evaluation of 2,4-pyrimidinediamine derivatives as ALK and HDACs dual inhibitors for the treatment of ALK addicted cancer

**DOI:** 10.1080/14756366.2022.2121822

**Published:** 2022-09-13

**Authors:** Dafeng Guo, Yu Yu, Binyu Long, Ping Deng, Dongzhi Ran, Lei Han, Jiecheng Zheng, Zongjie Gan

**Affiliations:** a Department of Medicinal Chemistry, College of Pharmacy, Chongqing Medical University, Chongqing, PR China; b Chongqing Research Center for Pharmaceutical Engineering, Chongqing Medical University, Chongqing, PR China

**Keywords:** ALK, HDACs, dual inhibitors, 2,4-pyrimidinediamine, ALK addicted

## Abstract

Simultaneous inhibition of histone deacetylases (HDACs) and anaplastic lymphoma kinase (ALK) could enhance therapeutic activity against ALK addicted cancer cells. Herein, a new series of 2,4-pyrimidinediamine derivatives as ALK and HDACs dual inhibitors were designed, synthesised and evaluated. Compound **12a** which possessed good inhibitory potency against ALK^wt^ and HDAC1, exhibited stronger antiproliferative activity than Ceritinib on ALK positive cancer cell lines though inducing cell apoptosis and cell cycle arrest *in vitro* and *in vivo*. In addition, the mechanism is further verified by the down-regulation of p-ALK protein, and up-regulation of Acetylated histone 3 (Ac-H3) protein in cancer cells. These results suggested that **12a** would be a potential candidate for the ALK addicted cancer treatment.

## Graphical Abstract

**Figure:**
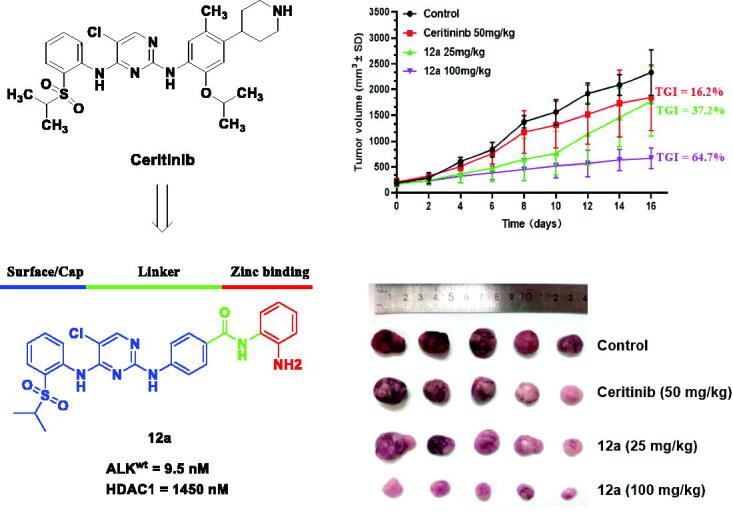

## Introduction

**Figure 1.**
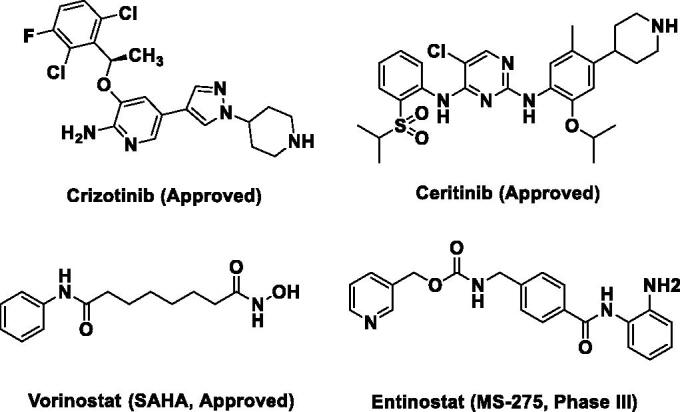
Chemical structures of ALK and HDACs inhibitors.

Anaplastic lymphoma kinase (ALK) is a tyrosine kinase that belongs to the insulin receptor (IR) superfamily^1^^,^^2^. ALK alterations are often involved in the development of several types of cancers including non-small cell lung cancer (NSCLC), anaplastic large cell lymphoma (ALCL) and neuroblastoma^3^^,^^4^. For example, the echinoderm microtubule-associated protein-like 4 (EML4–ALK) fusion, as a common oncogenic gene fusion detected in NSCLC, promotes the dimerisation and phosphorylation of ALK protein, which finally leads to NSCLC occurrence^5^. As a consequence, small molecular ALK inhibitors such as Crizotinib^6^ and Ceritinib^7^ (Figure 1) were approved for the treatment of *ALK*-driven NSCLC via blocking ALK and its downstream signal transduction pathways. However, the effective application of ALK inhibitors is often limited by the drug resistance that emerges following the prolonged treatment in the clinic^8^.

Histone deacetylases (HDACs) are a class of scavenging enzyme that catalysed the removal of acetyl from lysine residues, leading to chromatin condensation and transcriptional suppression in cells^9^^,^^10^. However, aberrant activation of HDACs often contributes to the development and progression of many cancers^11^. Hence, inhibition of HDACs can reverse the genetic aberrations of epigenetic states associated with malignancy^9^^,^^12^. Currently, four small molecule HDACs inhibitors (HDACIs) (Figure 1), Vorinostat (SAHA) ^13^, Belinostat (PXD-101)^14^, Romidepsin (FK228)^15^ and Panobinostat (LBH589)^16^ targeting HDACs to suppress the growth of cancers were approved by FDA and Entinostat (MS-275)^17^ (Figure 1) was being evaluated in the third stages of clinical trials.

As *AXL*-dependent epithelial-to-mesenchymal transition (EMT) regulated by HDACs has been proved to be associated with the emergence of drug resistance to ALK inhibitors, simultaneous inhibition of HDACs could reduce the levels of H3K27ac related to AXL to decrease its gene expression, thus improving the efficacy of ALK inhibitors^18^. Increasing evidences have indicated that ALK inhibitors in combination with HDACIs could synergistically induce the anti-proliferative effects on ALK inhibitor resistant cells or xenografts and are more efficient in ALK positive NSCLC patients^19–22^. In addition to the combinational therapy methods, we conceived that ALK and HDACs dual inhibitors that can concurrently inhibit both targets would be an alternative and attractive therapeutic strategy for ALK addicted cancer.

**Figure 2.**
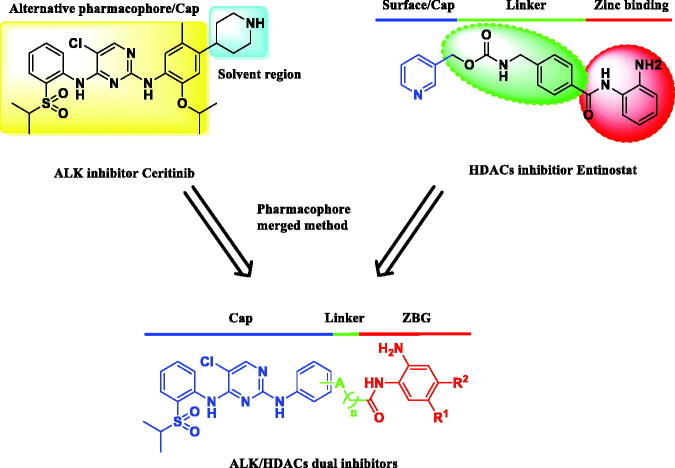
Design strategy for ALK/HDACs dual inhibitors.

In our previous work, we have discovered a series of ALK and HDACs novel dual inhibitors via fused pharmacophore approach^23^. Among them, the optimal compound **10f** featuring a flexible side-chain exhibited good potency on ALK-positive cancer cell lines. However, compound **10f** displayed poor antitumor activities *in vivo*. It was speculated that the hydroxamic acid group might account for the low permeability or aqueous solubility of compound **10f**. Therefore, in this work, a more effective ALK/HDACs dual inhibitor compound **12a**, referring to the structure of Ceritinib and Entinostat, was identified and evaluated (Figure 2). To our delight, **12a** showed a remarkable antitumor efficacy against different cancer cell lines *in vitro* and *in vivo*. These results indicated that **12a** would be a promising anti-NSCLC candidate which deserves further research.

## Results and discussion

### Chemistry

**Scheme 1.**
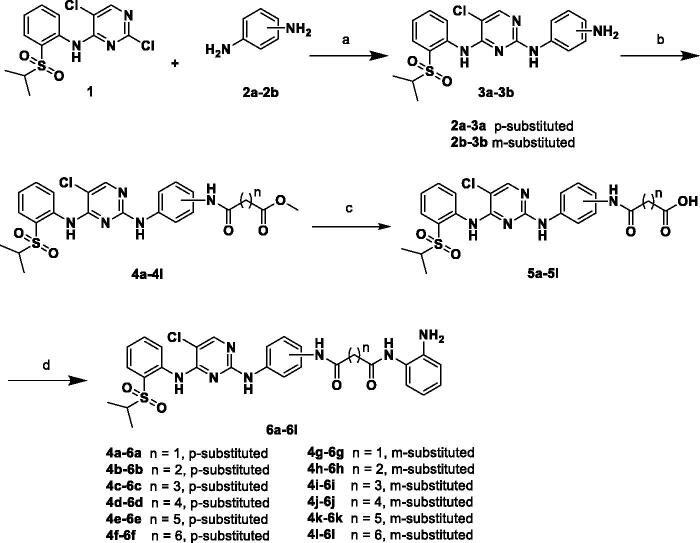
Reagents and conditions: (a) HCl, *i*-PrOH, 90 °C, 6 h, 52–63%; (b) Corresponding methyl alkanoates, HOBT, EDCI, DIPEA, DMF, r.t., 12 h, 24–68%; (c) NaOH, MeOH/H_2_O, 70 °C, 6 h, 79–93%; and (d) HOBT, EDCI, DIPEA, DMF, r.t., 5 h, 25–89%.

**Scheme 2.**
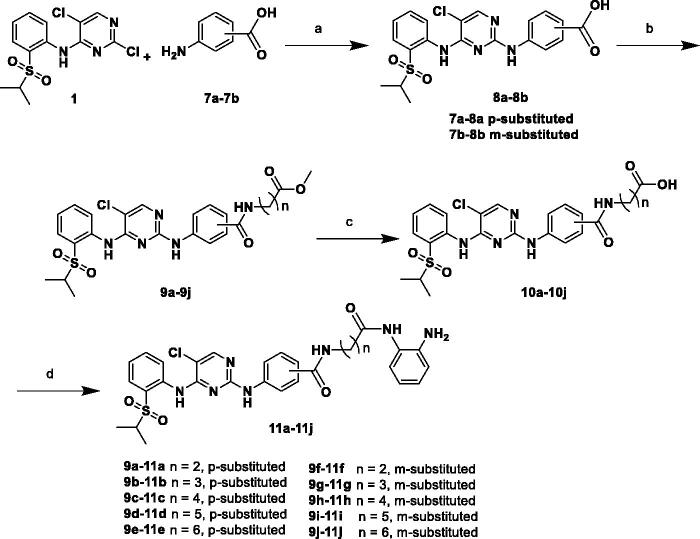
Reagents and conditions: (a) HCl, EtOH, 90 °C, 6 h, 68–88%; (b) Corresponding methyl aminoalkanoates, HOBT, EDCI, DIPEA, DMF, r.t., 12 h, 47–76%; (c) NaOH, MeOH/H_2_O, 70 °C, 6 h, 70–89%; and (d) HOBT, EDCI, DIPEA, DMF, r.t., 5 h, 36–90%.

The desired compounds **6a–l** and **11a–j** were prepared according to the synthetic route depicted in Schemes 1 and 2. 2,5-dichloro-N-(2-(isopropylsulfonyl)phenyl)pyrimidin-4-amine (**1**) was purchased and used as the starting material. With regard to procedures in Scheme 1, m/p-phenylenediamines (**2a–b**) were firstly reacted with compound **1** to obtain intermediates **3a–b**, respectively. Next, methyl alkanoate derivatives were activated by 1-ethyl-3–(3-dimethyllaminopropyl) carbodiimide hydrochloride (EDCI)/1-hydroxybenzotriazole (HOBT), and subsequently condensed with intermediates **3a–b** to produce the key ester intermediates **4a–l** in the presence of N, N-Diisopropylethylamine (DIPEA). Then **4a–l** were hydrolysed by NaOH to afford carboxylic acid intermediates **5a–l**, which were finally condensed with *o*-phenylenediamine to give the target products **6a–l** under the same condensed reaction condition. In Scheme 2, compounds **11a–j** were synthesised by following similar procedures from compound **1** and p/m-aminobenzoic acid (**7a–b**).

**Scheme 3.**
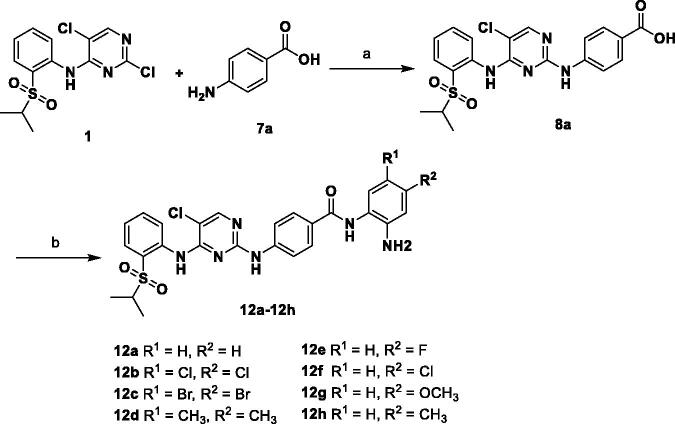
Reagents and conditions: (a) HCl, EtOH, 90 °C, 6 h, 88%; (b) HOBT, EDCI, DIPEA, DMF, r.t., 5 h, 25–44%.

Another series of desired compounds **12a–h** were prepared as described in Scheme 3. Condensation of previously obtained 4-((5-chloro-4-((2-(isopropylsulfonyl)phenyl)amino)pyrimidin-2-yl)amino)benzoic acid (**8a**) with *o*-phenylenediamines yielded the corresponding final compounds **12a–h** in moderate yields.

### Biological evaluation

#### Anti-proliferative activities of target compounds against cancer cell lines

The *in vitro* antiproliferative effects of the target compounds on A549 (lung cancer cells), MDA-MB-231 (breast carcinoma cells), HepG2 (hepatocellular carcinoma cells), and SK-N-BE(2) (neuroblastoma cells) were detected via CCK-8 assay for 72 h, Ceritinib and Entinostat were selected as two positive controls. As shown in Table 1, most of the synthetic compounds displayed stronger inhibitory activity than the positive controls on selected cancer cell line, especially compound **12a**, which inhibited the growth of cells with IC_50_ values ranging from 0.0003 to 0.01 μM, demonstrating that the HDACs inhibitory effects of these dual inhibitors may contribute to their antiproliferative capacities. Notably, SK-N-BE(2) cell line harbouring ALK^wt^ mutation seems to be the most sensitive cancer cell line than others, indicating that our compounds may be more effective on ALK positive cancer cell lines. To further investigate the effect of linker on the antitumor activity, compounds **6a–f** with different linker length (*n* = 2–6) were synthesised. However, it was found that increasing the linker length may be unfavourable for enhancing the anti-proliferative activity. Moreover, introducing electron-withdrawing or electron-donating groups such as F, Cl, CH_3_ groups on the phenyl ring (**12b–h**) also lead to decreased inhibition activity. Interestingly, the meta-substituted compounds (**6g–l**) showed good antitumor activity as well as their para-substituted analog (**6a–f**), indicated that the substituted position may had little effect on inhibition capacities. Finally, in comparison with **6a–l**, compounds **11a–j** were made as reversed amide and they exhibited parallel potency compared to compound **6a–l**.

**Table 1.** Antiproliferative activity of compounds against cancer cell lines.

Compd.	Cell lines/IC_50_ (μM)^a^			
	A549	MDA-MB-231	HepG2	SK-N-BE (2)
**6a**	0.11 ± 0.01	ND^b^	0.02 ± 0.01	0.02 ± 0.001
**6b**	0.82 ± 0.11	0.23 ± 0.04	0.16 ± 0.01	0.02 ± 0.001
**6c**	1.38 ± 0.31	0.49 ± 0.01	0.26 ± 0.01	0.01 ± 0.001
**6d**	1.61 ± 0.46	0.40 ± 0.15	0.35 ± 0.02	0.01 ± 0.003
**6e**	1.09 ± 0.13	0.28 ± 0.07	0.22 ± 0.02	0.01 ± 0.001
**6f**	ND^b^	0.22 ± 0.08	0.20 ± 0.03	ND^b^
**6g**	0.31 ± 0.04	0.16 ± 0.05	0.36 ± 0.02	0.02 ± 0.001
**6h**	0.34 ± 0.05	0.14 ± 0.01	0.19 ± 0.06	0.01 ± 0.002
**6i**	0.50 ± 0.03	0.05 ± 0.001	0.30 ± 0.01	ND^b^
**6j**	0.46 ± 0.10	0.21 ± 0.05	0.34 ± 0.05	0.01 ± 0.001
**6k**	0.67 ± 0.18	0.05 ± 0.01	0.40 ± 0.28	ND^b^
**6l**	0.79 ± 0.12	0.27 ± 0.02	0.54 ± 0.27	0.01 ± 0.001
**11a**	0.47 ± 0.04	0.29 ± 0.001	0.64 ± 0.21	0.02 ± 0.004
**11b**	0.29 ± 0.05	0.25 ± 0.009	0.63 ± 0.15	0.01 ± 0.003
**11c**	0.30 ± 0.03	0.33 ± 0.07	0.72 ± 0.05	0.01 ± 0.001
**11d**	0.28 ± 0.04	0.05 ± 0.03	0.51 ± 0.02	ND^b^
**11e**	0.33 ± 0.04	0.16 ± 0.08	0.42 ± 0.01	0.01 ± 0.002
**11f**	0.38 ± 0.04	0.14 ± 0.04	0.61 ± 0.11	0.02 ± 0.004
**11g**	0.59 ± 0.08	0.07 ± 0.02	0.64 ± 0.24	0.02 ± 0.003
**11h**	0.83 ± 0.03	0.13 ± 0.08	0.85 ± 0.01	0.01 ± 0.002
**11i**	0.75 ± 0.08	0.08 ± 0.07	1.18 ± 0.37	0.01 ± 0.001
**11j**	1.09 ± 0.01	0.13 ± 0.005	2.22 ± 0.72	0.01 ± 0.001
**12a**	0.02 ± 0.001	0.01 ± 0.008	0.03 ± 0.009	0.003 ± 0.002
**12b**	0.21 ± 0.02	0.01 ± 0.005	0.11 ± 0.006	0.02 ± 0.001
**12c**	0.06 ± 0.01	0.03 ± 0.01	ND^b^	0.02 ± 0.002
**12d**	0.35 ± 0.05	0.01 ± 0.001	0.45 ± 0.06	0.03 ± 0.004
**12e**	0.22 ± 0.02	0.05 ± 0.01	0.19 ± 0.05	0.03 ± 0.001
**12f**	0.33 ± 0.02	0.02 ± 0.01	0.20 ± 0.05	0.03 ± 0.006
**12g**	0.38 ± 0.05	0.09 ± 0.01	0.21 ± 0.05	0.02 ± 0.003
**12h**	0.39 ± 0.10	0.13 ± 0.05	0.31 ± 0.06	0.02 ± 0.002
Ceritinib	2.36 ± 0.19	1.09 ± 0.67	0.94 ± 0.30	0.04 ± 0.002
Entinostat	3.20 ± 0.10	2.51 ± 0.27	2.54 ± 0.37	0.43 ± 0.08

^a^The reported data are the mean values from three independent experiments.

^b^Not determined.

Considering the excellent antitumor efficacy, compound **12a** was further selected to evaluate its potency on ALK-dependent H2228 lung cancer cells, and **12a** showed an IC_50_ value of 11 nM against the proliferation of H2228, which was almost 10-fold potent than that of Ceritinib and Entinostat (Table 2). In addition, the binding affinity of compound **12a** to ALK and HDAC1 were investigated in kinase assay. As depicted in Table 2, although less efficient than Ceritinib and Entinostat, compound **12a** showed good inhibitory potency against ALK^wt^ and HDAC1, with IC_50_ values of 9.5 and 1450 nM, respectively (Table 2), indicating that compound **12a** was a dual ALK/HDACs inhibitor.

**Table 2.** *In vitro* inhibitory activity of compound **12a** against cancer cell lines and enzymes.

Compd.	IC_50_ (nM)^a^		Cell lines/IC_50_ (μM)^b^			
	HDAC1	ALK	A549	HepG2	SK-N-BE(2) (ALK^wt^)	H2228 (EML4-ALK)
**12a**	1450	9.5	0.002 ± 0.001	0.01 ± 0.001	0.003 ± 0.002	0.11 ± 0.09
Ceritinib	ND*^c^*	<1.0^[23]^	2.36 ± 0.19	1.06 ± 0.67	0.04 ± 0.01	4.32 ± 0.89
Entinostat	211	ND*^c^*	3.20 ± 0.10	2.51 ± 0.27	0.42 ± 0.08	8.83 ± 0.91

^a^The IC_50_ values are the mean values of two independent experiments.

^b^The IC_50_ values are the mean ± *SD* values of three independent experiments.

^c^Not determined.

#### Compound 12a represses cell invasion and migration in vitro

**Figure 3.**
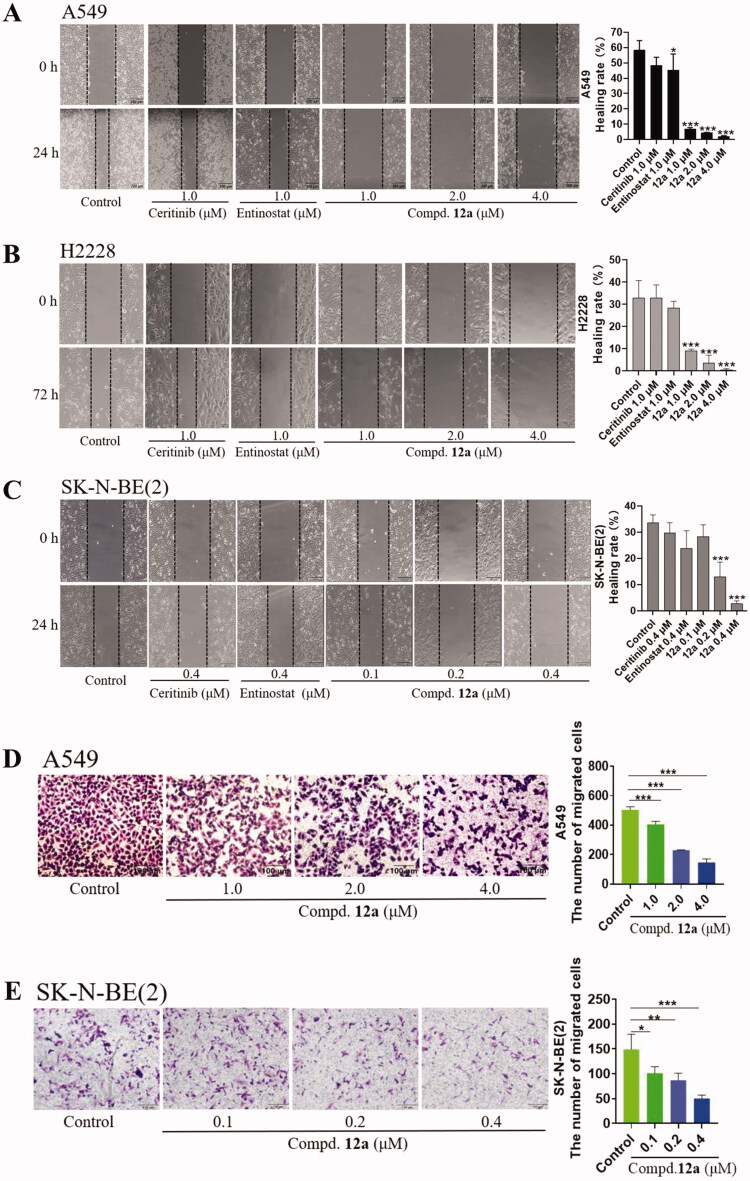
Cell scratch and Transwell assays. Effect of compound **12a** (1.0, 2.0 and 4.0 μM or 0.1, 0.2 and 0.4 μM), Ceritinib (1.0 μM) and Entinostat (1.0 μM) on A549 (A), H2228 (B) and SK-N-BE(2) (C) on cell migration ability. The distance was measured as the mean ± SD (*n* = 3). (D, E) Transwell assay results in SK-N-BE(2) and A549 cell lines treated with Ceritinib (1.0 μM) and compound **12a** (1.0, 2.0 and 4.0 μM or 0.1, 0.2, and 0.4 μM) for 24 h. The number of cells was calculated as the mean ± SD (*n* = 3). Scale bar = 100 μm, *p < 0.05, **p < 0.01 and ***p < 0.001, compared with the control.

Next, we detected the effect of compound **12a** on the migration and invasion ability of A549, H2228 and SK-N-BE(2) cells by cell scratch assay and Transwell method. As shown in Figure 3(A–C), the results showed that the migration capability of all cancer cells was significantly suppressed by **12a** after 24 h treatment, compared with the Ceritinib group and Entinostat group. In Transwell assay, **12a** also reduced the metastasis ability in A549 and SK-N-BE(2) cells at 4.0 μM or 0.4 μM concentration (Figure 3D, E). These results clearly displayed that compound **12a** can prevent the cell migration and invasion in a dose-dependent manner.

#### Compound 12a arrests cell cycle and induces cell apoptosis in vitro

**Figure 4.**
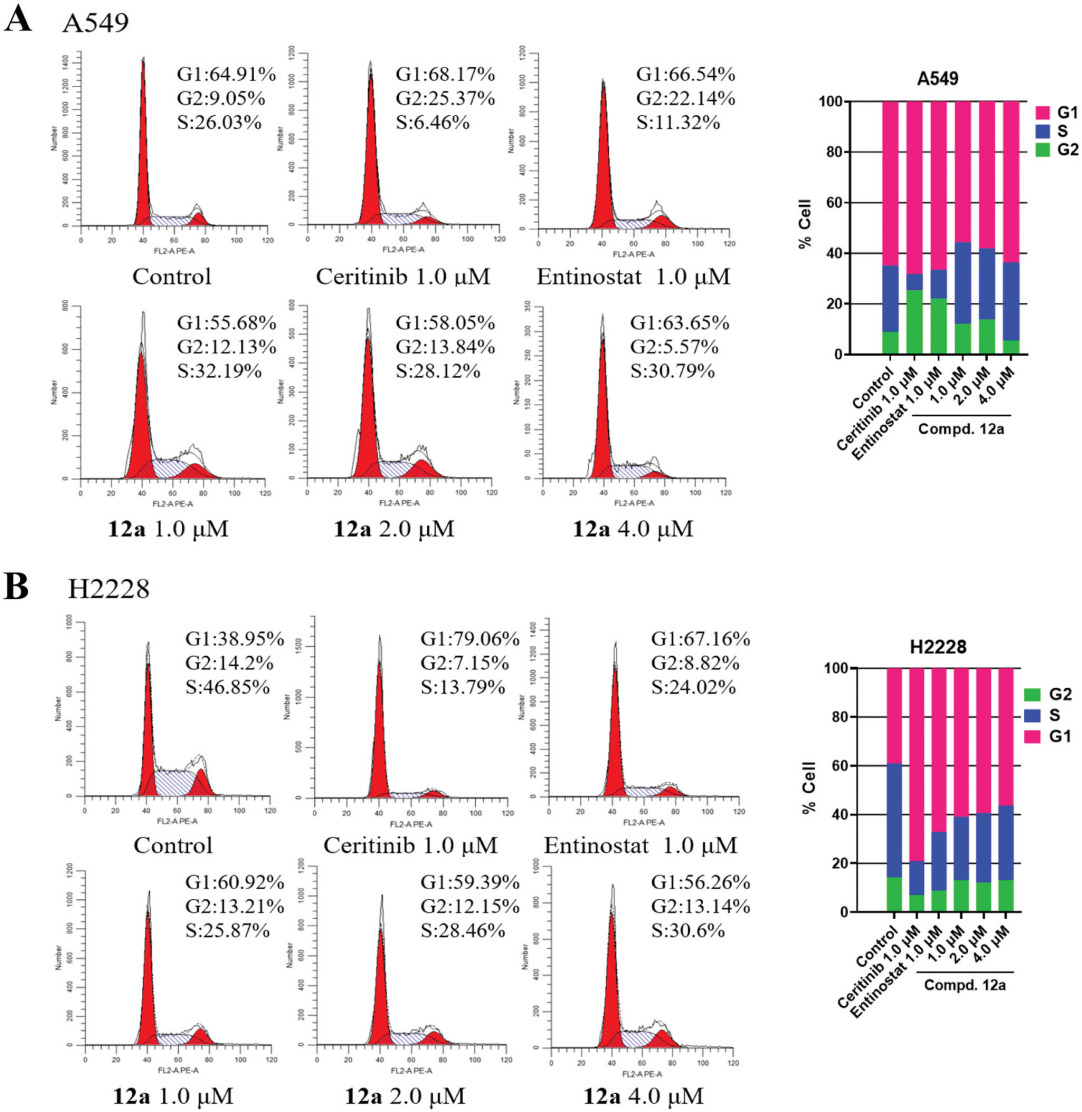
Effect of Compound **12a** on cell cycle progression. A549 and H2228 cells were treated with, Ceritinib (1.0 μM), Entinostat (1.0 μM), compound **12a** (1.0, 2.0 and 4.0 μM) and DMSO (0.8 μL) for 24 h, then stained with PI, followed by analysed via flow cytometry. The bar graph shows the percentages of cells in the G1, G2, S phases. (A) After treatment with compound **12a**, the cell cycle of A549 cells was slightly blocked at S phase, compared with the positive groups or control. (B) The cell cycle was arrested at G1 phase after being treated with compound **12a**, vs. control in H2228 cells.

Since we had demonstrated that compound **12a** is effective to inhibit proliferation of cells *in vitro*, the effect of compound **12a** on the cell cycle was then investigated via flow cytometry to further explore the mechanism. The results of flow cytometry were illustrated in Figure 4(A, B). After A549 cells were treated with **12a** at different concentrations (1.0, 2.0, 4.0 μM) for 24 h, it can be observed that **12a** could slightly arrest cell cycle at S phase. When H2228 cells were treated in the same manner, the percentage of cell population in G1 phase was increased from 38.95% (control) to 60.92% (1.0 μM), indicating that **12a** could arrest the cell cycle at G1 phase in H2228 cells.

**Figure 5.**
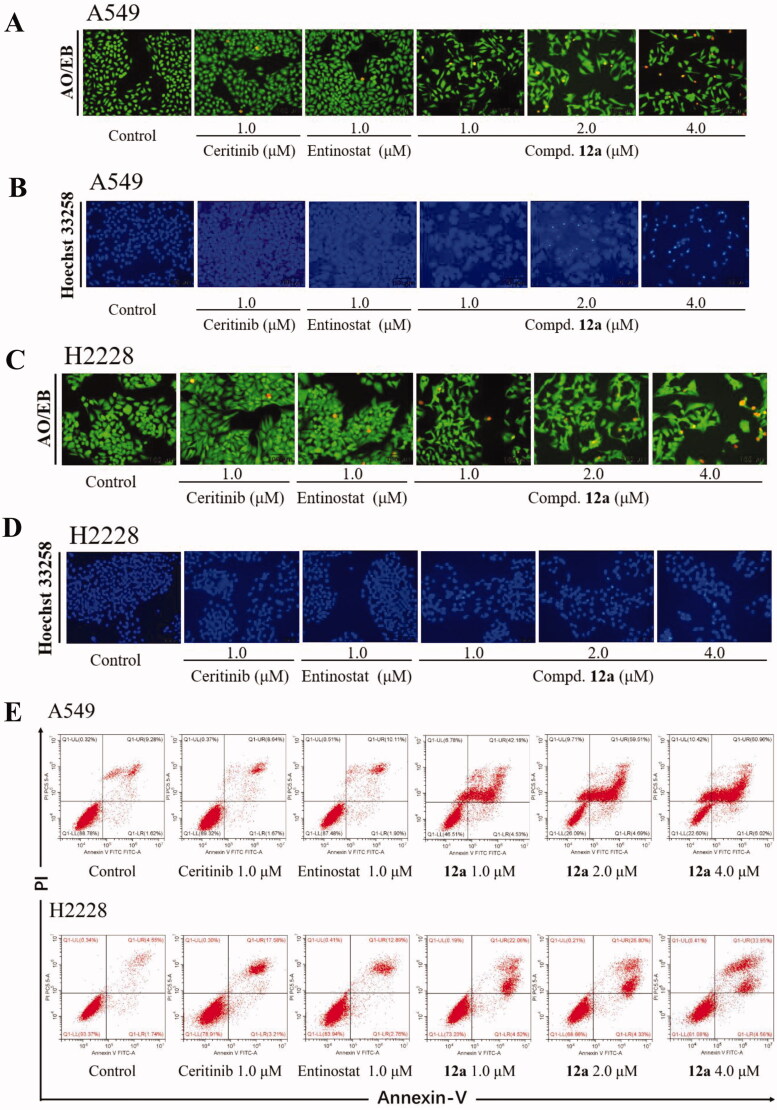
Compound **12a** induced apoptosis in A549 and H2228 cell lines. A549 and H2228 cells were treated with Ceritinib (1.0 μM), Entinostat (1.0 μM), compound **12a** (1.0, 2.0 and 4.0 μM) for 24 h, followed by stained with AO/EB (A and C) or Hoechst 33258 (B and D), and photographed. Scale bar = 100 μm. (E and F) Flow cytometry analysis results. A549 and H2228 cells (E) cells were treated with compound **12a** or positive drugs for 48 h, then stained with Annexin V-FITC/PI.

Furthermore, AO/EB and Hoechst 33258 staining assays were utilised to evaluate whether compound **12a** could induce apoptosis of cells. The AO/EB staining results were illustrated in Figure 5(A, C), it can be seen that the cells in the control group showed well-distributed green fluorescence. On the contrary, after 24 h treatment of compound **12a** at 4.0 μM concentration, the number of orange fluorescent cells increased and the cell morphology gradually changed in A549 and H2228 cells, indicating that the number of late apoptotic cells increased. Similarly, the results of Hoechst 33258 staining further verified that compound **12a** could induce cell death. As illustrated in Figure 5(B, D), the uneven blue fluorescence was emerged and enhanced in a dose-dependent manner in compound treatment groups compared with the control groups. Flow cytometry was further conducted to investigate whether the anti-proliferation effect of **12a** was associated with cellular apoptosis in A549 and H2228 cells. As shown in Figure 5(E), when treated with compound **12a**, the percentage of apoptotic cells increased in a dose-dependent manner, from 10.9% (control) to 46.7% (1.0 μM), 64.2% (2.0 μM), and 66.98% (4.0 μM). Likewise, the apoptosis rate of cells treated with compound **12a** also increased significantly in H2228 cells, much greater than that of control (Figure 5E). From these results, we conclude that **12a** was able to cause apoptotic effects at 1ow concentration in cancer cell lines.

#### Compound 12a blocks ALK and HDAC signalling pathways

**Figure 6.**
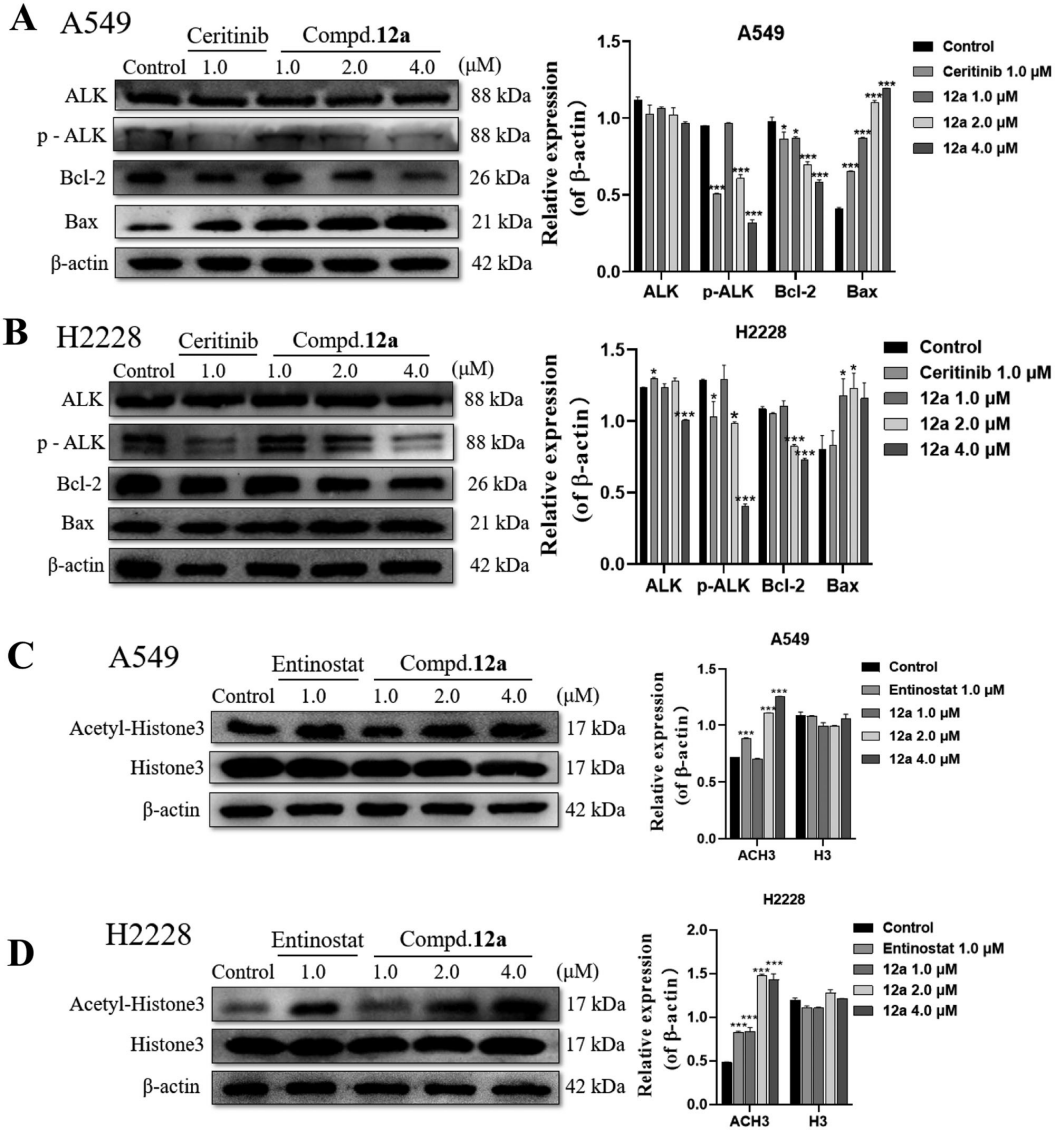
Effect of compound **12a** on the protein expression of ALK- and HDAC-mediated signalling pathways in A549 and H2228 cell lines. (A, B) Western blot analysis of apoptosis-associated and ALK protein expression after treated with compound **12a** in A549 (A) and H2228 (B) cell lines. (C, D) Western blot analysis of Ac-H3 and H3 protein expression after treated with compound **12a** in A549 (C) and H2228 (D) cell lines. All data were expressed as the mean ± SD of three independent experiments. *p < 0.05, ***p < 0.001 compared with untreated samples.

Furthermore, the changes of protein expression related to apoptosis were also investigated by western blot assays in A549 and H2228 cells, the results were illustrated in Figure 6(A, B). Not surprisingly, compound **12a** could dose-dependently up-regulated the expression of pro-apoptotic protein Bax accompanied with a decreased expression of anti-apoptotic protein Bcl-2, which were correlated with previous outcomes.

Meanwhile, to clarify the mechanism, the relative proteins involved in the ALK- or HDAC-mediated signalling pathway were also tested. It was found that the intracellular levels of p-ALK was significantly suppressed in **12a** treatment groups (4.0 μM), compared to that of control group, while the expression of ALK was not changed. On the other hand, after exposure to compound **12a** for 24 h, the expression levels of Acetylated histone 3 (Ac-H3) protein increased in a dose-dependent manner in drug treatment group (Figure 6C, D). Taken together, these results displayed that compound **12a** could block the ALK- and HDAC- mediated signalling pathways simultaneously as a dual inhibitor.

#### Compound 12a inhibits the growth of SK-N-BE(2) cells in vivo

**Figure 7.**
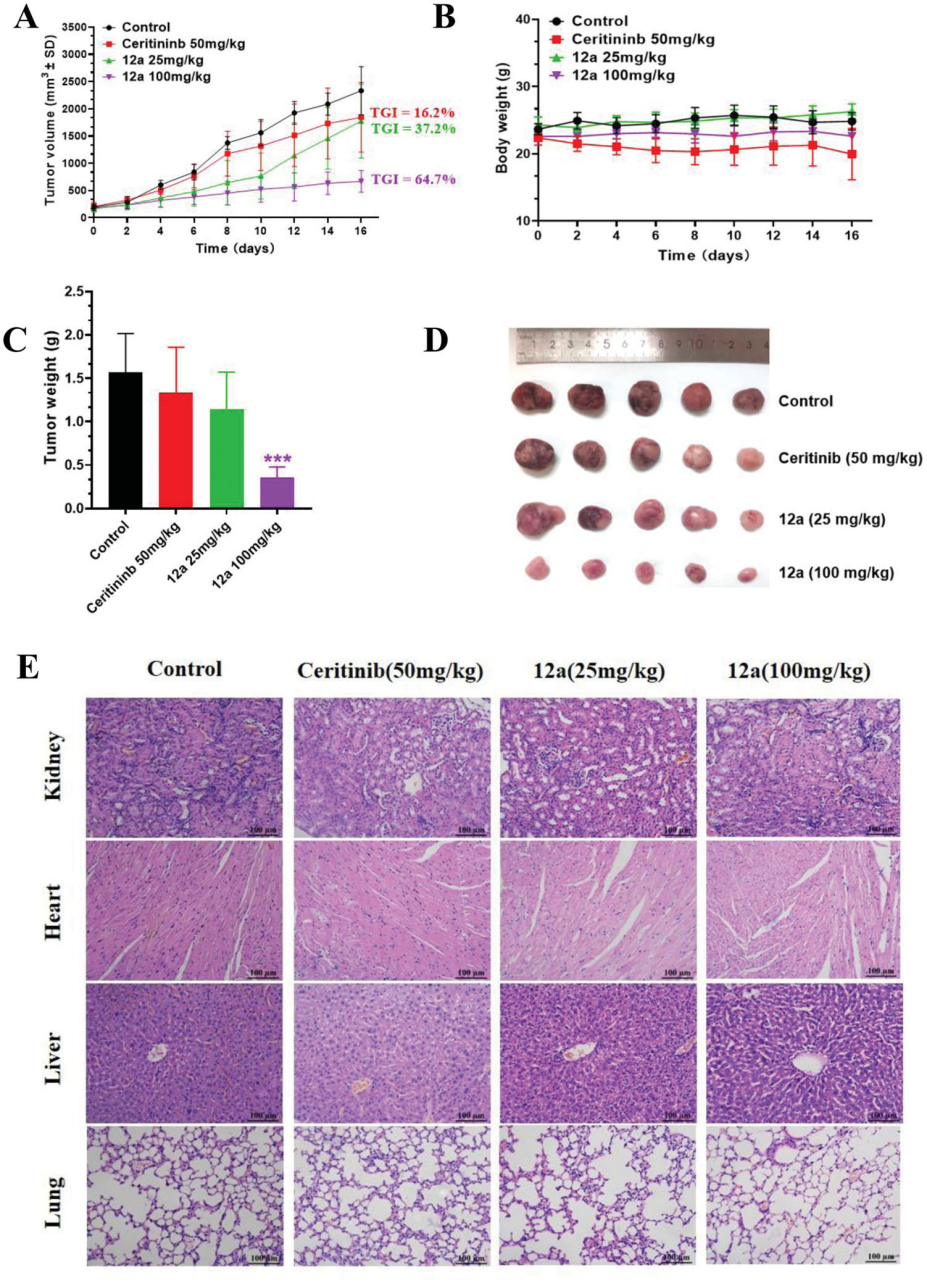
Effect of compound **12a** on the growth of SK-N-BE(2) xenografts *in vivo*. Nude mice were injected with SK-N-BE(2) cells, followed by treatment with compound **12a** (25 mg/kg and 100 mg/kg) and Ceritinib (50 mg/kg) for 16 days. (A) Tumour volume curves. (B) Bodyweight of mice. (C) Tumour weight. (D) Images of tumour xenografts excised from mice model. ***p < 0.005, compared with the control. (E) H&E staining of the organs from the model, control, low-dose and high-dose experimental groups.

On the basis of the favourable *in vitro* anticancer activity, compound **12a** was further selected to evaluate its preliminary antitumor efficacy in the SK-N-BE(2) xenografts *in vivo*^24^. When tumours had reached an average volume of 100 mm^3^, mice were random divided into four groups and intraperitoneally administered with saline, 25 or 100 mg/kg compound **12a** and 50 mg/kg Ceritinib every two days for 16 consecutive days, respectively. The results in Figure 7(A, C) showed that compound **12a** could significantly suppress the tumour growth. Compared with 50 mg/kg Ceritinib, compound **12a** treatment group at a dose of 25 or 100 mg/kg results in 37.2% and 64.7% TGI (tumour growth inhibition), respectively. In addition, no obvious weight loss was observed in all compound treatment groups (Figure 7B). Moreover, the H&E staining results showed no obvious pathological changes were observed in lung, heart and kidney, but vacuolisation in liver were observed in Ceritinib group and high-dose group, indicating that high dose of **12a** may have toxic effects on liver (Figure 7E).

### Molecular docking studies

**Figure 8.**
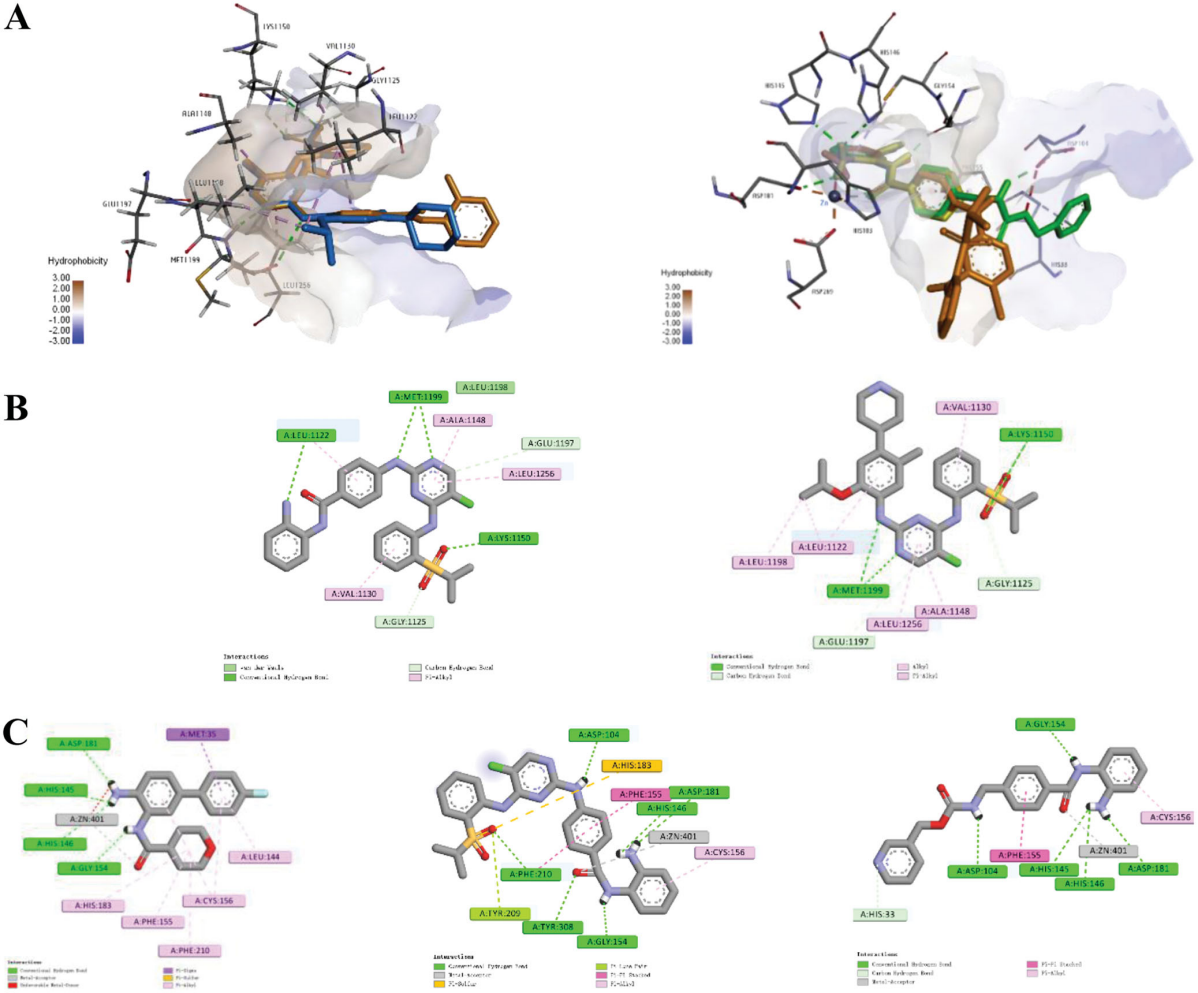
(A) Overlap modelling of **12a** (orange) and Ceritinib (blue) in ALK (PDB: 4MKC) (left); Overlap modelling of **12a** (orange), Entinostat (green) and original ligand (yellow) in HDAC2 (PDB: 5IWG) (right). (B) 2D diagram of the interactions of **12a** (left) and Ceritnib (right) with ALK. (C) 2D diagram of the interactions of original ligand (left), **12a** (middle) and Entinostat (right) with HDAC2.

Docking studies of compound **12a** with ALK and HDAC2 were performed (Figure 8A–C). Similar to Ceritinib, the pyrimidine nitrogen atom in compound **12a** establish strong hydrogen bonds at the hinge area with Met1199. In addition, the phenylamine group was found to be projected towards solvent region which didn’t form key interactions with ALK (Figure 8A, B). On the other hand, from the overlap model of **12a**, the original ligand and Entinostat in HDAC2, we could see that the NH_2_ group and carbonyl group in compound **12a** can coordinate with Zn^2+^ to form a stable six-ring in ZBG pocket, as the original ligand and Entinostat did, which might explain the good inhibitory activity of **12a** against HDAC2 (Figure 8C). Interestingly, it can be observed that the GAP group in compound **12a** and Entinostat adopts a totally opposite orientation, respectively, which might barely affect the HDAC2 inhibitory potency.

## Conclusions

In summary, a novel and potent dual ALK and HDACs inhibitor **12a** was discovered. Compound **12a** exhibited good inhibitory activities against ALK^wt^ or HDAC1 enzymes and synergistically inhibited proliferation of ALK-driven cancer cells via inducing cell apoptosis and cell cycle arrest. Western blot assays were further conducted and confirmed that compound **12a** can concurrently inhibits ALK and HDACs signal pathways as an ALK/HDACs dual inhibitor. More importantly, compound **12a** possessed desirable *in vivo* antitumor potency in a SK-N-BE(2) xenograft model. These results suggested compound **12a** was expected to be a good candidate for the ALK-positive cancer treatment.

## Experimental section

### Chemistry

All materials used in this study were obtained from Tansoole, Macklin and Adamas without further purification after purchasing. The conventional ^1^H NMR and ^13 ^C HNMR spectra were measured in CDCl_3_/DMSO-d_6_ by a Bruker spectrometer with tetramethylsilane (TMS) as internal standard. Coupling constants (*J*) and chemical shifts (δ) are noted in Hz and ppm separately. The melting point of compounds was detected with microscopic melting point apparatus. High-resolution mass spectra (HRMS) were recorded with Aglient 6470 Triple Quad LC-MS apparatus. Column chromatography was conducted on silica gel (200–300 mesh).

#### General procedure for the synthesis of intermediates 3a–b

To a solution of *p*-phenylenediamine (1.25 g, 11.6 mmol, **2a**) in 50 mL isopropanol, was added 2,5-dichloro-N-(2-(isopropylsulfonyl)phenyl)pyrimidin-4-amine (3.48 g, 10.0 mmol, **1**) and 37% HCl solution (0.8 mL). The mixture was heated and stirred at 90 °C for 11 h. After reaction completion, the solvent was evaporated *in vacuo* and extracted with 100 mL EtOAc twice, then the organic layer was sequentially washed with saturated sodium chloride aqueous solution twice, dried over anhydrous Na_2_SO_4_ and filtered to give desired compound **3a** which can be directly used without further purification. **3b** was synthesised in the manner of **3a**.

#### General procedure for the synthesis of intermediates 4a–l

##### Methyl 3-((4-((5-chloro-4-((2-(isopropylsulfonyl)phenyl)amino)pyrimidin-2-yl)amino)phenyl)amino)-3-oxopropanoate (4a)

To a solution of monomethyl malonate hydrochloride (0.58 g, 4.96 mmol) in dry DMF, HOBT (0.65 g, 4.80 mmol), EDCI (0.91 g, 4.77 mmol) and DIPEA (1.24 g, 9.60 mmol) were added. The resulting solution was stirred at room temperature for 0.5 h. Then, **3a** (1.07 g, 2.37 mmol) was added to the solution and stirred for another 12 h. Then the reaction mixture was extracted by ethyl acetate and washed with brine. The organic layer was dried overnight with anhydrous Na_2_SO_4_, filtered and concentrated under reduced pressure. The obtained powder was purified by column chromatography eluting on silica gel with DCM/methanol (30:1) to obtain 0.43 g of **4a** as a white crystal. Yield: 35%. M.p. 175.2–177.3 °C. ^1^H NMR (600 MHz, DMSO-d_6_) δ 10.09 (s, 1H), 9.51 (s, 1H), 9.46 (s, 1H), 8.59 (s, 1H), 8.27 (s, 1H), 7.85 (dd, *J* = 8.0, 1.6 Hz, 1H), 7.76–7.70 (m, 1H), 7.54 (d, *J* = 8.5 Hz, 2H), 7.44 (d, *J* = 9.0 Hz, 2H), 7.41–7.38 (m, 1H), 3.66 (s, 3H), 3.46–3.42 (m, 3H), 1.16 (d, *J* = 6.8 Hz, 6H). MS (ESI) *m*/*z*: [M + H]^+^: 518.3.

##### Methyl 4-((4-((5-chloro-4-((2-(isopropylsulfonyl)phenyl)amino)pyrimidin-2-yl)amino)phenyl)amino)-4-oxobutanoate (4b)

Purple solid. Yield: 36%. M.p. 202.2–204.9 °C. ^1^H NMR (600 MHz, DMSO-d_6_) δ 9.88 (s, 1H), 9.47 (d, *J* = 10.0 Hz, 2H), 8.59 (s, 1H), 8.27 (s, 1H), 7.85 (dd, *J* = 8.0, 1.5 Hz, 1H), 7.73 (t, *J* = 7.5 Hz, 1H), 7.50 (d, *J* = 8.4 Hz, 2H), 7.44 (d, *J* = 8.9 Hz, 2H), 7.42–7.37 (m, 1H), 3.60 (s, 3H), 3.44 (dt, *J* = 13.6, 6.8 Hz, 1H), 2.59 (s, 4H), 1.16 (d, *J* = 6.8 Hz, 6H). MS (ESI) *m*/*z*: [M + H]^+^: 532.3.

##### Methyl 5-((4-((5-chloro-4-((2-(isopropylsulfonyl)phenyl)amino)pyrimidin-2-yl)amino)phenyl)amino)-5-oxopentanoate (4c)

White solid. Yield: 30%. M.p. 201.7–202.3 °C. ^1^H NMR (600 MHz, DMSO-d_6_) δ 9.79 (s, 1H), 9.46 (d, *J* = 6.4 Hz, 2H), 8.59 (s, 1H), 8.26 (s, 1H), 7.85 (dd, *J* = 8.0, 1.5 Hz, 1H), 7.73 (t, *J* = 7.5 Hz, 1H), 7.50 (d, *J* = 8.4 Hz, 2H), 7.45 (d, *J* = 8.9 Hz, 2H), 7.40–7.37 (m, 1H), 3.59 (s, 3H), 3.43 (dd, *J* = 13.6, 6.8 Hz, 1H), 2.36 (t, *J* = 7.4 Hz, 2H), 2.32 (t, *J* = 7.4 Hz, 2H), 1.83 (p, *J* = 7.4 Hz, 2H), 1.16 (d, *J* = 6.8 Hz, 6H). MS (ESI) *m*/*z*: [M + H]^+^: 546.3.

##### Methyl 6-((4-((5-chloro-4-((2-(isopropylsulfonyl)phenyl)amino)pyrimidin-2-yl)amino)phenyl)amino)-6-oxohexanoate (4d)

Purple solid. Yield: 26%. M.p. 181.7–182.4 °C. ^1^H NMR (600 MHz, DMSO-d_6_) δ 9.77 (s, 1H), 9.47 (d, *J* = 5.7 Hz, 2H), 8.60 (s, 1H), 8.27 (s, 1H), 7.85 (dd, *J* = 8.0, 1.5 Hz, 1H), 7.73 (t, *J* = 7.4 Hz, 1H), 7.50 (d, *J* = 8.5 Hz, 2H), 7.45 (d, *J* = 8.9 Hz, 2H), 7.40–7.37 (m, 1H), 3.59 (s, 3H), 3.48–3.41 (m, 1H), 2.34 (t, *J* = 7.0 Hz, 2H), 2.29 (t, *J* = 6.9 Hz, 2H), 1.61–1.55 (m, 4H), 1.16 (d, *J* = 6.8 Hz, 6H). MS (ESI) *m*/*z*: [M + H]^+^: 560.3.

##### Ethyl 7-((4-((5-chloro-4-((2-(isopropylsulfonyl)phenyl)amino)pyrimidin-2-yl)amino)phenyl)amino)-7-oxoheptanoate (4e)

Brown solid. Yield: 54%. M.p. 174.5–176.8 °C. ^1^H NMR (600 MHz, DMSO-d_6_) δ 9.76 (s, 1H), 9.47 (s, 2H), 8.60 (s, 1H), 8.27 (s, 1H), 7.86 (dd, *J* = 8.0, 1.6 Hz, 1H), 7.73 (t, *J* = 7.3 Hz, 1H), 7.50 (d, *J* = 8.6 Hz, 2H), 7.46 (d, *J* = 9.0 Hz, 2H), 7.41–7.38 (m, 1H), 4.04 (t, *J* = 7.1 Hz, 2H), 3.49–3.41 (m, 1H), 2.28 (dt, *J* = 11.4, 7.4 Hz, 4H), 1.62–1.52 (m, 4H), 1.34–1.28 (m, 2H), 1.19–1.17 (m, 6H), 1.16 (d, *J* = 2.6 Hz, 3H). MS (ESI) *m*/*z*: [M + H]^+^: 588.3.

##### Methyl 8-((4-((5-chloro-4-((2-(isopropylsulfonyl)phenyl)amino)pyrimidin-2-yl)amino)phenyl)amino)-8-oxooctanoate (4f)

Yellow solid. Yield: 68%. M.p. 173.1–174.5 °C. ^1^H NMR (600 MHz, CDCl_3_) δ 9.62 (s, 1H), 8.55 (d, *J* = 7.9 Hz, 1H), 8.12 (s, 1H), 7.90 (dd, *J* = 7.9, 1.5 Hz, 1H), 7.64–7.60 (m, 1H), 7.46 (s, 4H), 7.25 (d, *J* = 5.0 Hz, 2H), 7.18 (s, 1H), 3.67 (s, 3H), 3.23 (dt, *J* = 13.7, 6.9 Hz, 1H), 2.30–2.36 (m, 4H), 1.77–1.72 (m, 2H), 1.66–1.62 (m, 2H), 1.43–1.36 (m, 4H), 1.31 (d, *J* = 6.9 Hz, 6H). MS (ESI) *m*/*z*: [M + H]^+^: 588.3.

##### Methyl 3-((3-((5-chloro-4-((2-(isopropylsulfonyl)phenyl)amino)pyrimidin-2-yl)amino)phenyl)amino)-3-oxopropanoate (4g)

White solid. Yield: 36%. M.p. 149.2–150.1 °C. ^1^H NMR (600 MHz, DMSO-d_6_) δ 10.13 (s, 1H), 9.59 (s, 1H), 9.55 (s, 1H), 8.70 (d, *J* = 7.5 Hz, 1H), 8.30 (d, *J* = 6.3 Hz, 1H), 7.86–7.81 (m, 2H), 7.72 (t, *J* = 7.3 Hz, 1H), 7.40–7.35 (m, 2H), 7.22–7.18 (m, 2H), 3.65 (d, *J* = 4.1 Hz, 3H), 3.47 (s, 2H), 3.46–3.43 (m, 1H), 1.18 (d, *J* = 6.8 Hz, 6H). MS (ESI) *m*/*z*: [M + H]^+^: 518.3.

##### Methyl 4-((3-((5-chloro-4-((2-(isopropylsulfonyl)phenyl)amino)pyrimidin-2-yl)amino)phenyl)amino)-4-oxobutanoate (4h)

Yellow solid. Yield: 24%. M.p. 101.2–103.5 °C. ^1^H NMR (600 MHz, DMSO-d_6_) δ 9.92 (s, 1H), 9.54 (d, *J* = 7.2 Hz, 2H), 8.70 (d, *J* = 7.2 Hz, 1H), 8.27 (s, 1H), 7.85–7.81 (m, 2H), 7.68 (t, *J* = 7.4 Hz, 1H), 7.36–7.32 (m, 2H), 7.18–7.14 (m, 2H), 3.58 (s, 3H), 3.44–3.41 (m, 1H), 2.60 (d, *J* = 5.7 Hz, 2H), 2.57 (d, *J* = 5.4 Hz, 2H), 1.17 (d, *J* = 6.8 Hz, 6H). MS (ESI) *m*/*z*: [M + H]^+^: 532.3.

##### Methyl 5-((3-((5-chloro-4-((2-(isopropylsulfonyl)phenyl)amino)pyrimidin-2-yl)amino)phenyl)amino)-5-oxopentanoate (4i)

Yellow solid. Yield: 60%. M.p. 129.8–130.1 °C. ^1^H NMR (600 MHz, DMSO-d_6_) δ 9.84 (s, 1H), 9.56 (s, 2H), 8.71 (d, *J* = 6.4 Hz, 1H), 8.28 (s, 1H), 7.89–7.82 (m, 2H), 7.68 (t, *J* = 7.6 Hz, 1H), 7.35 (t, *J* = 7.5 Hz, 2H), 7.21–7.14 (m, 2H), 3.59 (s, 3H), 3.46–3.43 (m, 1H), 2.37–2.34 (m, 4H), 1.86–1.79 (m, 2H), 1.18 (d, *J* = 6.8 Hz, 6H). MS (ESI) *m*/*z*: [M + H]^+^: 546.3.

##### Methyl 6-((3-((5-chloro-4-((2-(isopropylsulfonyl)phenyl)amino)pyrimidin-2-yl)amino)phenyl)amino)-6-oxohexanoate (4j)

White solid. Yield: 26%. M.p. 128.9–131.5 °C. ^1^H NMR (600 MHz, DMSO-d_6_) δ 9.81 (s, 1H), 9.55 (s, 2H), 8.70 (d, *J* = 7.4 Hz, 1H), 8.29 (s, 1H), 7.85–7.82 (m, 2H), 7.68 (t, *J* = 7.4 Hz, 1H), 7.36 (d, *J* = 7.3 Hz, 1H), 7.33 (d, *J* = 7.8 Hz, 1H), 7.19 (d, *J* = 8.1 Hz, 1H), 7.17–7.14 (m, 1H), 3.58 (d, *J* = 4.0 Hz, 3H), 3.47–3.43 (m, 1H), 2.33 (d, *J* = 6.7 Hz, 2H), 2.30 (d, *J* = 5.1 Hz, 2H), 1.58–1.53 (m, 4H), 1.17 (d, *J* = 6.8 Hz, 6H). MS (ESI) *m*/*z*: [M + H]^+^: 560.0.

##### Ethyl 7-((3-((5-chloro-4-((2-(isopropylsulfonyl)phenyl)amino)pyrimidin-2-yl)amino)phenyl)amino)-7-oxoheptanoate (4k)

Yellow solid. Yield: 38%. M.p. 128.4–129.2 °C. ^1^H NMR (600 MHz, DMSO-d_6_) δ 9.80 (s, 1H), 9.55 (d, *J* = 7.1 Hz, 2H), 8.71 (d, *J* = 6.2 Hz, 1H), 8.28 (s, 1H), 7.87–7.81 (m, 2H), 7.68 (t, *J* = 7.5 Hz, 1H), 7.35–7.33 (m, 2H), 7.19–7.15 (m, 2H), 4.03 (q, *J* = 7.0 Hz, 2H), 3.47–3.44 (m, 1H), 2.28 (t, *J* = 5.8 Hz, 4H), 1.57–1.54 (m, 4H), 1.36–1.23 (m, 3H), 1.17 (d, *J* = 6.6 Hz, 6H), 1.15 (s, 2H). MS (ESI) *m*/*z*: [M + H]^+^: 588.4.

##### Methyl 8-((3-((5-chloro-4-((2-(isopropylsulfonyl)phenyl)amino)pyrimidin-2-yl)amino)phenyl)amino)-8-oxooctanoate (4l)

White solid. Yield: 32%. M.p. 152.2–154.8 °C. ^1^H NMR (600 MHz, DMSO-d_6_) δ 9.79 (s, 1H), 9.55 (d, *J* = 11.5 Hz, 2H), 8.71 (d, *J* = 7.7 Hz, 1H), 8.29 (s, 1H), 7.89–7.81 (m, 2H), 7.68 (t, *J* = 7.7 Hz, 1H), 7.38–7.30 (m, 2H), 7.21–7.12 (m, 2H), 3.58 (s, 3H), 3.45 (dt, *J* = 13.5, 6.8 Hz, 1H), 2.30–2.22 (m, 4H), 1.58–1.50 (m, 4H), 1.29 (dt, *J* = 6.9, 3.5 Hz, 4H), 1.18 (d, *J* = 6.8 Hz, 6H). MS (ESI) *m*/*z*: [M + H]^+^: 588.4.

#### General procedure for the synthesis of intermediates 5a–l

Sodium hydroxide (0.10 g, 2.50 mmol) was dissolved in methanol/water (80%, 10 mL) mixture and heated to 70 °C, compound **4a** (0.40 g, 0.77 mmol) was slowly added to the solution and stirred under reflux for 6 h. When the reaction completed, the solvent was evaporated *in vacuo*, and water (40 mL) was added, after stirring for 0.5 h at room temperature. The reaction mixture was filtered, dried in an oven to give intermediate **5a** which can be directly used. **5b–l** were synthesised in the manner of **5a**.

#### General procedure for the synthesis of 6a–l

##### N^1^-(2-aminophenyl)-N^3^-(4-((5-chloro-4-((2-(isopropylsulfonyl)phenyl)amino)pyrimidin-2-yl)amino)phenyl)malonamide (6a)

Synthesised using the preparation method of **4a** using **5a** (0.30 g, 0.59 mmol), *o*-phenylenediamine (0.06 g, 0.52 mmol), HOBT (0.17 g, 1.2 mmol), EDCI (0.23 g, 1.17 mmol) and DIPEA (0.31 g, 2.37 mmol) in 6 mL DMF, and 0.07 g of **6a** was gained as white solid. Yield: 25%. M.p. 210.0–212.2 °C. ^1^H NMR (600 MHz, DMSO-d_6_) δ 10.10 (s, 1H), 9.52 (s, 1H), 9.47 (s, 1H), 9.34 (s, 1H), 8.60 (s, 1H), 8.28 (s, 1H), 7.86 (dd, *J* = 8.0, 1.5 Hz, 1H), 7.75 (t, *J* = 7.8 Hz, 1H), 7.54 (d, *J* = 8.4 Hz, 2H), 7.48 (d, *J* = 8.9 Hz, 2H), 7.40 (dd, *J* = 11.6, 4.4 Hz, 1H), 7.14 (dd, *J* = 7.8, 1.1 Hz, 1H), 6.95–6.91 (m, 1H), 6.72 (dd, *J* = 8.0, 1.2 Hz, 1H), 6.57–6.52 (m, 1H), 4.97 (s, 2H), 3.48–3.41 (m, 3H), 1.17 (d, *J* = 6.8 Hz, 6H). ^13 ^C NMR (151 MHz, DMSO-d_6_) δ 166.15, 166.07, 158.15, 155.75, 155.36, 143.08, 138.51, 136.13, 135.26, 133.82, 131.41, 126.85, 126.40, 125.13, 124.65, 124.16, 123.21, 120.57, 119.98, 116.40, 115.94, 104.92, 55.33, 45.41, 15.33. HRMS *m*/*z* calcd for C_28_H_29_ClN_7_O_4_S [M + H]^+^: 594.1690, found: 549.1685.

##### N^1^-(2-aminophenyl)-N^4^-(4-((5-chloro-4-((2-(isopropylsulfonyl)phenyl)amino)pyrimidin-2-yl)amino)phenyl)succinimide (6b)

Yellow solid. Yield: 68%. M.p. 215.4–217.9 °C. ^1^H NMR (600 MHz, DMSO-d_6_) δ 9.89 (s, 1H), 9.47 (d, *J* = 6.2 Hz, 2H), 9.17 (s, 1H), 8.60 (s, 1H), 8.27 (s, 1H), 7.85 (dd, *J* = 8.0, 1.5 Hz, 1H), 7.73 (t, *J* = 7.4 Hz, 1H), 7.51 (d, *J* = 8.2 Hz, 2H), 7.46 (d, *J* = 8.9 Hz, 2H), 7.38 (t, *J* = 7.7 Hz, 1H), 7.13 (d, *J* = 6.9 Hz, 1H), 6.91–6.87 (m, 1H), 6.70 (dd, *J* = 7.9, 1.0 Hz, 1H), 6.54–6.50 (m, 1H), 4.88 (s, 2H), 3.45 (dt, *J* = 13.6, 6.8 Hz, 1H), 2.64 (s, 4H), 1.16 (d, *J* = 6.8 Hz, 6H). ^13 ^C NMR (151 MHz, DMSO-d_6_) δ 170.93, 170.60, 158.18, 155.73, 155.33, 142.71, 138.52, 135.68, 135.26, 134.31, 131.40, 126.36, 126.08, 125.00, 124.56, 124.11, 123.77, 120.59, 119.77, 116.47, 116.09, 104.86, 55.34, 32.01, 31.28, 15.33. HRMS *m*/*z* calcd for C_29_H_31_ClN_7_O_4_S [M + H]^+^: 608.1847, found: 608.1843.

##### N^1^-(2-aminophenyl)-N^5^-(4-((5-chloro-4-((2-(isopropylsulfonyl)phenyl)amino)pyrimidin-2-yl)amino)phenyl)glutaramide (6c)

White solid. Yield: 47%. M.p. 159.3–161.2 °C. ^1^H NMR (600 MHz, DMSO-d_6_) δ 9.82 (s, 1H), 9.47 (d, *J* = 6.6 Hz, 2H), 9.10 (s, 1H), 8.60 (s, 1H), 8.27 (s, 1H), 7.85 (dd, *J* = 8.0, 1.5 Hz, 1H), 7.73 (t, *J* = 7.4 Hz, 1H), 7.46–7.52 (m, 4H), 7.41–7.37 (m, 1H), 7.18 (dd, *J* = 7.8, 1.0 Hz, 1H), 6.91–6.86 (m, 1H), 6.71 (dd, *J* = 8.0, 1.2 Hz, 1H), 6.56–6.51 (m, 1H), 4.83 (s, 2H), 3.42–3.46 (m, 1H), 2.40–2.33 (m, 4H), 1.94–1.88 (m, 2H), 1.16 (d, *J* = 6.8 Hz, 6H). ^13 ^C NMR (151 MHz, DMSO-d_6_) δ 171.23, 170.92, 158.18, 155.74, 155.34, 142.38, 138.52, 135.70, 135.25, 134.32, 131.41, 126.19, 125.85, 125.01, 124.66, 124.13, 123.96, 120.52, 119.86, 116.59, 116.31, 104.84, 55.33, 36.06, 35.50, 21.73, 15.33. HRMS *m*/*z* calcd for C_30_H_33_ClN_7_O_4_S [M + H]^+^: 622.2003, found: 622.2008.

##### N^1^-(2-aminophenyl)-N^6-^(4-((5-chloro-4-((2-(isopropylsulfonyl)phenyl)amino)pyrimidin-2-yl)amino)phenyl)adipamide (6d)

White solid. Yield: 34%. M.p. 209.8–211.1 °C. ^1^H NMR (600 MHz, DMSO-d_6_) δ 9.80 (s, 1H), 9.48 (s, 1H), 9.46 (s, 1H), 9.11 (s, 1H), 8.59 (s, 1H), 8.27 (s, 1H), 7.84 (dd, *J* = 8.0, 1.4 Hz, 1H), 7.73 (t, *J* = 7.6 Hz, 1H), 7.50 (d, *J* = 8.1 Hz, 2H), 7.46 (d, *J* = 8.9 Hz, 2H), 7.38 (t, *J* = 7.6 Hz, 1H), 7.16–7.13 (m, 1H), 6.90–6.87 (m, 1H), 6.71 (dd, *J* = 7.9, 1.0 Hz, 1H), 6.54–6.51 (m, 1H), 4.83 (s, 2H), 3.48–3.40 (m, 1H), 2.35 (s, 2H), 2.32 (s, 2H), 1.64 (d, *J* = 3.1 Hz, 4H), 1.16 (d, *J* = 6.8 Hz, 6H). ^13 ^C NMR (151 MHz, DMSO-d_6_) δ 171.50, 171.18, 158.19, 155.73, 155.32, 142.38, 138.52, 135.70, 135.24, 134.34, 131.40, 126.19, 125.81, 124.95, 124.49, 124.08, 124.02, 120.56, 119.87, 116.65, 116.37, 104.85, 55.35, 36.69, 36.15, 25.58, 25.43, 15.33. HRMS *m*/*z* calcd for C_31_H_35_ClN_7_O_4_S [M + H]^+^: 636.2160, found: 636.2157.

##### N^1^-(2-aminophenyl)-N^7^-(4-((5-chloro-4-((2-(isopropylsulfonyl)phenyl)amino)pyrimidin-2-yl)amino)phenyl)heptanediamide (6e)

White solid. Yield: 40%. M.p. 215.4–216.9 °C. ^1^H NMR (600 MHz, DMSO-d_6_) δ 9.77 (s, 1H), 9.47 (d, *J* = 4.5 Hz, 2H), 9.08 (s, 1H), 8.60 (s, 1H), 8.27 (s, 1H), 7.85 (dd, *J* = 8.0, 1.6 Hz, 1H), 7.73 (t, *J* = 7.4 Hz, 1H), 7.50 (d, *J* = 8.5 Hz, 2H), 7.46 (d, *J* = 9.0 Hz, 2H), 7.41–7.36 (m, 1H), 7.15 (dd, *J* = 7.8, 1.2 Hz, 1H), 6.90–6.86 (m, 1H), 6.71 (dd, *J* = 8.0, 1.3 Hz, 1H), 6.58–6.49 (m, 1H), 4.81 (s, 2H), 3.45 (dt, *J* = 13.6, 6.8 Hz, 1H), 2.39–2.29 (m, 4H), 1.67–1.60 (m, 4H), 1.40–1.33 (m, 2H), 1.17 (d, *J* = 6.8 Hz, 6H). ^13 ^C NMR (151 MHz, DMSO-d_6_) δ 171.60, 171.26, 158.19, 155.73, 155.32, 142.35, 138.52, 135.67, 135.24, 134.36, 131.40, 126.15, 125.76, 124.97, 124.53, 124.08, 124.05, 120.55, 119.84, 116.64, 116.36, 104.86, 55.34, 36.70, 36.14, 28.84, 25.61, 25.48, 15.33. HRMS *m*/*z* calcd for C_32_H_37_ClN_7_O_4_S [M + H]^+^: 650.2316, found: 650.2312.

##### N^1^-(2-aminophenyl)-N^8^-(4-((5-chloro-4-((2-(isopropylsulfonyl)phenyl)amino)pyrimidin-2-yl)amino)phenyl)octanediamide (6f)

Yellow solid. Yield: 60%. M.p. 201.2–203.8 °C. ^1^H NMR (600 MHz, DMSO-d_6_) δ 9.76 (s, 1H), 9.46 (s, 2H), 9.08 (s, 1H), 8.59 (s, 1H), 8.26 (s, 1H), 7.84 (dd, *J* = 8.0, 1.6 Hz, 1H), 7.73 (t, *J* = 7.3 Hz, 1H), 7.42–7.55 (m, 4H), 7.41–7.35 (m, 1H), 7.14 (dd, *J* = 7.8, 1.2 Hz, 1H), 6.91–6.84 (m, 1H), 6.70 (dd, *J* = 8.0, 1.3 Hz, 1H), 6.59 − 6.50 (m, 1H), 4.80 (s, 2H), 3.41–4.46 (m, 1H), 2.23–2.35 (m, 4H), 1.64–1.54 (m, 4H), 1.34 (dd, *J* = 6.9, 3.4 Hz, 4H), 1.16 (d, *J* = 6.8 Hz, 6H). ^13 ^C NMR (151 MHz, DMSO-d_6_) δ 171.61, 171.27, 158.18, 155.74, 155.33, 142.35, 138.52, 135.66, 135.24, 134.36, 131.40, 126.15, 125.75, 125.00, 124.54, 124.10, 124.06, 120.54, 119.83, 116.65, 116.36, 104.85, 55.33, 36.79, 36.23, 29.02, 29.00, 25.71, 25.60, 15.33. HRMS *m*/*z* calcd for C_33_H_39_ClN_7_O_4_S [M + H]^+^: 664.2473, found: 664.2477.

##### N^1^-(2-aminophenyl)-N^3^-(3-((5-chloro-4-((2-(isopropylsulfonyl)phenyl)amino)pyrimidin-2-yl)amino)phenyl)malonamide (6g)

White solid. Yield: 54%. M.p. 135.4–137.2 °C. ^1^H NMR (600 MHz, DMSO-d_6_) δ 10.14 (s, 1H), 9.60 (s, 1H), 9.55 (s, 1H), 9.35 (s, 1H), 8.70 (d, *J* = 6.7 Hz, 1H), 8.30 (s, 1H), 7.84 (d, *J* = 8.2 Hz, 2H), 7.73 (t, *J* = 7.8 Hz, 1H), 7.42–7.34 (m, 2H), 7.25 (d, *J* = 8.0 Hz, 1H), 7.20 (t, *J* = 8.0 Hz, 1H), 7.14 (d, *J* = 7.8 Hz, 1H), 6.93 (t, *J* = 7.7 Hz, 1H), 6.72 (d, *J* = 7.9 Hz, 1H), 6.54 (t, *J* = 7.5 Hz, 1H), 4.96 (s, 2H), 3.48 (s, 2H), 3.46–4.43 (m, 1H), 1.17 (d, *J* = 6.8 Hz, 6H). ^13 ^C NMR (151 MHz, DMSO-d_6_) δ 166.36, 166.12, 158.15, 155.59, 155.25, 143.08, 140.80, 139.47, 138.47, 135.44, 131.39, 129.06, 126.85, 126.39, 124.50, 124.09, 123.96, 123.18, 116.40, 115.94, 115.82, 113.84, 111.55, 105.47, 55.39, 45.47, 15.33. HRMS *m*/*z* calcd for C_28_H_29_ClN_7_O_4_S [M + H]^+^: 594.1690, found: 594.1684.

##### N^1^-(2-aminophenyl)-N^4^-(3-((5-chloro-4-((2-(isopropylsulfonyl)phenyl)amino)pyrimidin-2-yl)amino)phenyl)succinimide (6h)

Yellow solid. Yield: 39%. M.p. 135.9–138.6 °C. ^1^H NMR (600 MHz, DMSO-d_6_) δ 9.93 (s, 1H), 9.55 (d, *J* = 1.5 Hz, 2H), 9.16 (s, 1H), 8.70 (d, *J* = 7.2 Hz, 1H), 8.29 (s, 1H), 7.84–7.81 (m, 2H), 7.71 (t, *J* = 7.6 Hz, 1H), 7.36 (d, *J* = 7.5 Hz, 1H), 7.33 (d, *J* = 8.2 Hz, 1H), 7.22 (d, *J* = 8.1 Hz, 1H), 7.16 (t, *J* = 8.0 Hz, 1H), 7.13 (d, *J* = 7.7 Hz, 1H), 6.88 (dd, *J* = 11.1, 4.2 Hz, 1H), 6.69 (d, *J* = 7.9 Hz, 1H), 6.51 (dd, *J* = 11.0, 4.1 Hz, 1H), 4.86 (s, 2H), 3.45 (dt, *J* = 13.6, 6.8 Hz, 1H), 2.67–2.63 (m, 4H), 1.18–1.16 (m, 6H). ^13 ^C NMR (151 MHz, DMSO-d_6_) δ 170.90, 170.88, 158.19, 155.61, 155.21, 142.67, 140.67, 139.88, 138.49, 135.42, 131.36, 128.92, 126.31, 126.00, 124.40, 124.04, 123.91, 123.77, 116.44, 116.08, 115.51, 113.83, 111.61, 105.37, 55.41, 32.05, 31.16, 15.34. HRMS *m*/*z* calcd for C_29_H_31_ClN_7_O_4_S [M + H]^+^: 608.1847, found: 608.1845.

##### N^1^-(2-aminophenyl)-N^5^-(3-((5-chloro-4-((2-(isopropylsulfonyl)phenyl)amino)pyrimidin-2-yl)amino)phenyl)glutaramide (6i)

Yellow solid. Yield: 43%. M.p. 200.6–201.7 °C. ^1^H NMR (600 MHz, DMSO-d_6_) δ 9.87 (s, 1H), 9.56 (s, 2H), 9.11 (s, 1H), 8.71 (d, *J* = 6.9 Hz, 1H), 8.29 (s, 1H), 7.87 (s, 1H), 7.84–7.81 (m, 1H), 7.69 (t, *J* = 7.6 Hz, 1H), 7.37–7.34 (m, 2H), 7.23 (d, *J* = 8.1 Hz, 1H), 7.20–7.17 (m, 2H), 6.91–6.87 (m, 1H), 6.72 (dd, *J* = 8.0, 1.2 Hz, 1H), 6.56–6.51 (m, 1H), 4.84 (s, 2H), 3.47–3.45 (m, 1H), 2.40–2.37 (m, 4H), 1.93–1.87 (m, 2H), 1.17 (d, *J* = 6.8 Hz, 6H). ^13 ^C NMR (151 MHz, DMSO-d_6_) δ 171.23, 158.19, 155.62, 155.20, 142.37, 140.66, 139.90, 138.51, 135.35, 131.37, 128.89, 126.19, 125.84, 124.38, 124.00, 123.97, 123.87, 116.60, 116.32, 115.54, 113.90, 111.71, 105.37, 55.42, 36.14, 35.51, 21.71, 15.34. HRMS *m*/*z* calcd for C_30_H_33_ClN_7_O_4_S [M + H]^+^: 622.2003, found: 622.2001.

##### N^1^-(2-aminophenyl)-N^6^-(3-((5-chloro-4-((2-(isopropylsulfonyl)phenyl)amino)pyrimidin-2-yl)amino)phenyl)adipamide (6j)

White solid. Yield: 39%. M.p. 145.8–147.6 °C. ^1^H NMR (600 MHz, DMSO-d_6_) δ 9.83 (s, 1H), 9.55 (d, *J* = 4.2 Hz, 2H), 9.10 (s, 1H), 8.71 (d, *J* = 7.4 Hz, 1H), 8.29 (s, 1H), 7.86–7.82 (m, 2H), 7.69 (t, *J* = 7.6 Hz, 1H), 7.36 (d, *J* = 7.5 Hz, 1H), 7.33 (d, *J* = 8.5 Hz, 1H), 7.20 (d, *J* = 8.1 Hz, 1H), 7.18–7.14 (m, 2H), 6.90–6.87 (m, 1H), 6.71 (dd, *J* = 7.9, 1.0 Hz, 1H), 6.59–6.50 (m, 1H), 4.81 (s, 2H), 3.45 (dd, *J* = 13.6, 6.8 Hz, 1H), 2.34 (d, *J* = 5.4 Hz, 4H), 1.63 (s, 4H), 1.18 (d, *J* = 6.8 Hz, 6H). ^13 ^C NMR (151 MHz, DMSO-d_6_) δ 171.46, 158.19, 155.62, 155.19, 142.37, 140.66, 139.89, 138.51, 135.35, 131.37, 128.90, 126.18, 125.78, 124.37, 124.00, 123.86, 116.63, 116.35, 115.53, 113.88, 111.70, 105.36, 55.41, 36.74, 36.13, 32.01, 29.90, 25.56, 25.38, 15.34. HRMS *m*/*z* calcd for C_31_H_35_ClN_7_O_4_S [M + H]^+^: 636.2160, found: 636.2161.

##### N^1^-(2-aminophenyl)-N^7^-(3-((5-chloro-4-((2-(isopropylsulfonyl)phenyl)amino)pyrimidin-2-yl)amino)phenyl)heptanediamide (6k)

Yellow solid. Yield: 30%. M.p. 108.4–109.8 °C. ^1^H NMR (600 MHz, CDCl_3_) δ 9.52 (s, 1H), 8.50 (d, *J* = 8.3 Hz, 1H), 8.07 (s, 1H), 8.02 (s, 1H), 7.87 (s, 1H), 7.79 (d, *J* = 7.9 Hz, 1H), 7.62 (s, 1H), 7.50–7.45 (m, 2H), 7.28 (d, *J* = 7.3 Hz, 1H), 7.12 (t, *J* = 7.2 Hz, 1H), 7.09–7.04 (m, 3H), 6.88 (t, *J* = 7.6 Hz, 1H), 6.63–6.59 (m, 2H), 5.22 (s, 2H), 3.15–3.13 (m, 1H), 2.29 (t, *J* = 6.6 Hz, 2H), 2.22 (t, *J* = 6.5 Hz, 2H), 1.61–1.58 (m, 4H), 1.30 (d, *J* = 4.9 Hz, 2H), 1.21 (d, *J* = 6.7 Hz, 6H). ^13 ^C NMR (151 MHz, DMSO-d_6_) δ 171.58, 171.55, 158.19, 155.62, 155.19, 142.30, 140.64, 139.93, 138.51, 135.35, 131.37, 128.89, 126.12, 125.73, 124.35, 124.07, 123.98, 123.84, 116.65, 116.35, 115.50, 113.87, 111.68, 105.36, 55.41, 36.73, 36.13, 28.83, 25.61, 25.43, 15.34. HRMS *m*/*z* calcd for C_32_H_37_ClN_7_O_4_S [M + H]^+^: 650.2316, found: 650.2309.

##### N^1^-(2-aminophenyl)-N^8^-(3-((5-chloro-4-((2-(isopropylsulfonyl)phenyl)amino)pyrimidin-2-yl)amino)phenyl)octanediamide (6l)

Yellow solid. Yield: 89%. M.p. 105.2–107.3 °C. ^1^H NMR (600 MHz, DMSO-d_6_) δ 9.80 (s, 1H), 9.55 (d, *J* = 7.9 Hz, 2H), 9.09 (s, 1H), 8.71 (d, *J* = 7.5 Hz, 1H), 8.29 (s, 1H), 7.86–7.81 (m, 2H), 7.68 (t, *J* = 7.6 Hz, 1H), 7.38–7.32 (m, 2H), 7.20–7.13 (m, 3H), 6.90–6.86 (m, 1H), 6.71 (dd, *J* = 8.0, 1.2 Hz, 1H), 6.55–6.51 (m, 1H), 4.86 (s, 2H), 3.47–3.43 (m, 1H), 2.34–2.26 (m, 4H), 1.63–1.58 (m, 4H), 1.35–1.32 (m, 4H), 1.17 (d, *J* = 6.8 Hz, 6H). ^13 ^C NMR (151 MHz, DMSO-d_6_) δ 171.67, 171.62, 158.19, 155.63, 155.19, 142.31, 140.63, 139.90, 138.50, 135.35, 131.38, 128.91, 126.19, 125.77, 124.34, 124.06, 123.98, 123.86, 116.71, 116.40, 115.54, 113.91, 111.76, 105.35, 55.43, 36.82, 36.21, 32.00, 28.99, 25.70, 25.54, 15.32. HRMS *m*/*z* calcd for C_33_H_39_ClN_7_O_4_S [M + H]^+^: 664.2473, found: 664.2477.

#### General procedure for the synthesis of intermediates 8a–b

Synthesised using the preparation method of **3a** using **1** (1.50 g, 4.30 mmol), p-aminobenzoic acid (0.89 g, 6.50 mmol) in 40 mL absolute ethanol followed by the addition of the HCl solution (37%, 0.8 mL), and 1.70 g of **8a** was gained as white solid. **8b** was synthesised in the manner of **8a**.

#### General procedure for the synthesis of intermediates 9a–j

##### Methyl 3–(4-((5-chloro-4-((2-(isopropylsulfonyl)phenyl)amino)pyrimidin-2-yl)amino)benzamido)propanoate (9a)

Compounds **9a–j** was synthesised according to the preparation procedure of **4a** using **8a** (1.00 g, 2.24 mmol), methyl 3-aminopropionate (0.47 g, 3.36 mmol), HOBT (0.66 g, 4.89 mmol), EDCI (0.92 g, 4.80 mmol) and DIPEA (1.22 g, 9.46 mmol) in 10 mL DMF, and 0.91 g of **9a** was gained as white solid. Yield: 77%. M.p. 155.3–157.2 °C. ^1^H NMR (600 MHz, DMSO-d_6_) δ 9.82 (s, 1H), 9.48 (s, 1H), 8.56 (d, *J* = 7.3 Hz, 1H), 8.38 (t, *J* = 5.5 Hz, 1H), 8.35 (s, 1H), 7.88 (dd, *J* = 8.0, 1.3 Hz, 1H), 7.83–7.80 (m, 1H), 7.72 (d, *J* = 8.9 Hz, 2H), 7.69 (d, *J* = 9.0 Hz, 2H), 7.44 (t, *J* = 7.6 Hz, 1H), 3.61 (s, 3H), 3.49–3.45 (m, 3H), 2.59 (t, *J* = 7.0 Hz, 2H), 1.16 (d, *J* = 6.8 Hz, 6H). MS (ESI) *m*/*z*: [M + H]^+^: 532.3.

##### Methyl 4–(4-((5-chloro-4-((2-(isopropylsulfonyl)phenyl)amino)pyrimidin-2-yl)amino)benzamido)butanoate (9b)

White solid. Yield: 47%. M.p. 195.2–196.0 °C. ^1^H NMR (600 MHz, DMSO-d_6_) δ 9.81 (s, 1H), 9.48 (s, 1H), 8.56 (d, *J* = 7.3 Hz, 1H), 8.35 (s, 1H), 8.30 (t, *J* = 5.6 Hz, 1H), 7.88 (dd, *J* = 7.9, 1.5 Hz, 1H), 7.85–7.79 (m, 1H), 7.73 (s, 1H), 7.72 (s, 1H), 7.70 (s, 1H), 7.68 (s, 1H), 7.44 (t, *J* = 7.6 Hz, 1H), 3.58 (s, 3H), 3.50–3.42 (m, 1H), 3.28–3.23 (m, 2H), 2.36 (t, *J* = 7.4 Hz, 2H), 1.80–1.75 (m, 2H), 1.16 (d, *J* = 6.8 Hz, 6H). MS (ESI) *m*/*z*: [M + H]^+^: 546.3.

##### Methyl 5–(4-((5-chloro-4-((2-(isopropylsulfonyl)phenyl)amino)pyrimidin-2-yl)amino)benzamido)pentanoate (9c)

Yellow solid. Yield: 68%. M.p. 167.4–169.2 °C. ^1^H NMR (600 MHz, DMSO-d_6_) δ 9.81 (s, 1H), 9.49 (s, 1H), 8.57 (d, *J* = 6.7 Hz, 1H), 8.35 (s, 1H), 8.28 (t, *J* = 5.6 Hz, 1H), 7.88 (dd, *J* = 7.9, 1.2 Hz, 1H), 7.84–7.80 (m, 1H), 7.73 (d, *J* = 8.8 Hz, 2H), 7.70 (d, *J* = 8.7 Hz, 2H), 7.44 (t, *J* = 7.6 Hz, 1H), 3.58 (s, 3H), 3.49–3.42 (m, 1H), 3.25 (dd, *J* = 12.4, 6.4 Hz, 2H), 2.34 (t, *J* = 7.2 Hz, 2H), 1.60–1.55 (m, 2H), 1.55–1.50 (m, 2H), 1.17 (d, *J* = 6.8 Hz, 6H). MS (ESI) *m*/*z*: [M + H]^+^: 560.3.

##### Methyl 6–(4-((5-chloro-4-((2-(isopropylsulfonyl)phenyl)amino)pyrimidin-2-yl)amino)benzamido)hexanoate (9d)

White solid. Yield: 59%. M.p. 181.3–182.6 °C. ^1^H NMR (600 MHz, DMSO-d_6_) δ 9.81 (s, 1H), 9.49 (s, 1H), 8.57 (d, *J* = 7.2 Hz, 1H), 8.36 (s, 1H), 8.26 (t, *J* = 5.6 Hz, 1H), 7.89 (dd, *J* = 8.0, 1.5 Hz, 1H), 7.84–7.80 (m, 1H), 7.74 (s, 1H), 7.72 (s, 1H), 7.71 (s, 1H), 7.69 (s, 1H), 7.47–7.43 (m, 1H), 3.58 (s, 3H), 3.50–3.43 (m, 1H), 3.25–3.21 (m, 2H), 2.31 (t, *J* = 7.4 Hz, 2H), 1.59–1.54 (m, 2H), 1.54–1.49 (m, 2H), 1.35–1.28 (m, 2H), 1.17 (d, *J* = 6.8 Hz, 6H). MS (ESI) *m*/*z*: [M + H]^+^: 574.3.

##### Methyl 7–(4-((5-chloro-4-((2-(isopropylsulfonyl)phenyl)amino)pyrimidin-2-yl)amino)benzamido)heptanoate (9e)

White solid. Yield: 76%. M.p. 154.1–156.8 °C. ^1^H NMR (600 MHz, DMSO-d_6_) δ 9.81 (s, 1H), 9.49 (s, 1H), 8.57 (d, *J* = 7.1 Hz, 1H), 8.36 (s, 1H), 8.25 (t, *J* = 5.6 Hz, 1H), 7.89 (dd, *J* = 7.9, 1.3 Hz, 1H), 7.85–7.80 (m, 1H), 7.73 (d, *J* = 8.8 Hz, 2H), 7.70 (d, *J* = 8.7 Hz, 2H), 7.45 (t, *J* = 7.6 Hz, 1H), 3.58 (s, 3H), 3.49–3.46 (m, 1H), 3.25–3.21 (m, 2H), 2.30 (t, *J* = 7.4 Hz, 2H), 1.55–1.51 (m, 4H), 1.30 (t, *J* = 3.5 Hz, 4H), 1.17 (d, *J* = 6.8 Hz, 6H). MS (ESI) *m*/*z*: [M + H]^+^: 588.3.

##### Methyl 3–(3-((5-chloro-4-((2-(isopropylsulfonyl)phenyl)amino)pyrimidin-2-yl)amino)benzamido)propanoate (9f)

White solid. Yield: 49%. M.p. 152.0–153.7 °C. ^1^H NMR (600 MHz, DMSO-d_6_) δ 9.69 (s, 1H), 9.57 (s, 1H), 8.65 (d, *J* = 6.4 Hz, 1H), 8.46 (t, *J* = 5.5 Hz, 1H), 8.32 (d, *J* = 6.4 Hz, 1H), 8.03 (s, 1H), 7.84 (dd, *J* = 8.0, 1.5 Hz, 1H), 7.77 (d, *J* = 7.6 Hz, 1H), 7.68 (t, *J* = 7.6 Hz, 1H), 7.41 (d, *J* = 7.8 Hz, 1H), 7.38–7.34 (m, 2H), 3.60 (s, 3H), 3.48 (dd, *J* = 10.7, 5.1 Hz, 2H), 3.47–3.43 (m, 1H), 2.57 (t, *J* = 7.0 Hz, 2H), 1.17 (d, *J* = 6.8 Hz, 6H). MS (ESI) *m*/*z*: [M + H]^+^: 532.3.

##### Methyl 4–(3-((5-chloro-4-((2-(isopropylsulfonyl)phenyl)amino)pyrimidin-2-yl)amino)benzamido)butanoate (9g)

White solid. Yield: 35%. M.p. 149.3–150.8 °C. ^1^H NMR (600 MHz, DMSO-d_6_) δ 9.69 (s, 1H), 9.57 (s, 1H), 8.66 (d, *J* = 6.3 Hz, 1H), 8.40 (t, *J* = 5.6 Hz, 1H), 8.33 (s, 1H), 8.04 (s, 1H), 7.84 (dd, *J* = 8.0, 1.5 Hz, 1H), 7.77 (d, *J* = 7.6 Hz, 1H), 7.67 (t, *J* = 7.6 Hz, 1H), 7.43 (d, *J* = 7.7 Hz, 1H), 7.37–7.33 (m, 2H), 3.58 (s, 3H), 3.47–3.44 (m, 1H), 3.28–3.25 (m, 2H), 2.36 (t, *J* = 7.4 Hz, 2H), 1.76 (p, *J* = 7.2 Hz, 2H), 1.17 (d, *J* = 6.8 Hz, 6H). MS (ESI) *m*/*z*: [M + H]^+^: 546.3.

##### Methyl 5–(3-((5-chloro-4-((2-(isopropylsulfonyl)phenyl)amino)pyrimidin-2-yl)amino)benzamido)pentanoate (9h)

Yellow solid. Yield: 47%. M.p. 75.4–77.1 °C. ^1^H NMR (600 MHz, DMSO-d_6_) δ 9.68 (s, 1H), 9.57 (s, 1H), 8.66 (d, *J* = 6.3 Hz, 1H), 8.37 (t, *J* = 5.6 Hz, 1H), 8.32 (s, 1H), 8.03 (s, 1H), 7.84 (dd, *J* = 7.9, 1.4 Hz, 1H), 7.76 (d, *J* = 7.5 Hz, 1H), 7.67 (t, *J* = 7.7 Hz, 1H), 7.43 (d, *J* = 7.7 Hz, 1H), 7.35 (dd, *J* = 8.6, 4.8 Hz, 1H), 7.33 (t, *J* = 5.9 Hz, 1H), 3.58 (s, 3H), 3.47–3.44 (m, 1H), 3.26–3.23 (m, 2H), 2.33 (t, *J* = 7.2 Hz, 2H), 1.59–1.53 (m, 2H), 1.53–1.49 (m, 2H), 1.17 (d, *J* = 6.8 Hz, 6H). MS (ESI) *m*/*z*: [M + H]^+^: 560.3.

##### Methyl 6–(3-((5-chloro-4-((2-(isopropylsulfonyl)phenyl)amino)pyrimidin-2-yl)amino)benzamido)hexanoate (9i)

Yellow solid. Yield: 60%. M.p. 99.6–101.5 °C. ^1^H NMR (600 MHz, CDCl_3_) δ 9.66 (s, 1H), 8.58 (d, *J* = 8.3 Hz, 1H), 8.14 (s, 1H), 7.91 (s, 1H), 7.89–7.86 (m, 1H), 7.73 (dd, *J* = 8.0, 1.4 Hz, 1H), 7.64 (s, 1H), 7.56 (t, *J* = 7.8 Hz, 1H), 7.43 (d, *J* = 7.7 Hz, 1H), 7.33 (t, *J* = 7.9 Hz, 1H), 7.22 (t, *J* = 7.6 Hz, 1H), 6.49 (d, *J* = 5.0 Hz, 1H), 3.65 (s, 3H), 3.42–3.39 (m, 2H), 3.26–3.23 (m, 1H), 2.32 (t, *J* = 7.4 Hz, 2H), 1.67–1.64 (m, 2H), 1.62–1.56 (m, 2H), 1.41–1.37 (m, 2H), 1.30 (d, *J* = 6.9 Hz, 6H). MS (ESI) *m*/*z*: [M + H]^+^: 574.3.

##### Methyl 7–(3-((5-chloro-4-((2-(isopropylsulfonyl)phenyl)amino)pyrimidin-2-yl)amino)benzamido)heptanoate (9j)

Yellow solid. Yield: 59%. M.p. 128.1–129.4 °C. ^1^H NMR (600 MHz, CDCl_3_) δ 9.66 (s, 1H), 8.57 (t, *J* = 8.1 Hz, 1H), 8.15 (d, *J* = 6.3 Hz, 1H), 7.92–7.87 (m, 2H), 7.73 (dd, *J* = 8.1, 1.2 Hz, 1H), 7.59–7.57 (m, 1H), 7.51 (s, 1H), 7.41 (d, *J* = 7.8 Hz, 1H), 7.34 (t, *J* = 7.9 Hz, 1H), 7.23 (t, *J* = 7.6 Hz, 1H), 6.31 (t, *J* = 5.5 Hz, 1H), 3.66 (s, 3H), 3.41–3.38 (m, 2H), 3.27–3.21 (m, 1H), 2.31 (t, *J* = 7.5 Hz, 2H), 1.65–1.62 (m, 2H), 1.60–1.57 (m, 2H), 1.37 (dd, *J* = 9.7, 6.1 Hz, 4H), 1.31 (d, *J* = 6.9 Hz, 6H). MS (ESI) *m*/*z*: [M + H]^+^: 588.5.

#### General procedure for the synthesis of intermediates 10a–j

Synthesised using the preparation method of **5a** using **9a** (0.91 g, 1.70 mmol), NaOH (0.22 g, 5.50 mmol) in MeOH/H_2_O solution (80%, 10 mL), and 0.79 g of **10a** was gained as white solid. **10b–j** were synthesised in the manner of **10a**.

#### General procedure for the synthesis of 11a–j

##### N-(3-((2-aminophenyl)amino)-3-oxopropyl)-4-((5-chloro-4-((2-(isopropylsulfonyl)phenyl)amino)pyrimidin-2-yl)amino)benzamide (11a)

Compounds **11a–j** was synthesised according to the preparation procedure of **4a**, using **10a** (0.78 g, 1.50 mmol), *o*-phenylenediamine (0.17 g, 1.50 mmol), HOBT (0.40 g, 3.00 mmol), EDCI (0.58 g, 3.00 mmol) and DIPEA (0.77 g, 6.00 mmol) in 10 mL DMF, and 0.50 g of **11a** was gained as white solid. Yield: 55%. M.p. 215.2–217.7 °C. ^1^H NMR (600 MHz, DMSO-d_6_) δ 9.82 (s, 1H), 9.49 (s, 1H), 9.17 (s, 1H), 8.56 (d, *J* = 6.9 Hz, 1H), 8.43 (t, *J* = 5.5 Hz, 1H), 8.35 (s, 1H), 7.88 (dd, *J* = 7.9, 1.3 Hz, 1H), 7.83–7.79 (m, 1H), 7.75 (d, *J* = 8.7 Hz, 2H), 7.70 (d, *J* = 8.6 Hz, 2H), 7.43 (t, *J* = 7.6 Hz, 1H), 7.16 (d, *J* = 6.9 Hz, 1H), 6.92–6.88 (m, 1H), 6.71 (dd, *J* = 7.9, 0.9 Hz, 1H), 6.55–6.51 (m, 1H), 4.86 (s, 2H), 3.58–3.55 (m, 2H), 3.47–3.44 (m, 1H), 2.62 (t, *J* = 7.0 Hz, 2H), 1.16 (d, *J* = 6.8 Hz, 6H). ^13 ^C NMR (151 MHz, DMSO-d_6_) δ 170.05, 166.42, 157.72, 155.67, 155.56, 143.24, 142.65, 138.30, 135.39, 131.45, 128.24, 127.74, 126.38, 126.09, 125.61, 124.99, 124.56, 123.70, 118.47, 116.50, 116.23, 105.95, 55.30, 36.57, 36.46, 15.32. HRMS *m*/*z* calcd for C_29_H_31_ClN_7_O_4_S [M + H]^+^: 608.1847, found: 608.1845.

##### N-(4-((2-aminophenyl)amino)-4-oxobutyl)-4-((5-chloro-4-((2-(isopropylsulfonyl)phenyl)amino)pyrimidin-2-yl)amino)benzamide (11b)

White solid. Yield: 36%. M.p. 158.9–160.3 °C. ^1^H NMR (600 MHz, DMSO-d_6_) δ 9.82 (s, 1H), 9.49 (s, 1H), 9.13 (s, 1H), 8.56 (d, *J* = 7.2 Hz, 1H), 8.38–8.33 (m, 2H), 7.88 (dd, *J* = 8.0, 1.4 Hz, 1H), 7.84–7.80 (m, 1H), 7.75 (d, *J* = 8.8 Hz, 2H), 7.70 (d, *J* = 8.6 Hz, 2H), 7.44 (t, *J* = 7.6 Hz, 1H), 7.15 (dd, *J* = 7.8, 1.0 Hz, 1H), 6.91–6.87 (m, 1H), 6.72 (dd, *J* = 8.0, 1.1 Hz, 1H), 6.55–6.51 (m, 1H), 4.97 (s, 2H), 3.50–3.42 (m, 1H), 3.31 (d, *J* = 6.8 Hz, 2H).2.38 (t, *J* = 7.4 Hz, 2H), 1.89–1.83 (m, 2H), 1.16 (d, *J* = 6.8 Hz, 6H). ^13 ^C NMR (151 MHz, DMSO-d_6_) δ 171.40, 166.30, 157.73, 155.69, 155.57, 143.19, 142.43, 138.31, 135.41, 131.46, 128.25, 127.84, 126.23, 125.94, 125.60, 124.99, 124.58, 123.95, 118.43, 116.61, 116.30, 105.94, 55.28, 39.21, 33.77, 26.00, 15.32. HRMS *m*/*z* calcd for C_30_H_33_ClN_7_O_4_S [M + H]^+^: 622.2003, found: 622.2005.

##### N-(5-((2-aminophenyl)amino)-5-oxopentyl)-4-((5-chloro-4-((2-(isopropylsulfonyl)phenyl)amino)pyrimidin-2-yl)amino)benzamide (11c)

White solid. Yield: 52%. M.p. 184.3–186.2 °C. ^1^H NMR (600 MHz, DMSO-d_6_) δ 9.81 (s, 1H), 9.49 (s, 1H), 9.10 (s, 1H), 8.56 (d, *J* = 7.0 Hz, 1H), 8.36 (s, 1H), 8.31 (t, *J* = 5.6 Hz, 1H), 7.88 (dd, *J* = 8.0, 1.4 Hz, 1H), 7.84–7.80 (m, 1H), 7.74 (d, *J* = 8.7 Hz, 2H), 7.70 (d, *J* = 8.6 Hz, 2H), 7.44 (t, *J* = 7.6 Hz, 1H), 7.17 (d, *J* = 7.0 Hz, 1H), 6.91–6.87 (m, 1H), 6.73–6.70 (m, 1H), 6.55–6.51 (m, 1H), 4.83 (s, 2H), 3.48–3.44 (m, 1H), 3.31–3.28 (m, 2H), 2.36 (t, *J* = 7.3 Hz, 2H), 1.68–1.62 (m, 2H), 1.60–1.55 (m, 2H), 1.17 (d, *J* = 6.8 Hz, 6H). ^13 ^C NMR (151 MHz, DMSO-d_6_) δ 171.55, 166.15, 157.74, 155.68, 155.56, 143.14, 142.34, 138.31, 135.39, 131.45, 128.20, 127.93, 126.15, 125.75, 125.57, 124.96, 124.56, 124.02, 118.45, 116.61, 116.33, 105.93, 55.28, 35.98, 29.46, 23.41, 22.57, 15.32. HRMS *m*/*z* calcd for C_31_H_35_ClN_7_O_4_S [M + H]^+^: 636.2160, found: 636.2158.

##### N-(6-((2-aminophenyl)amino)-6-oxohexyl)-4-((5-chloro-4-((2-(isopropylsulfonyl)phenyl)amino)pyrimidin-2-yl)amino)benzamide (11d)

White solid. Yield: 59%. M.p. 198.2–199.1 °C. ^1^H NMR (600 MHz, DMSO-d_6_) δ 9.82 (s, 1H), 9.50 (s, 1H), 9.09 (s, 1H), 8.57 (d, *J* = 6.5 Hz, 1H), 8.36 (s, 1H), 8.29 (t, *J* = 5.5 Hz, 1H), 7.89 (dd, *J* = 7.9, 1.4 Hz, 1H), 7.84–7.80 (m, 1H), 7.75 (d, *J* = 8.7 Hz, 2H), 7.70 (d, *J* = 8.6 Hz, 2H), 7.44 (dd, *J* = 11.3, 4.0 Hz, 1H), 7.15 (dd, *J* = 7.8, 1.0 Hz, 1H), 6.91–6.87 (m, 1H), 6.72 (dd, *J* = 7.9, 1.1 Hz, 1H), 6.55–6.49 (m, 1H), 4.81 (s, 2H), 3.51–3.43 (m, 1H), 3.29–3.24 (m, 2H), 2.33 (t, *J* = 7.4 Hz, 2H), 1.67–1.61 (m, 2H), 1.59–1.55 (m, 2H), 1.40–1.36 (m, 2H), 1.17 (d, *J* = 6.8 Hz, 6H). ^13 ^C NMR (151 MHz, DMSO-d_6_) δ 171.60, 166.13, 157.74, 155.67, 155.55, 143.12, 142.36, 138.32, 135.38, 131.45, 128.19, 127.96, 126.15, 125.77, 125.55, 124.93, 124.53, 124.04, 118.45, 116.63, 116.35, 105.94, 55.29, 40.53, 36.23, 29.61, 26.72, 25.61, 15.32. HRMS *m*/*z* calcd for C_32_H_37_ClN_7_O_4_S [M + H]^+^: 650.2316, found: 650.2307.

##### N-(7-((2-aminophenyl)amino)-7-oxoheptyl)-4-((5-chloro-4-((2-(isopropylsulfonyl)phenyl)amino)pyrimidin-2-yl)amino)benzamide (11e)

White solid. Yield: 42%. M.p. 163.2–165.6 °C. ^1^H NMR (600 MHz, DMSO-d_6_) δ 9.81 (s, 1H), 9.49 (s, 1H), 9.09 (s, 1H), 8.57 (d, *J* = 6.9 Hz, 1H), 8.36 (s, 1H), 8.26 (t, *J* = 5.5 Hz, 1H), 7.88 (dd, *J* = 7.9, 1.2 Hz, 1H), 7.84–7.80 (m, 1H), 7.73 (d, *J* = 8.7 Hz, 2H), 7.69 (d, *J* = 8.7 Hz, 2H), 7.44 (t, *J* = 7.6 Hz, 1H), 7.15 (d, *J* = 7.2 Hz, 1H), 6.88 (t, *J* = 7.1 Hz, 1H), 6.71 (d, *J* = 7.2 Hz, 1H), 6.53 (t, *J* = 7.1 Hz, 1H), 4.81 (s, 2H), 3.51–3.43 (m, 1H), 3.26–3.22 (m, 2H), 2.32 (t, *J* = 7.4 Hz, 2H), 1.64–1.58 (m, 2H), 1.56–1.51 (m, 2H), 1.35 (d, *J* = 8.3 Hz, 4H), 1.17 (d, *J* = 6.8 Hz, 6H). ^13 ^C NMR (151 MHz, DMSO-d_6_) δ 171.62, 166.12, 157.74, 156.28, 155.68, 155.56, 143.11, 142.35, 138.32, 135.38, 131.46, 128.18, 127.97, 126.15, 125.74, 125.58, 124.95, 124.55, 124.07, 118.45, 116.65, 116.37, 105.93, 55.29, 36.23, 29.68, 28.96, 26.82, 25.78, 15.32. HRMS *m*/*z* calcd for C_33_H_39_ClN_7_O_4_S [M + H]^+^: 664.2473, found: 664.2476.

##### N-(3-((2-aminophenyl)amino)-3-oxopropyl)-3-((5-chloro-4-((2-(isopropylsulfonyl)phenyl)amino)pyrimidin-2-yl)amino)benzamide (11f)

White solid. Yield: 76%. M.p. 143.2–145.0 °C. ^1^H NMR (600 MHz, DMSO-d_6_) δ 9.69 (s, 1H), 9.56 (s, 1H), 9.17 (s, 1H), 8.66 (s, 1H), 8.49 (t, *J* = 5.5 Hz, 1H), 8.32 (s, 1H), 8.05 (s, 1H), 7.84 (dd, *J* = 8.0, 1.5 Hz, 1H), 7.79 (d, *J* = 7.7 Hz, 1H), 7.70 (t, *J* = 7.6 Hz, 1H), 7.45 (d, *J* = 7.7 Hz, 1H), 7.37–7.33 (m, 2H), 7.16 (dd, *J* = 7.8, 1.2 Hz, 1H), 6.91–6.87 (m, 1H), 6.71 (dd, *J* = 8.0, 1.2 Hz, 1H), 6.55–6.51 (m, 1H), 4.86 (s, 2H), 3.58–3.54 (m, 2H), 3.47–3.43 (m, 1H), 2.62 (t, *J* = 7.0 Hz, 2H), 1.17 (d, *J* = 6.8 Hz, 6H). ^13 ^C NMR (151 MHz, DMSO-d_6_) δ 169.95, 166.99, 158.09, 155.69, 155.31, 142.62, 140.56, 138.42, 135.67, 135.43, 131.39, 128.77, 126.36, 126.08, 124.60, 124.09, 124.02, 123.70, 122.78, 120.92, 119.60, 116.50, 116.22, 105.65, 55.37, 36.65, 36.24, 15.34. HRMS *m*/*z* calcd for C_29_H_31_ClN_7_O_4_S [M + H]^+^: 608.1847, found: 608.1848.

##### N-(4-((2-aminophenyl)amino)-4-oxobutyl)-3-((5-chloro-4-((2-(isopropylsulfonyl)phenyl)amino)pyrimidin-2-yl)amino)benzamide (11g)

White solid. Yield: 76%. M.p. 179.3–182.1 °C. ^1^H NMR (600 MHz, DMSO-d_6_) δ 9.69 (s, 1H), 9.58 (s, 1H), 9.12 (s, 1H), 8.66 (s, 1H), 8.43 (t, *J* = 5.5 Hz, 1H), 8.33 (s, 1H), 8.07 (s, 1H), 7.83 (dd, *J* = 7.9, 1.2 Hz, 1H), 7.78 (d, *J* = 7.3 Hz, 1H), 7.68 (t, *J* = 7.8 Hz, 1H), 7.46 (d, *J* = 7.7 Hz, 1H), 7.36 (d, *J* = 8.1 Hz, 1H), 7.34 (d, *J* = 8.1 Hz, 1H), 7.16 (d, *J* = 7.2 Hz, 1H), 6.91–6.87 (m, 1H), 6.72 (d, *J* = 7.2 Hz, 1H), 6.53 (t, *J* = 7.2 Hz, 1H), 4.86 (s, 2H), 3.46–3.44 (m, 1H), 3.34–3.31 (m, 2H), 2.38 (t, *J* = 7.4 Hz, 2H), 1.84 (p, *J* = 7.2 Hz, 2H), 1.17 (d, *J* = 6.8 Hz, 6H). ^13 ^C NMR (151 MHz, DMSO-d_6_) δ 171.34, 166.95, 158.11, 155.73, 155.29, 142.48, 140.52, 138.44, 135.80, 135.41, 131.40, 128.74, 126.23, 125.90, 124.55, 124.01, 123.97, 123.92, 122.76, 120.98, 119.69, 116.57, 116.28, 105.62, 55.39, 39.38, 33.79, 25.87, 15.33. HRMS *m*/*z* calcd for C_30_H_33_ClN_7_O_4_S [M + H]^+^: 622.2003, found: 622.2006.

##### N-(5-((2-aminophenyl)amino)-5-oxopentyl)-3-((5-chloro-4-((2-(isopropylsulfonyl)phenyl)amino)pyrimidin-2-yl)amino)benzamide (11h)

Yellow solid. Yield: 90%. M.p. 165.8–169.3 °C. ^1^H NMR (600 MHz, DMSO-d_6_) δ 9.68 (s, 1H), 9.57 (s, 1H), 9.09 (s, 1H), 8.66 (s, 1H), 8.40 (t, *J* = 5.6 Hz, 1H), 8.32 (s, 1H), 8.04 (s, 1H), 7.83 (dd, *J* = 8.0, 1.5 Hz, 1H), 7.76 (d, *J* = 7.3 Hz, 1H), 7.68 (t, *J* = 7.8 Hz, 1H), 7.44 (d, *J* = 7.7 Hz, 1H), 7.37–7.34 (m, 1H), 7.33 (dd, *J* = 8.7, 4.2 Hz, 1H), 7.16 (dd, *J* = 7.8, 1.2 Hz, 1H), 6.90–6.86 (m, 1H), 6.71 (dd, *J* = 8.0, 1.2 Hz, 1H), 6.55–6.54 (m, 1H), 4.82 (s, 2H), 3.49–3.41 (m, 1H), 3.27–3.24 (m, 2H), 2.35 (t, *J* = 7.3 Hz, 2H), 1.68–1.61 (m, 2H), 1.58–1.54 (m, 2H), 1.17 (d, *J* = 6.8 Hz, 6H). ^13 ^C NMR (151 MHz, DMSO-d_6_) δ 171.52, 166.81, 158.11, 155.73, 155.29, 142.32, 140.49, 138.46, 138.43, 135.87, 135.40, 131.39, 128.74, 126.15, 125.73, 124.55, 124.01, 123.97, 122.72, 120.95, 119.68, 116.62, 116.33, 105.60, 55.37, 39.53, 35.94, 29.29, 23.37, 15.33. HRMS *m*/*z* calcd for C_31_H_35_ClN_7_O_4_S [M + H]^+^: 636.2160, found: 636.2161.

##### N-(6-((2-aminophenyl)amino)-6-oxohexyl)-3-((5-chloro-4-((2-(isopropylsulfonyl)phenyl)amino)pyrimidin-2-yl)amino)benzamide (11i)

Yellow solid. Yield: 68%. M.p. 132.4–135.5 °C. ^1^H NMR (600 MHz, DMSO-d_6_) δ 9.68 (s, 1H), 9.57 (s, 1H), 9.08 (s, 1H), 8.66 (s, 1H), 8.36 (t, *J* = 5.5 Hz, 1H), 8.32 (s, 1H), 8.03 (s, 1H), 7.84 (dd, *J* = 7.9, 1.5 Hz, 1H), 7.76 (d, *J* = 7.2 Hz, 1H), 7.68 (t, *J* = 7.6 Hz, 1H), 7.43 (d, *J* = 7.7 Hz, 1H), 7.35 (t, *J* = 7.7 Hz, 1H), 7.32 (t, *J* = 7.9 Hz, 1H), 7.16–7.13 (m, 1H), 6.90–6.86 (m, 1H), 6.72–6.69 (m, 1H), 6.54–6.51 (m, 1H), 4.81 (s, 2H), 3.47–3.43 (m, 1H), 3.27–3.24 (m, 2H), 2.32 (t, *J* = 7.4 Hz, 2H), 1.65–1.59 (m, 2H), 1.56–1.53 (m, 2H), 1.38–1.34 (m, 2H), 1.17 (d, *J* = 6.8 Hz, 6H). ^13 ^C NMR (151 MHz, DMSO-d_6_) δ 171.58, 166.78, 158.11, 155.73, 155.29, 142.35, 140.49, 138.44, 135.90, 135.40, 131.40, 128.73, 126.14, 125.76, 124.54, 124.05, 124.01, 123.96, 122.70, 120.94, 119.67, 116.64, 116.35,105.60, 55.37, 36.22, 31.76, 29.43, 26.69, 25.58, 15.34. HRMS *m*/*z* calcd for C_32_H_37_ClN_7_O_4_S [M + H]^+^: 650.2316, found: 650.2314.

##### N-(7-((2-aminophenyl)amino)-7-oxoheptyl)-3-((5-chloro-4-((2-(isopropylsulfonyl)phenyl)amino)pyrimidin-2-yl)amino)benzamide (11j)

Yellow solid. Yield: 84%. M.p. 101.5–103.4 °C. ^1^H NMR (600 MHz, DMSO-d_6_) δ 9.68 (s, 1H), 9.57 (s, 1H), 9.10 (s, 1H), 8.66 (d, *J* = 6.1 Hz, 1H), 8.35 (t, *J* = 5.5 Hz, 1H), 8.32 (s, 1H), 8.03 (s, 1H), 7.85–7.82 (m, 1H), 7.76 (d, *J* = 7.4 Hz, 1H), 7.67 (t, *J* = 7.5 Hz, 1H), 7.43 (d, *J* = 7.7 Hz, 1H), 7.35 (dd, *J* = 8.9, 4.9 Hz, 1H), 7.33 (t, *J* = 6.0 Hz, 1H), 7.17–7.14 (m, 1H), 6.91–6.86 (m, 1H), 6.72 (dd, *J* = 7.9, 1.0 Hz, 1H), 6.55–6.51 (m, 1H), 4.83 (s, 2H), 3.46–3.43 (m, 1H), 3.26–3.24 (m, 2H), 2.32 (t, *J* = 7.4 Hz, 2H), 1.63–1.57 (m, 2H), 1.53–1.49 (m, 2H), 1.34 (s, 4H), 1.17 (d, *J* = 6.8 Hz, 6H). ^13 ^C NMR (151 MHz, DMSO-d_6_) δ 171.64, 166.80, 158.12, 155.72, 155.28, 142.33, 140.47, 138.44, 135.91, 135.39, 131.40, 128.74, 126.16, 125.74, 124.50, 124.07, 123.98, 123.93, 122.72, 120.97, 119.68, 116.68, 116.39, 105.60, 55.39, 39.56, 36.23, 29.51, 28.94, 26.81, 25.77, 15.33. HRMS *m*/*z* calcd for C_33_H_39_ClN_7_O_4_S [M + H]^+^: 664.2473, found: 664.2464.

#### General procedure for the synthesis of 12a–h

##### N-(2-aminophenyl)-4-((5-chloro-4-((2-(isopropylsulfonyl)phenyl)amino)pyrimidin-2-yl)amino)benzamide (12a)

Compounds **12a-j** was synthesised according to the preparation procedure of **4a**, using **8a** (1.00 g, 2.24 mmol), o-phenylenediamine (0.25 g, 2.12 mmol), HOBT (0.60 g, 4.40 mmol), EDCI (0.86 g, 4.49 mmol) and DIPEA (1.19 g, 9.20 mmol) in 10 mL DMF, and 0.22 g of **12a** was gained as white solid. Yield: 30%. M.p. 207.6–209.3 °C. ^1^H NMR (600 MHz, DMSO-d_6_) δ 9.87 (s, 1H), 9.50 (s, 2H), 8.58 (d, *J* = 7.9 Hz, 1H), 8.37 (s, 1H), 7.90–7.84 (m, 4H), 7.76 (d, *J* = 8.6 Hz, 2H), 7.45 (t, *J* = 7.5 Hz, 1H), 7.16 (d, *J* = 7.3 Hz, 1H), 6.97 (t, *J* = 7.6 Hz, 1H), 6.79 (d, *J* = 8.1 Hz, 1H), 6.61 (t, *J* = 7.5 Hz, 1H), 4.86 (s, 2H), 3.49– 3.45 (m, 1H), 1.18 (d, *J* = 6.8 Hz, 6H). ^13 ^C NMR (151 MHz, DMSO-d_6_) δ 165.25, 157.71, 155.69, 155.61, 143.64, 143.56, 138.30, 135.45, 131.48, 128.91, 127.63, 127.15, 126.81, 125.04, 124.61, 124.09, 118.38, 116.77, 116.63, 109.49, 106.05, 55.29, 15.33. HRMS *m*/*z* calcd for C_26_H_26_ClN_6_O_3_S [M + H]^+^: 537.1476, found: 537.1479.

##### N-(2-amino-4,5-dichlorophenyl)-4-((5-chloro-4-((2-(isopropylsulfonyl)phenyl)amino)pyrimidin-2-yl)amino)benzamide (12b)

Pink solid. Yield: 31%. M.p. 218.4–220.5 °C. ^1^H NMR (600 MHz, DMSO-d_6_) δ 9.87 (s, 1H), 9.55 (s, 1H), 9.52 (s, 1H), 8.60 (d, *J* = 7.8 Hz, 1H), 8.33 (s, 1H), 7.91 (s, 1H), 7.90 (s, 1H), 7.87 (dd, *J* = 8.0, 1.2 Hz, 1H), 7.85–7.78 (m, 1H), 7.80 (s, 1H), 7.78 (s, 1H), 7.46 (s, 1H), 7.42 (t, *J* = 7.6 Hz, 1H), 7.00 (s, 1H), 5.38 (s, 2H), 3.44–3.41 (m, 1H), 1.17 (d, *J* = 6.8 Hz, 6H). ^13 ^C NMR (151 MHz, DMSO-d_6_) δ 165.62, 157.68, 155.66, 155.61, 143.97, 143.84, 138.31, 135.44, 131.48, 129.08, 128.23, 128.07, 127.14, 125.64, 125.00, 124.60, 123.96, 118.35, 116.59, 116.48, 106.15, 55.30, 15.33. HRMS *m*/*z* calcd for C_26_H_24_Cl_3_N_6_O_3_S [M + H]^+^: 605.0696, found: 605.0695.

##### N-(2-amino-4,5-dibromophenyl)-4-((5-chloro-4-((2-(isopropylsulfonyl)phenyl)amino)pyrimidin-2-yl)amino)benzamide (12c)

Yellow solid. Yield: 27%. M.p. 218.6–219.3 °C. ^1^H NMR (600 MHz, DMSO-d_6_) δ 9.88 (s, 1H), 9.50 (s, 2H), 8.58 (d, *J* = 8.0 Hz, 1H), 8.37 (s, 1H), 7.91–7.87 (m, 2H), 7.87–7.84 (m, 2H), 7.77 (s, 1H), 7.76 (s, 1H), 7.55 (s, 1H), 7.45 (t, *J* = 7.6 Hz, 1H), 7.15 (s, 1H), 5.38 (s, 2H), 3.50–3.44 (m, 1H), 1.18 (d, *J* = 6.8 Hz, 6H). ^13 ^C NMR (151 MHz, DMSO-d_6_) δ 165.59, 157.67, 155.67, 155.62, 144.56, 143.83, 138.29, 135.45, 131.48, 131.00, 129.07, 127.12, 125.68, 125.07, 124.70, 124.64, 120.67, 119.71, 118.34, 107.77, 106.13, 55.30, 15.33. HRMS *m*/*z* calcd for C_26_H_24_Br_2_ClN_6_O_3_S [M + H]^+^: 692.9686, found: 692.9684.

##### N-(2-amino-4,5-dimethylphenyl)-4-((5-chloro-4-((2-(isopropylsulfonyl)phenyl)amino)pyrimidin-2-yl)amino)benzamide (12d)

Brown solid. Yield: 44%. M.p. 192.6–194.5 °C. ^1^H NMR (600 MHz, DMSO-d_6_) δ 9.88 (s, 1H), 9.50 (s, 1H), 9.45 (s, 1H), 8.58 (d, *J* = 7.0 Hz, 1H), 8.37 (s, 1H), 7.92–7.88 (m, 2H), 7.87–7.83 (m, 2H), 7.76 (s, 1H), 7.75 (s, 1H), 7.45 (t, *J* = 7.4 Hz, 1H), 6.92 (s, 1H), 6.59 (s, 1H), 4.59 (s, 2H), 3.49–3.45 (m, 1H), 2.12 (s, 3H), 2.09 (s, 3H)., 1.17 (d, *J* = 6.8 Hz, 6H). ^13 ^C NMR (151 MHz, DMSO-d_6_) δ 165.08, 157.72, 155.68, 155.60, 143.47, 141.16, 138.31, 135.44, 134.28, 131.47, 128.83, 127.97, 127.71, 125.64, 125.03, 124.60, 124.13, 121.83, 118.39, 118.15, 106.02, 55.29, 19.61, 18.88, 15.33. HRMS *m*/*z* calcd for C_28_H_30_ClN_6_O_3_S [M + H]^+^: 565.1789, found: 565.1791.

##### N-(2-amino-4-fluorophenyl)-4-((5-chloro-4-((2-(isopropylsulfonyl)phenyl)amino)pyrimidin-2-yl)amino)benzamide (12e)

Yellow solid. Yield: 29%. M.p. 196.7–198.3 °C. ^1^H NMR (600 MHz, DMSO-d_6_) δ 9.88 (d, *J* = 6.9 Hz, 1H), 9.50 (s, 1H), 9.45 (s, 1H), 8.58 (s, 1H), 8.42–8.33 (m, 1H), 7.95–7.84 (m, 4H), 7.76 (d, *J* = 7.1 Hz, 2H), 7.49–7.42 (m, 1H), 7.11 (d, *J* = 6.5 Hz, 1H), 6.60–6.51 (m, 1H), 6.41–6.32 (m, 1H), 5.20 (s, 2H), 3.53–3.43 (m, 1H), 1.17 (d, *J* = 6.7 Hz, 6H). ^13 ^C NMR (151 MHz, DMSO-d_6_) δ 165.52, 162.22, 160.64, 157.71, 155.68, 155.61, 146.02, 145.94, 143.57, 138.30, 135.45, 131.47, 129.05, 128.98, 128.92, 127.51, 125.02, 124.61, 120.02, 118.35, 106.05, 55.29, 15.33. HRMS *m*/*z* calcd for C_26_H_25_ClFN_6_O_3_S [M + H]^+^: 555.1381, found: 555.1381.

##### N-(2-amino-4-chlorophenyl)-4-((5-chloro-4-((2-(isopropylsulfonyl)phenyl)amino)pyrimidin-2-yl)amino)benzamide (12f)

White solid. Yield: 36%. M.p. 193.5–196.8 °C. ^1^H NMR (600 MHz, DMSO-d_6_) δ 9.88 (s, 1H), 9.50 (s, 1H), 9.48 (s, 1H), 8.58 (d, *J* = 7.2 Hz, 1H), 8.37 (s, 1H), 7.89 (d, *J* = 8.0 Hz, 2H), 7.87–7.84 (m, 2H), 7.77 (s, 1H), 7.75 (s, 1H), 7.45 (t, *J* = 7.6 Hz, 1H), 7.16 (d, *J* = 8.4 Hz, 1H), 6.82 (d, *J* = 2.2 Hz, 1H), 6.59 (dd, *J* = 8.3, 2.2 Hz, 1H), 5.21 (s, 2H), 3.49–3.47 (m, 1H), 1.17 (d, *J* = 6.8 Hz, 6H). ^13 ^C NMR (151 MHz, DMSO-d_6_) δ 165.52, 162.22, 160.64, 157.71, 155.68, 155.61, 146.02, 145.94, 143.57, 138.30, 135.45, 131,47, 129.05, 128.98, 128.92, 127.51, 125.03, 124.61, 120.02, 118.35, 106.05, 55.29, 15.33. HRMS *m*/*z* calcd for C_26_H_25_Cl_2_N_6_O_3_S [M + H]^+^: 571.1086, found: 571.1084.

##### N-(2-amino-4-methoxyphenyl)-4-((5-chloro-4-((2-(isopropylsulfonyl)phenyl)amino)pyrimidin-2-yl)amino)benzamide (12g)

Brown solid. Yield: 29%. M.p. 205.6–207.7 °C. ^1^H NMR (600 MHz, DMSO-d_6_) δ 9.87 (s, 1H), 9.50 (s, 1H), 9.39 (s, 1H), 8.58 (d, *J* = 7.0 Hz, 1H), 8.37 (s, 1H), 7.89 (d, *J* = 6.9 Hz, 2H), 7.88–7.84 (m, 2H), 7.76 (s, 1H), 7.74 (s, 1H), 7.45 (t, *J* = 7.6 Hz, 1H), 7.00 (d, *J* = 8.5 Hz, 1H), 6.37 (d, *J* = 2.7 Hz, 1H), 6.19 (dd, *J* = 8.5, 2.5 Hz, 1H), 4.89 (s, 2H), 3.69 (s, 3H), 3.51–3.43 (m, 1H), 1.17 (d, *J* = 6.8 Hz, 6H). ^13 ^C NMR (151 MHz, DMSO-d_6_) δ 164.29, 157.52, 156.64, 154.61, 154.52, 144.07, 142.36, 137.23, 134.37, 130.39, 127.75, 127.32, 126.65, 124.58, 123.93, 123.51, 117.28, 116.24, 104.93, 101.33, 100.20, 54.24, 54.21, 14.25. HRMS *m*/*z* calcd for C_27_H_28_ClN_6_O_4_S [M + H]^+^: 567.1581, found: 567.1579.

##### N-(2-amino-4-methylphenyl)-4-((5-chloro-4-((2-(isopropylsulfonyl)phenyl)amino)pyrimidin-2-yl)amino)benzamide (12h)

White solid. Yield: 25%. M.p. 202.7–204.1 °C. ^1^H NMR (600 MHz, DMSO-d_6_) δ 9.88 (d, *J* = 4.7 Hz, 1H), 9.50 (s, 1H), 9.45 (s, 1H), 8.58 (d, *J* = 7.3 Hz, 1H), 8.37 (s, 1H), 7.89 (dd, *J* = 7.8, 1.3 Hz, 2H), 7.86 (d, *J* = 8.7 Hz, 2H), 7.76 (s, 1H), 7.75 (s, 1H), 7.45 (t, *J* = 7.6 Hz, 1H), 7.02 (d, *J* = 7.9 Hz, 1H), 6.60 (d, *J* = 1.1 Hz, 1H), 6.42 (d, *J* = 6.9 Hz, 1H), 4.79 (s, 2H), 3.51–3.43 (m, 1H), 2.19 (d, *J* = 9.4 Hz, 3H), 1.17 (d, *J* = 6.8 Hz, 6H). ^13 ^C NMR (151 MHz, DMSO-d_6_) δ 165.23, 157.72, 155.69, 155.61, 143.48, 143.45, 138.30, 135.82, 135.45, 131.47, 128.86, 127.69, 127.08, 124.61, 121.66, 118.37, 117.62, 117.04, 106.03, 56.50, 55.29, 21.30, 19.03, 15.33. HRMS *m*/*z* calcd for C_27_H_28_ClN_6_O_3_S [M + H]^+^: 551.1632, found: 551.1633.

### Cell culture

Human cancer cells A549, HepG2, MDA-MB-231, H2228 and SK-N-BE(2) were purchased from Chinese Cell bank of Sciences Academy (Shanghai, China). The tumour cells were cultured with 10% foetal bovine serum (FBS) (Gibco, US) DMEM or RPMI-1640 growth medium supplemented with 1% streptomycin and 1% penicillin in incubator at 37 °C with 5% CO_2_.

### Cell counting kit-8 (CCK-8) assay

Cell counting kit-8 (CCK-8) assay was employed to evaluate antiproliferative effects of target compounds. Briefly, cells were seeded in 96-well plates (4 × 10^3^ cells/well) and cultured overnight, then treated with compounds or positive control (Ceritinib, Entinostat) for 72 h. After that, the medium was removed and 10 μL freshly prepared CCK-8 solution was added. After incubation at 37 °C for 2 h, the absorbance (OD_450 nm_) was determined through a microplate reader (BioTek, US). The IC_50_ values of different compounds were calculated by SPSS 17.0 software.

### Enzyme inhibitory activity assay

The enzyme inhibitory activity assay was conducted with a fluorescent assay kit as we previously reported^23^.

### Migration assay

For Transwell method, the tumour cells were cultured in serum-free DMEM/RPMI-1640 medium for 24 h, then seeded into the upper chamber at a density of 3 × 10^5^ cells (100 μL/well), and DMEM/RPMI-1640 medium (750 μL/well) with 10% FBS was added to the lower chamber of 24-well plate. The cells of upper chamber were treated with the drug containing medium or DMSO control (100 μL/well) for 24 h at 37 °C. Finally, the migrated cells were fixed with 4% formaldehyde, dyed with crystal violet, and washed by PBS. The selected area was photographed by inverted microscope (Nikon, Japan) and determined using ImageJ software.

For wound healing method, A549, SK-N-BE(2) and H2228 cells were seeded in 6-well flat-bottomed plates and incubated overnight. Cells were scratched with a pipette tip to generate a clean-wound area in the cell layer, following washed with PBS and treated with compound **12a**, positive control (Ceritinib, Entinostat), then replenished with serium-free DMEM/RPMI-1640 medium. The cells were photographed by inverted microscope at 0 h, 24 h or 72 h. Quantification of the area of scratch were measured by ImageJ software.

### Flow cytometric analysis

Flow cytometric (FCM) was performed to detect cell cycle and apoptosis. Briefly, cancer cells (5 × 10^5^ cells/well) were grown in 6-well plate overnight and then incubated with compound **12a**, positive control (Ceritinib, Entinostat) for 24 h, then washed with PBS. In terms of cell cycle detection, resuspended with ice-cold ethanol (70%) for 24 h and stained with propidium iodine (PI) and Annexin V-FITC for 30 min. Finally, the samples were detected by FCM (CytoFLEX, Beckman Coulter, US). For apoptosis analysis, the cells were gathered with cold PBS, and then stained with PI and RNase A. Subsequently, the treated samples were tested with FCM.

### Western blot analysis

The cancer cells were seeded and treated with compound **12a**, positive control (Ceritinib, Entinostat) for 24 h, cells in each well were washed and decomposed in lysis buffer. Proteins were detached by SDS-PAGE and transferred to PVDF membrance. After being blocked by 5% BSA, washed and incubated with primary antibodies (1:1000 diluted) (Changzhou affinity Biosciences, China) at 4 °C, followed by incubated with horseradish peroxidase (HRP) conjugated secondary antibodies (1:5000 diluted) (Shanghai Beyotime institute of biotechnology, China). Finally, the enhanced chemiluminescence reagent was conducted to detect the bands. Immunoblotting was analysed by densitometry using the software ImageJ.

### Hoechst 33258 and AO/EB staining analysis

The tumour cells were stained with AO/EB or Hoechst 33258 to preliminarily identify apoptotic morphological changes. In short, H2228 or A549 cells were seeded into 6-well plates and treated with compound **12a**, positive control (Ceritinib, Entinostat) for 24 h. For AO/EB staining assay, after washing with PBS, cells were gathered and dyed with AO/EB for 15 min. Then cells were photographed using an inverted fluorescence microscope. For Hoechst 33258 staining assay, the cells were fixed with 4% polyformaldehyde for 10 min and stained with Hoechst 33258 solution after washing cells with PBS for twice. Finally, an anti-fluorescence quencher was added in each well for observation by an inverted fluorescence microscope.

### SK-N-BE(2) xenograft model assay

All animal studies were conducted according to the guidelines of the Animal Experimental Ethics Committee of Chongqing Medical University (ID. SCXK2018-0003). Female BALB/C nude mice aged 4 weeks, were utilised to establish the SK-N-BE(2) xenograft model for determining the antitumor effect of compound **12a** *in vivo*. To put it briefly, a 100 μL suspension of 5 × 10^6^ SK-N-BE(2) cells was injected subcutaneously into the one flank region of nude mouse (Chengdu Yaokang Bio-Technology Co., LTD, Chengdu, China). Once the size of the tumours reached approximately 100–150 mm^3^, the mice were randomly divided into four groups and intraperitoneally (i.p.) administrated with 0.9% NaCl, compound **12a** (25 and 100 mg/kg) or Ceritinib (100 mg/kg) once every two days for 16 days. The tumour volume was calculated with the formula (length × width^2^)/2. The tumour volume and body weight of each mouse were determined using calliper every 2 days. On day 16, the mice were sacrificed, xenograft tumour were dissected and the weight was measured in each group. The organs were further examined by H&E staining to observe drug toxicity.

### Molecular docking

The Molecular docking studies were conducted with AMDOCK software as previously reported^25^. (PDB Code: 4MKC) and (PDB Code: 5IWG) were used and obtained from the Protein Data Bank.

### Statistical analysis

The data were analysed using GraphPad Prism 6 software and expressed as mean ± SD of three independent experiments. The statistical analyses were performed using *t*-test and the statistical differences were measured using a one-way ANOVA with *p* < 0.05, which was considered as statistically significant.

## Supplementary Material

Supplemental Material
