# Supplementary material for: Design, synthesis and biological evaluation of 2,4-pyrimidinediamine derivatives as ALK and HDACs dual inhibitors for the treatment of ALK addicted cancer
Source: J Enzyme Inhib Med Chem. 2022 Sep 13;37(1):2512–29. doi: 10.1080/14756366.2022.2121822 (PMC9481106; doi:10.1080/14756366.2022.2121822)
Supplement: Supplemental Material [file IENZ_A_2121822_SM0772.pdf]

Supplementary Data

Design, Synthesis and Biological evaluation of 2,4-pyrimidinediamine derivatives as ALK/HDACs dual inhibitors for the treatment of ALK addicted cancer

Dafeng Guo [a, 1], Yu Yu [a, b, 1], Binyu Long [a], Ping Deng [a, b], Dongzhi Ran[a], Lei Han[a], Jiecheng Zheng [a, b], Zongjie Gan [a, b,*]

[a] *Department of Medicinal Chemistry, College of Pharmacy, Chongqing Medical University, Chongqing 400016, PR China*

[b] *Chongqing Research Center for Pharmaceutical Engineering, Chongqing Medical University, Chongqing 400016, PR China*

*Corresponding author: ZongJie Gan

[1] These authors contributed equally to this work.

Email: gzj@cqmu.edu.cn (Z. Gan)

Tel.: +86 23-68485161

^{1}H NMR and ^{13}C NMR spectra of target compounds

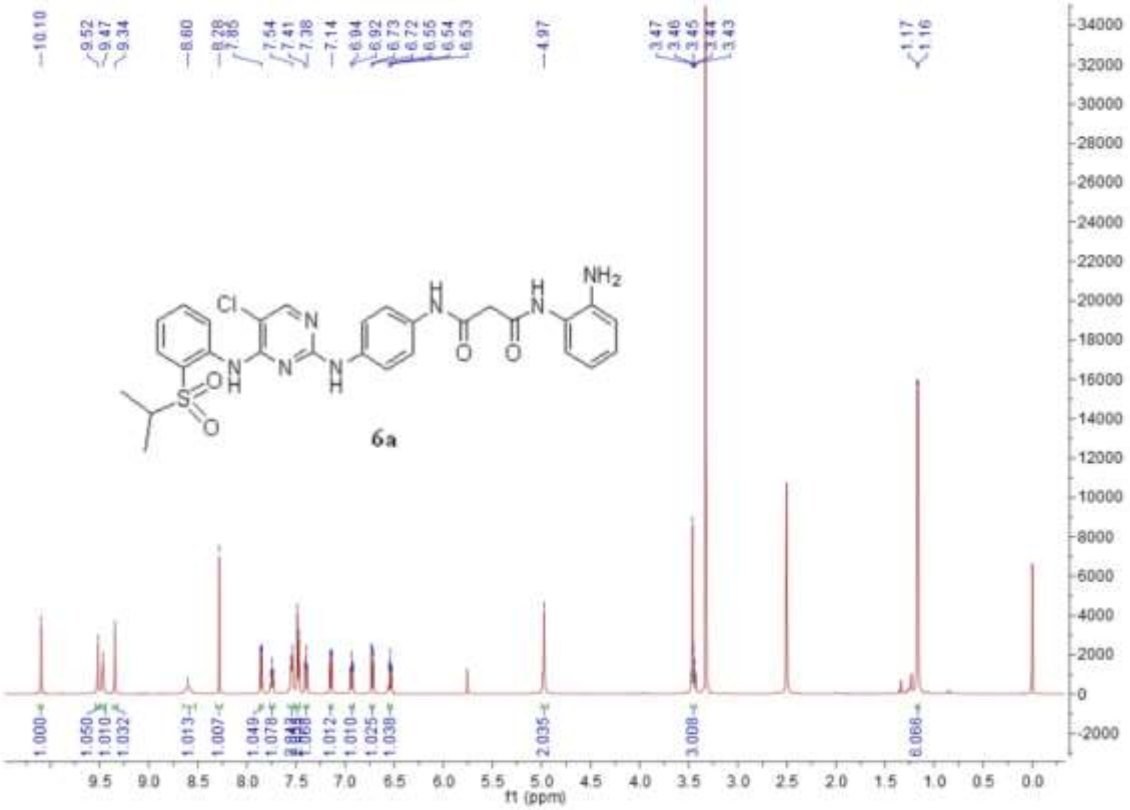

Figure 1. ^{1}H-NMR spectrum of **6a**

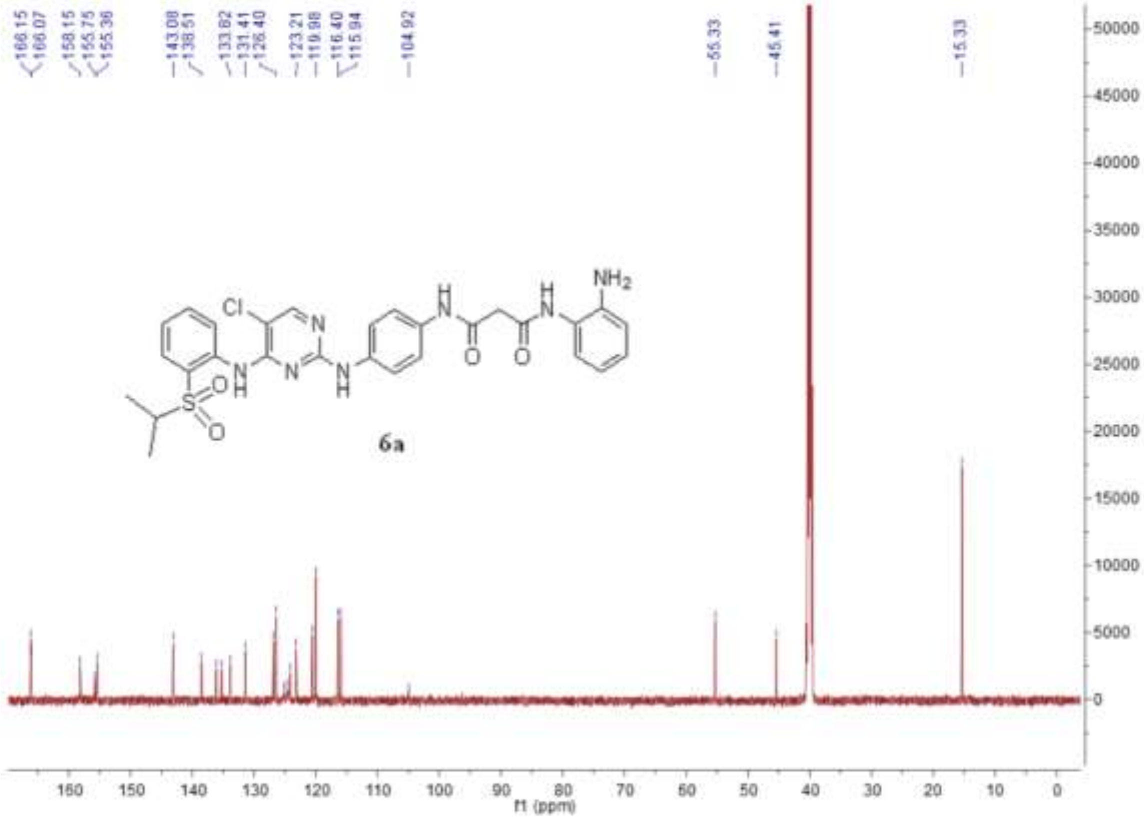

Figure 2. ^{13}C-NMR spectrum of **6a**

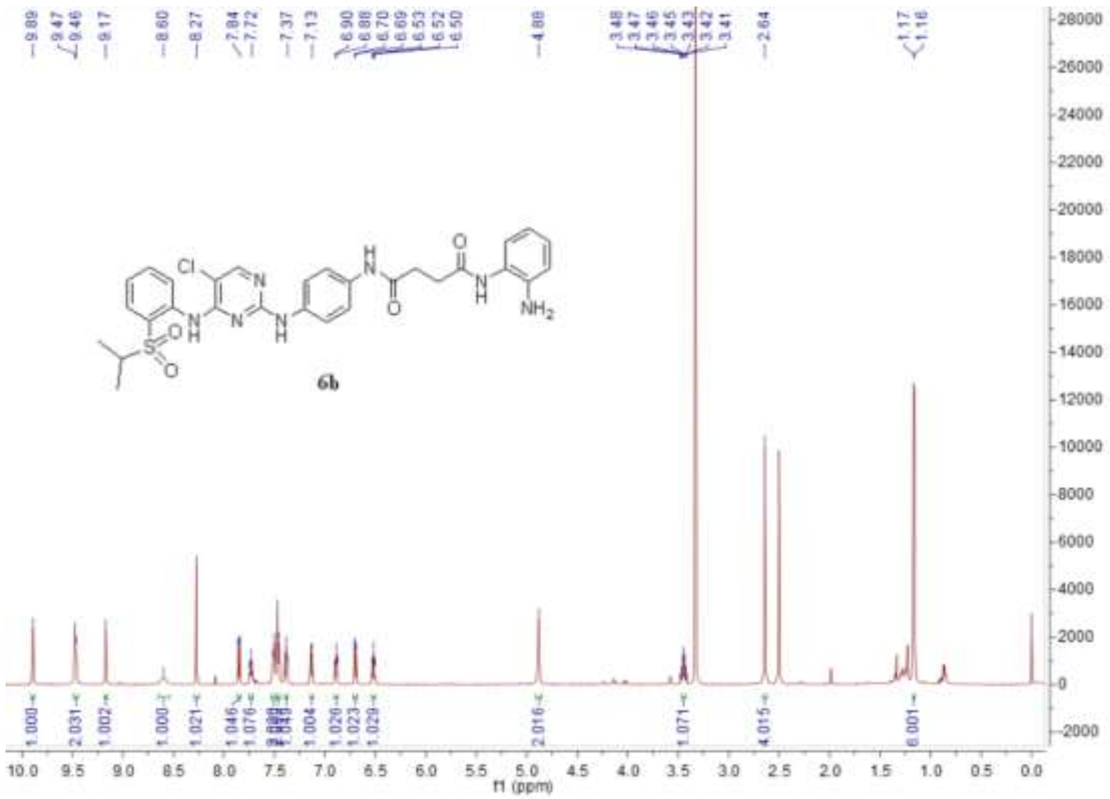

Figure 3. ^{1}H-NMR spectrum of **6b**

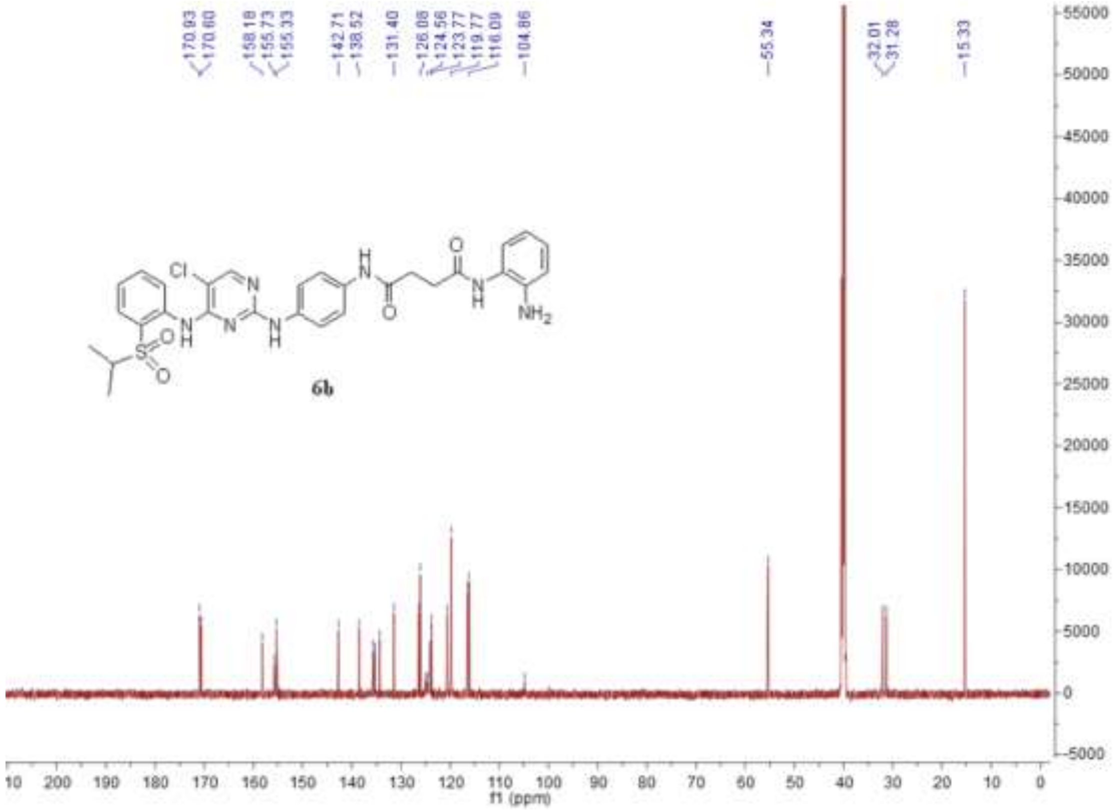

Figure 4. ^{13}C-NMR spectrum of **6b**

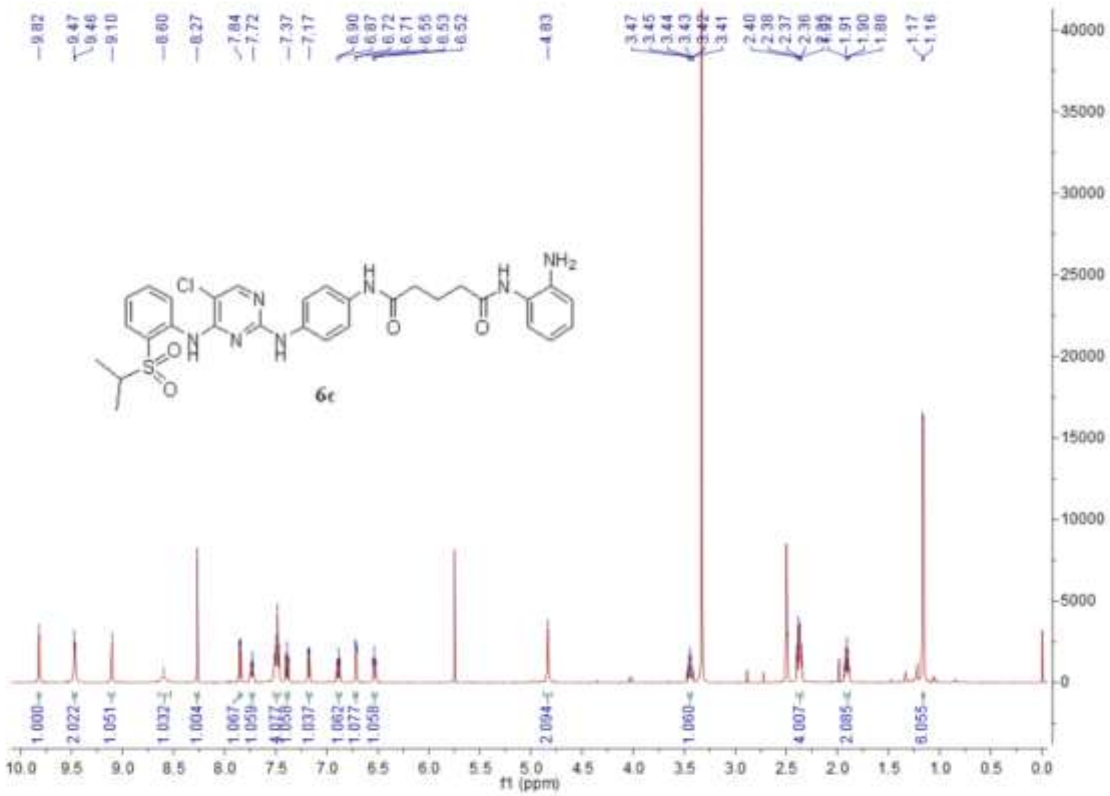

Figure 5. ^{1}H-NMR spectrum of **6c**

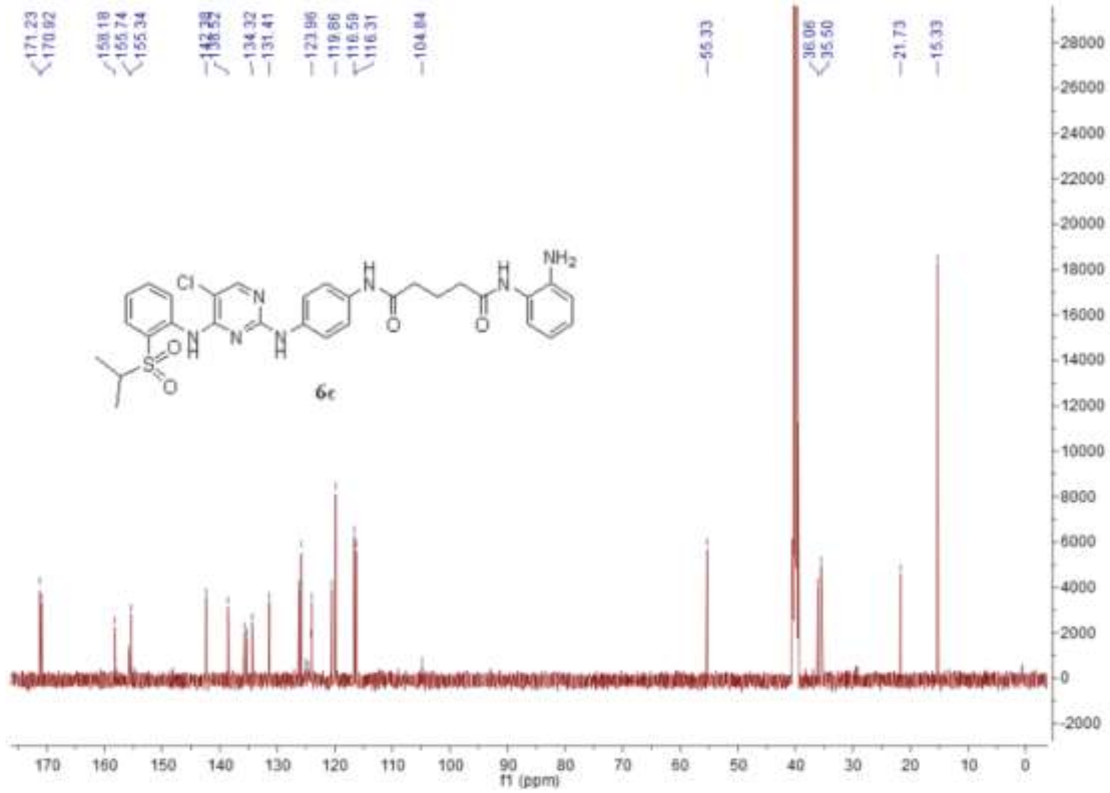

Figure 6. ^{13}C-NMR spectrum of **6c**

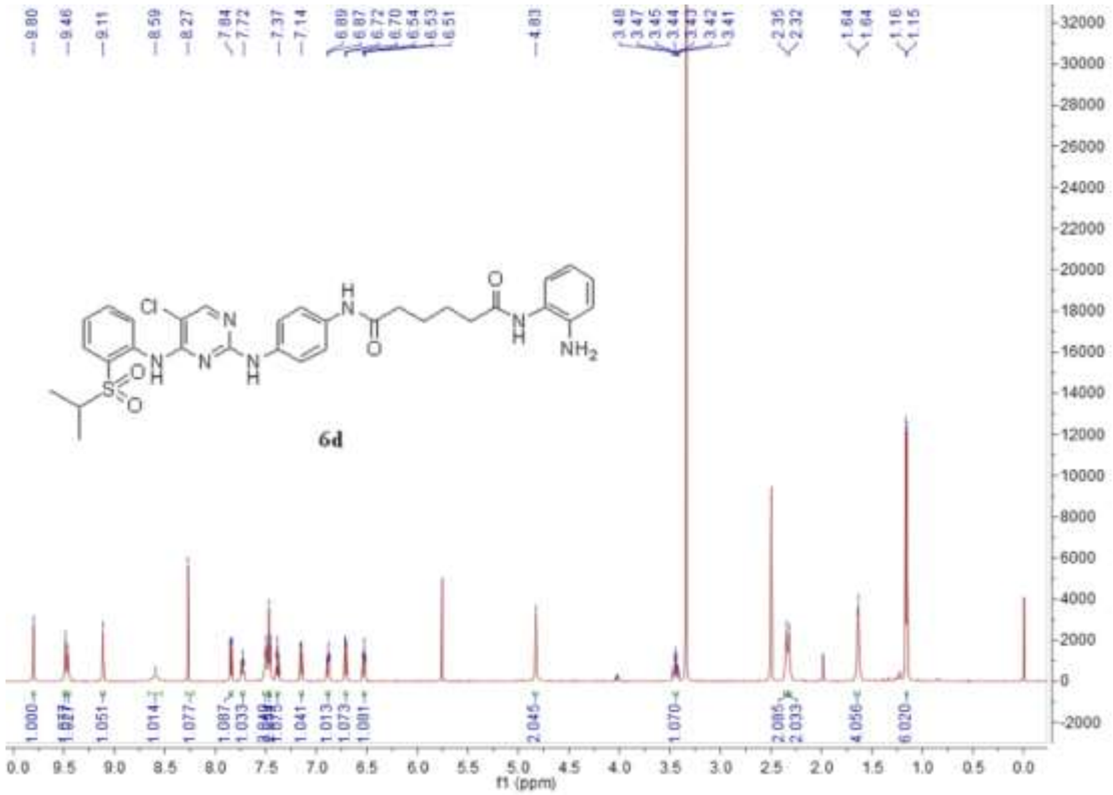

Figure 7. ^{1}H-NMR spectrum of **6d**

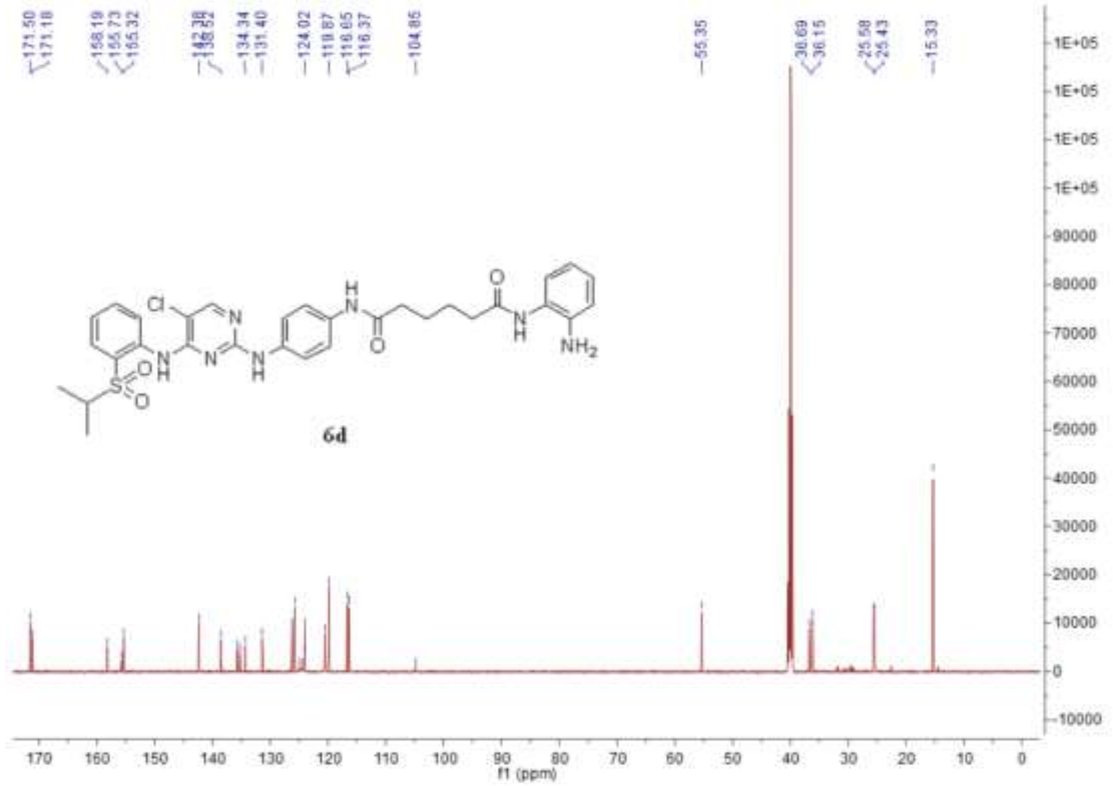

Figure 8. ^{13}C-NMR spectrum of **6d**

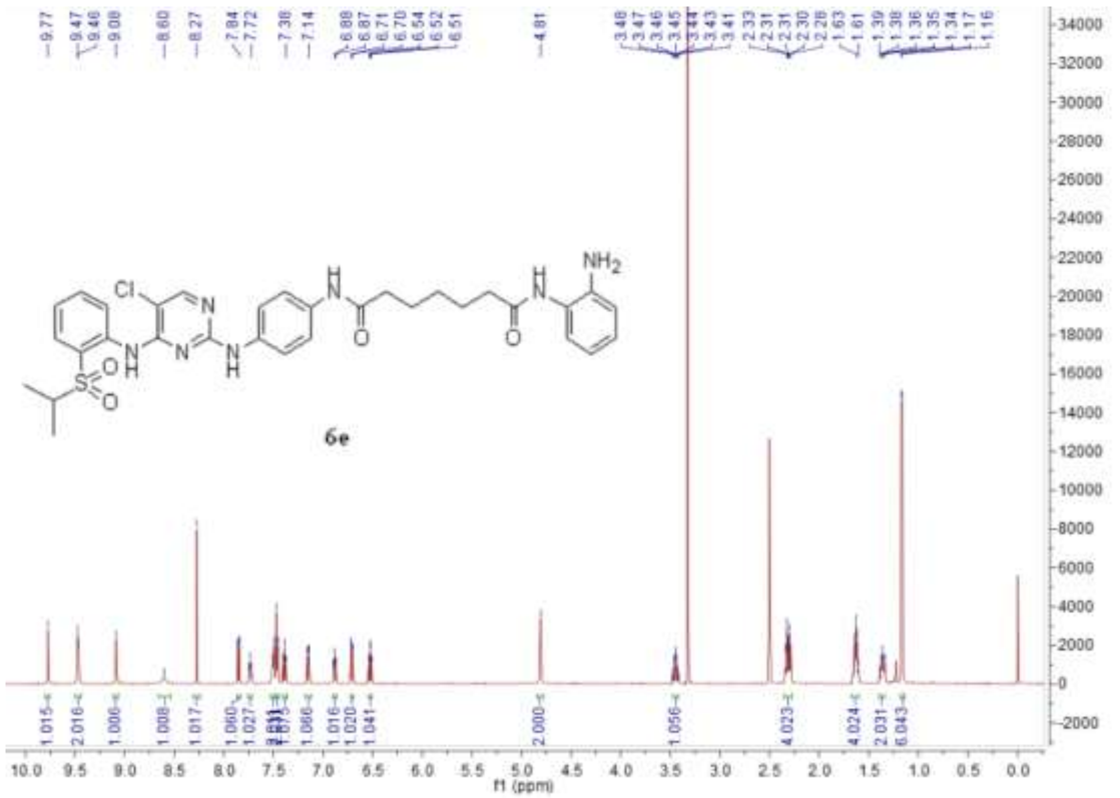

Figure 9. ^{1}H-NMR spectrum of **6e**

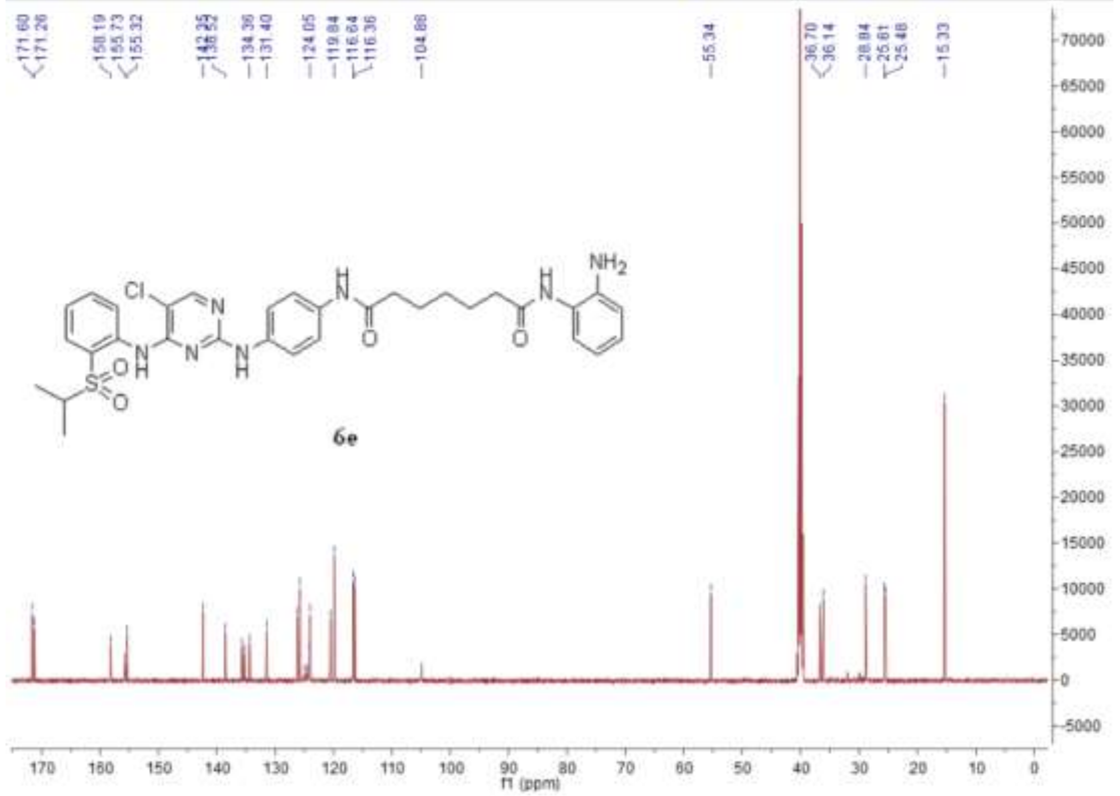

Figure 10. ^{13}C-NMR spectrum of **6e**

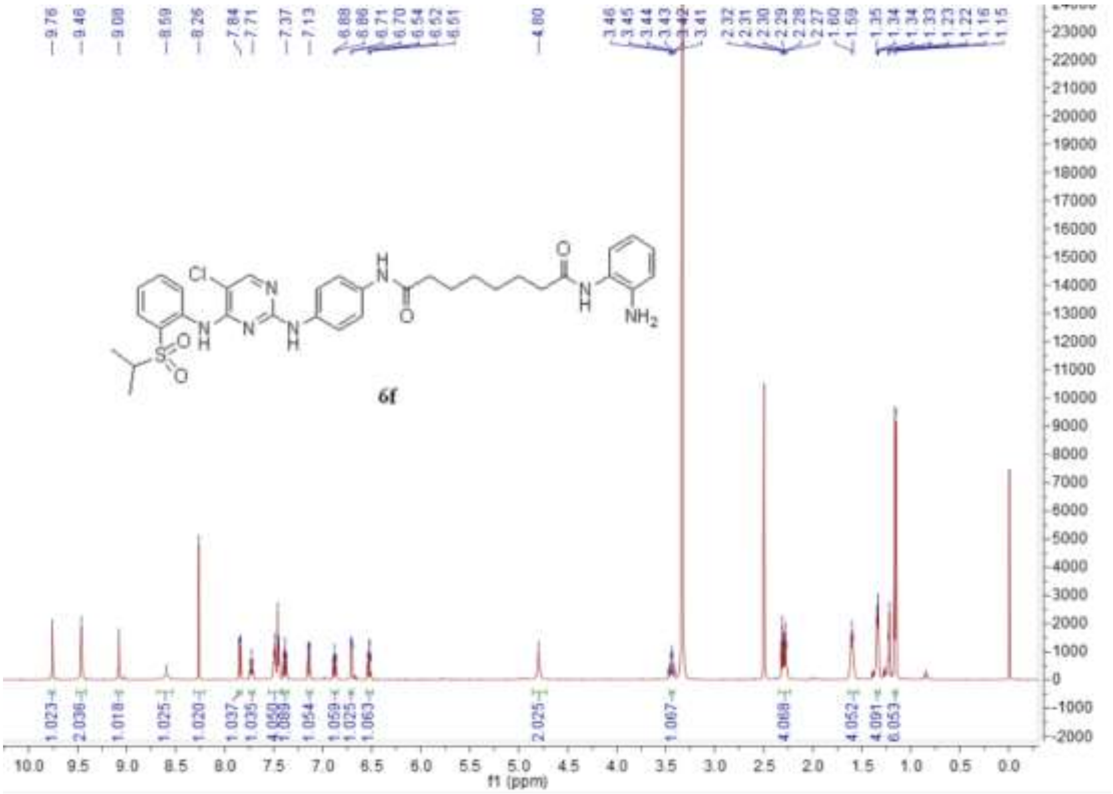

Figure 11. ^{1}H-NMR spectrum of **6f**

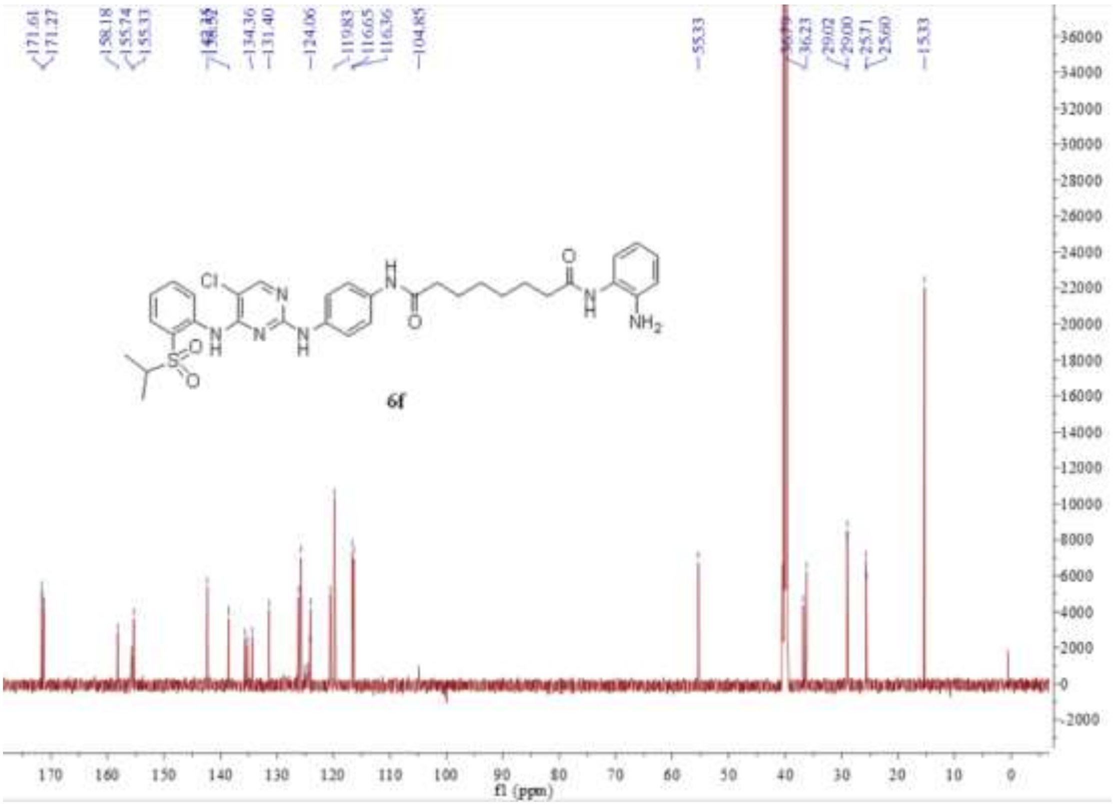

Figure 12. ^{13}C-NMR spectrum of **6f**

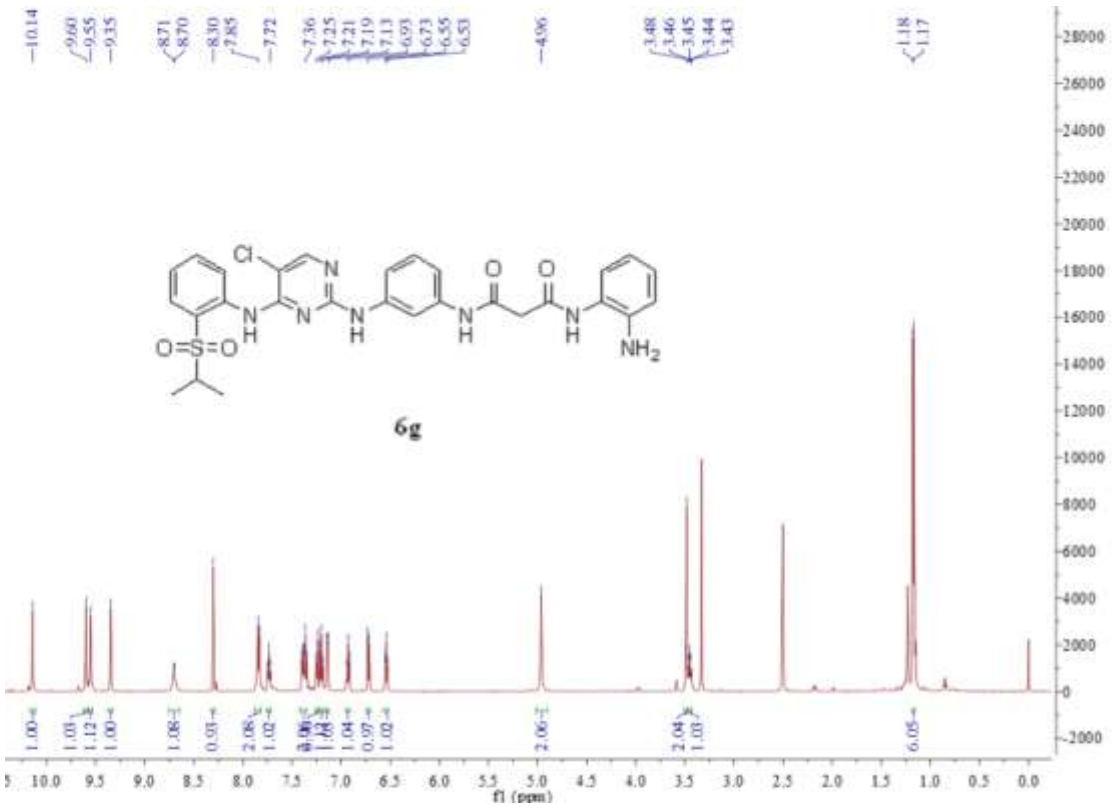

Figure 13. ^{1}H-NMR spectrum of **6g**

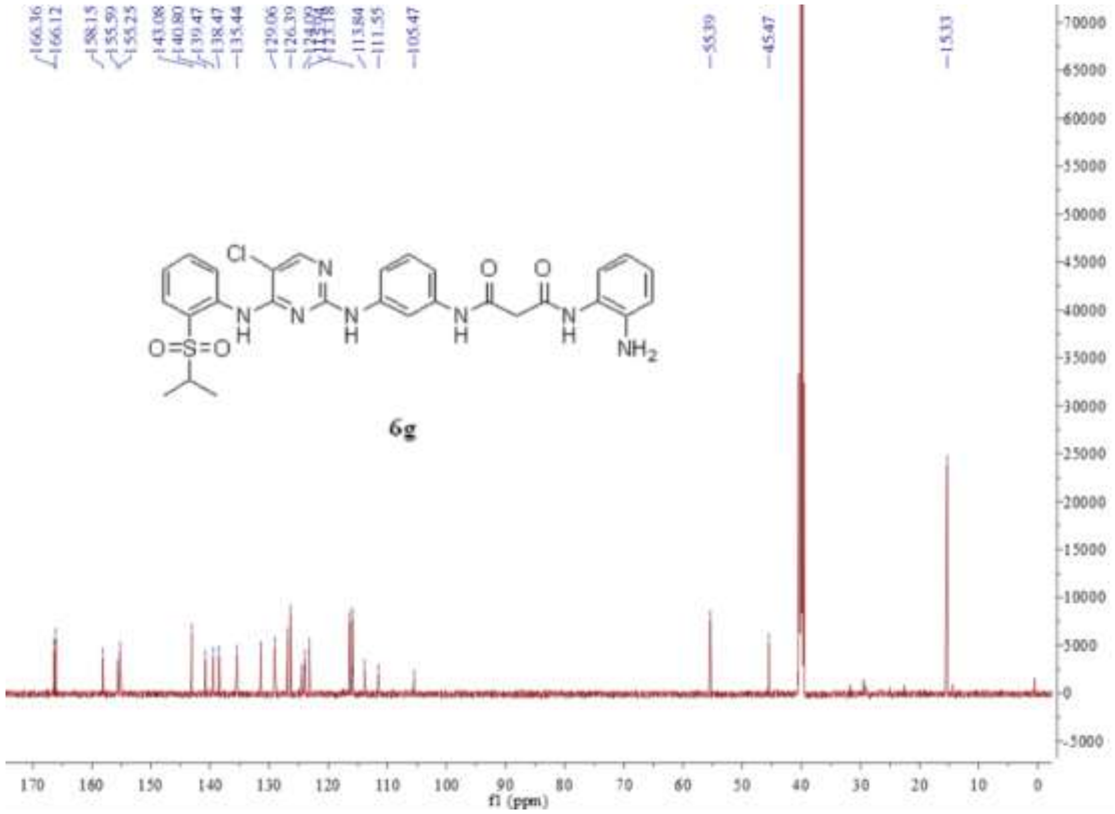

Figure 14. ^{13}C-NMR spectrum of **6g**

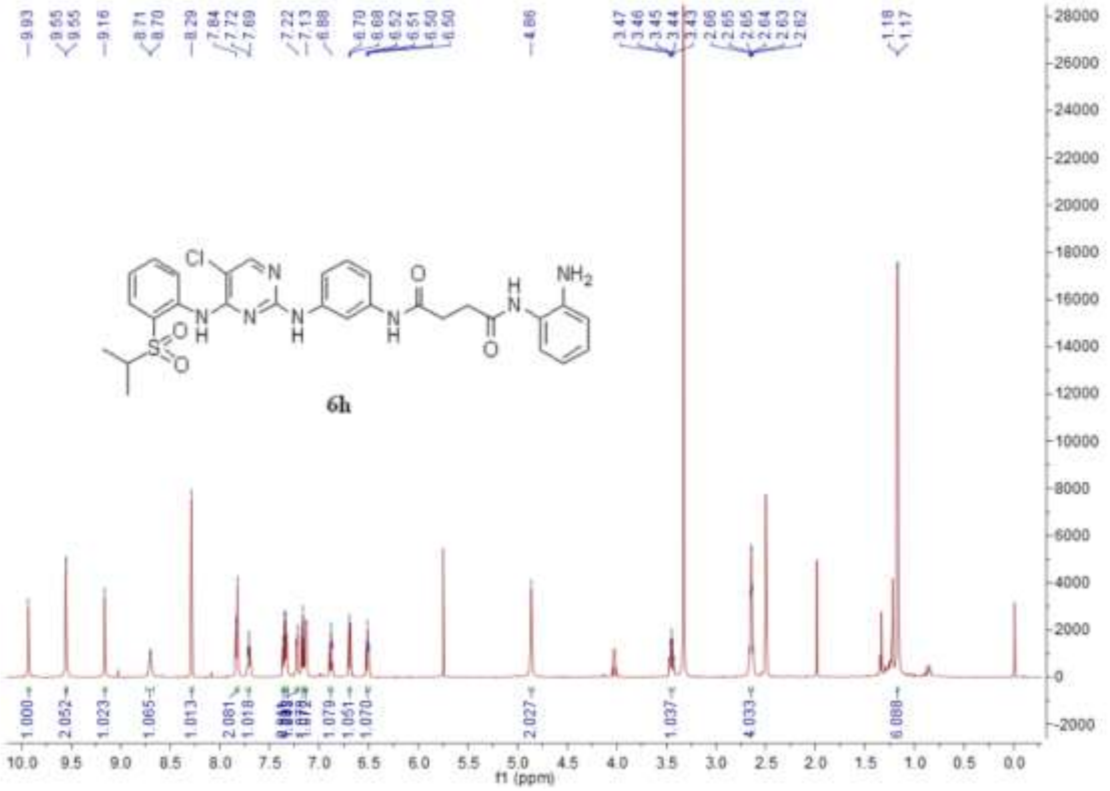

Figure 15. ^{1}H-NMR spectrum of **6h**

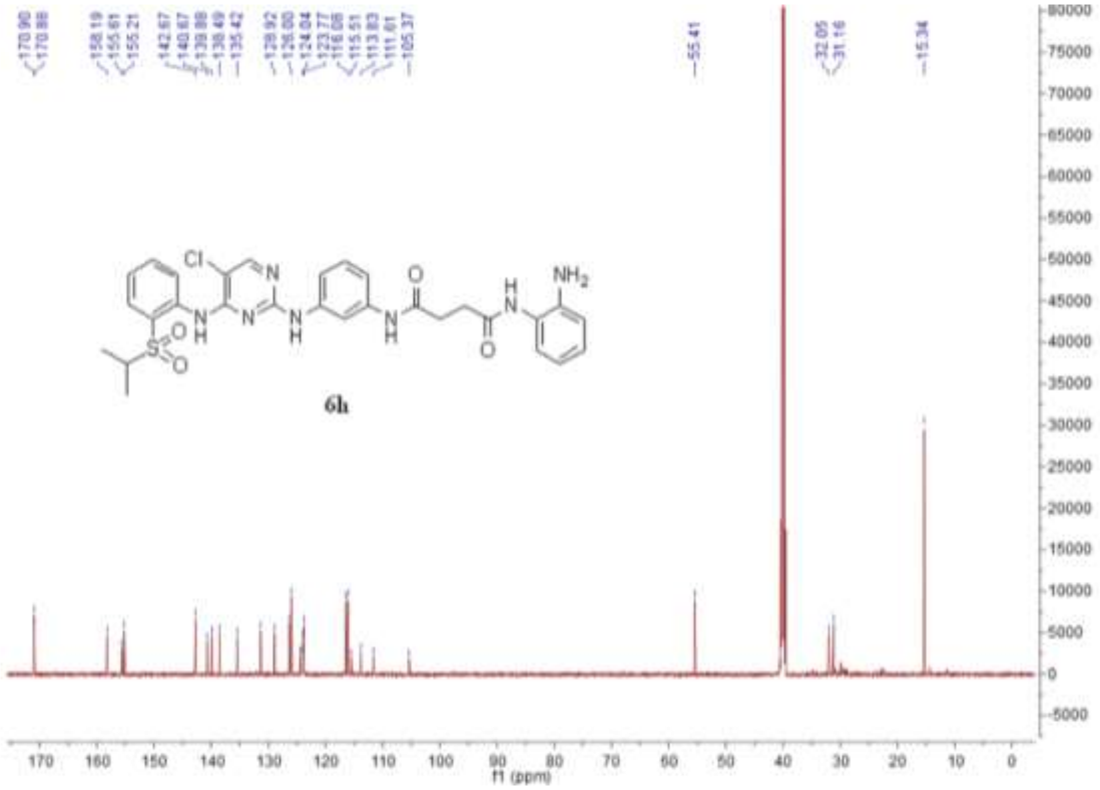

Figure 16. ^{13}C-NMR spectrum of **6h**

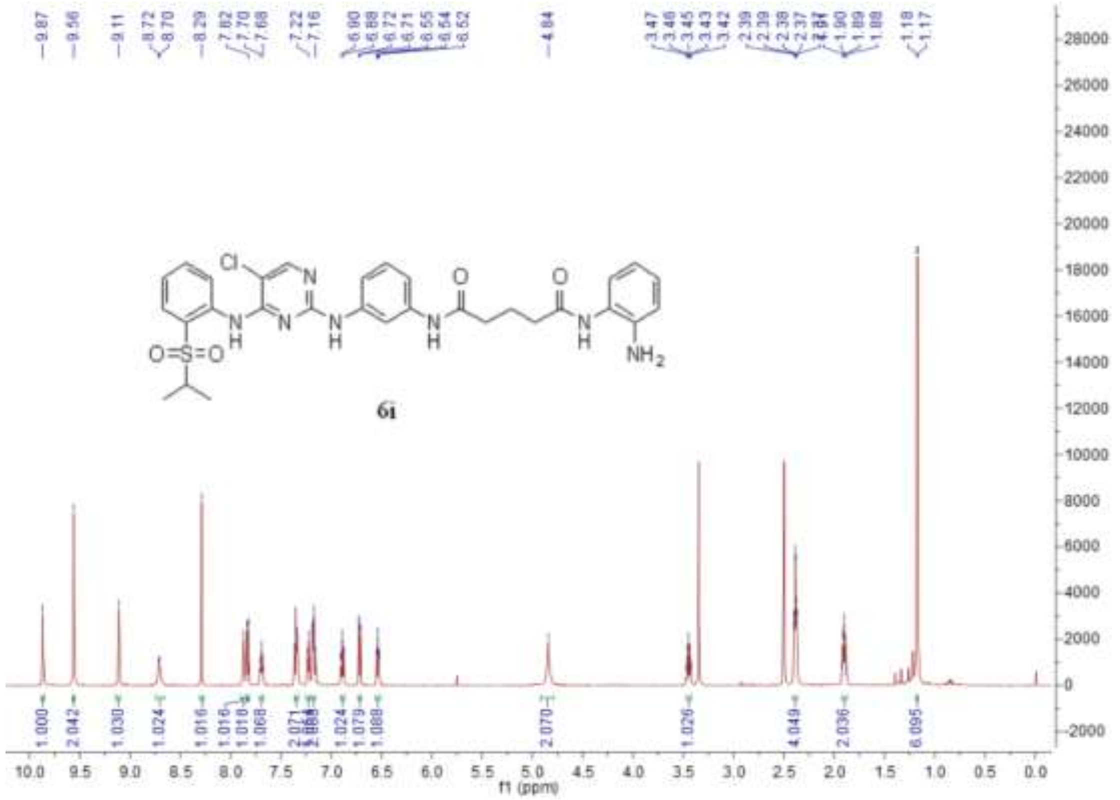

Figure 17. ^{1}H-NMR spectrum of **6i**

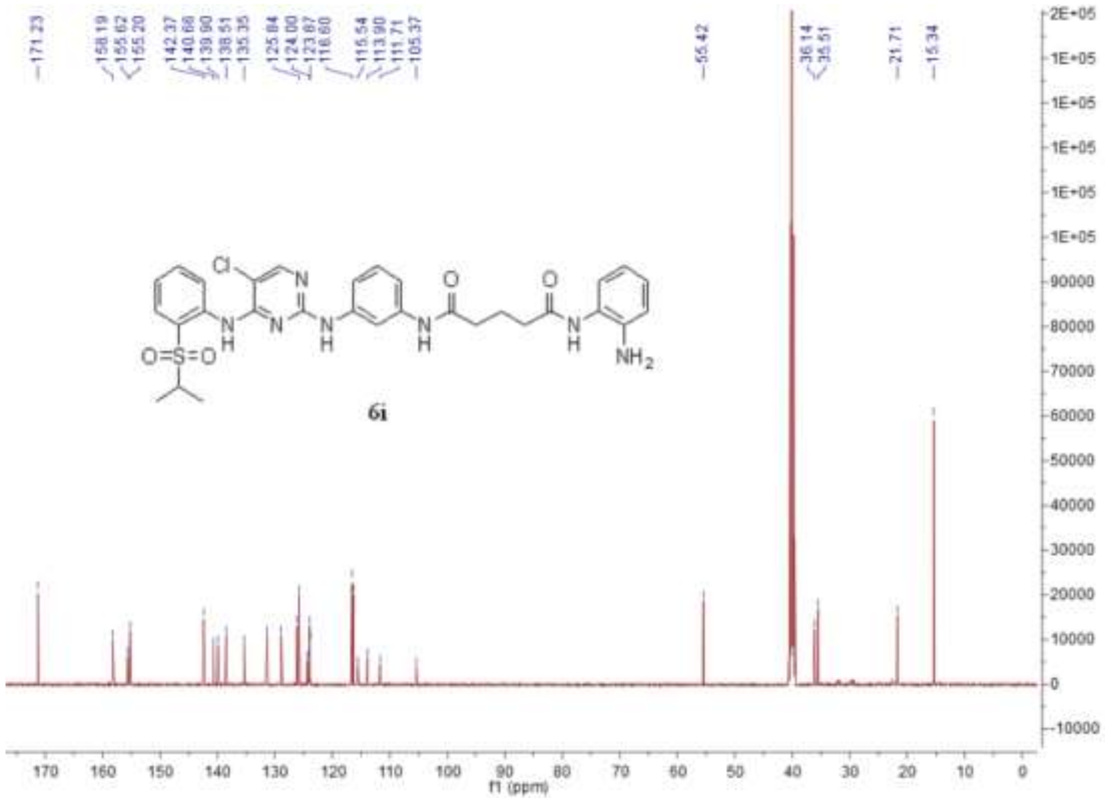

Figure 18. ^{13}C-NMR spectrum of **6i**

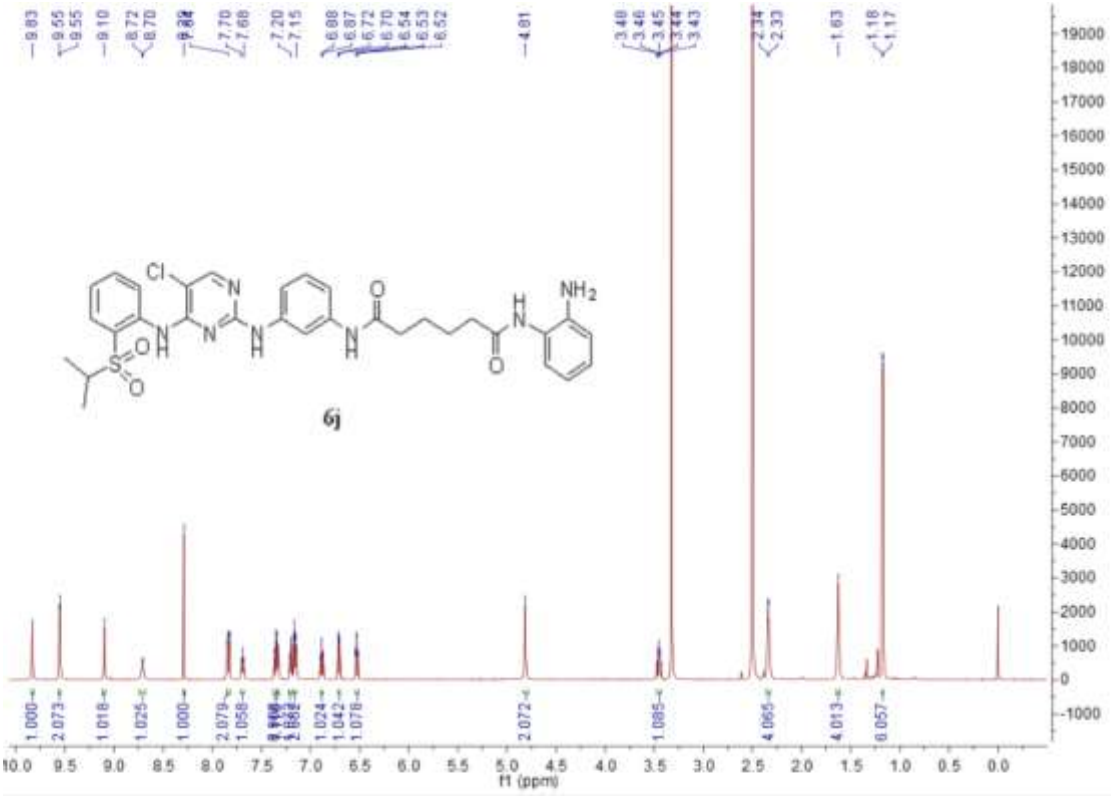

Figure 19. ^{1}H-NMR spectrum of **6j**

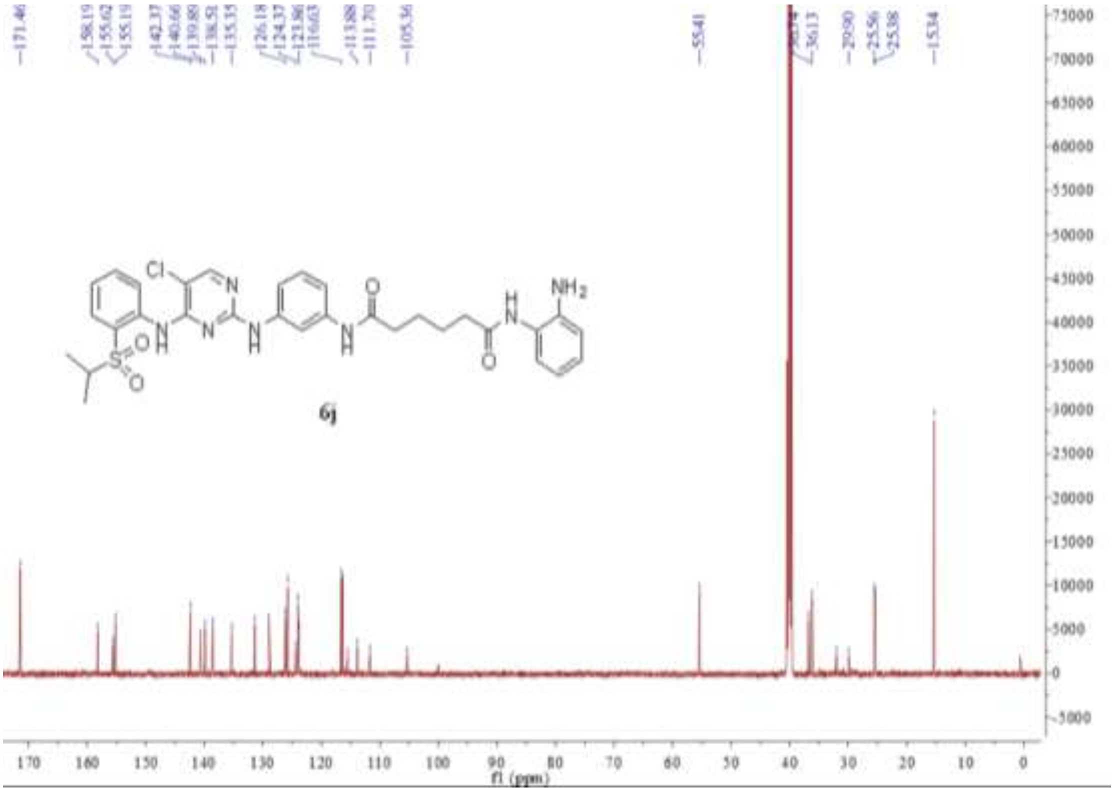

Figure 20. ^{13}C-NMR spectrum of **6j**

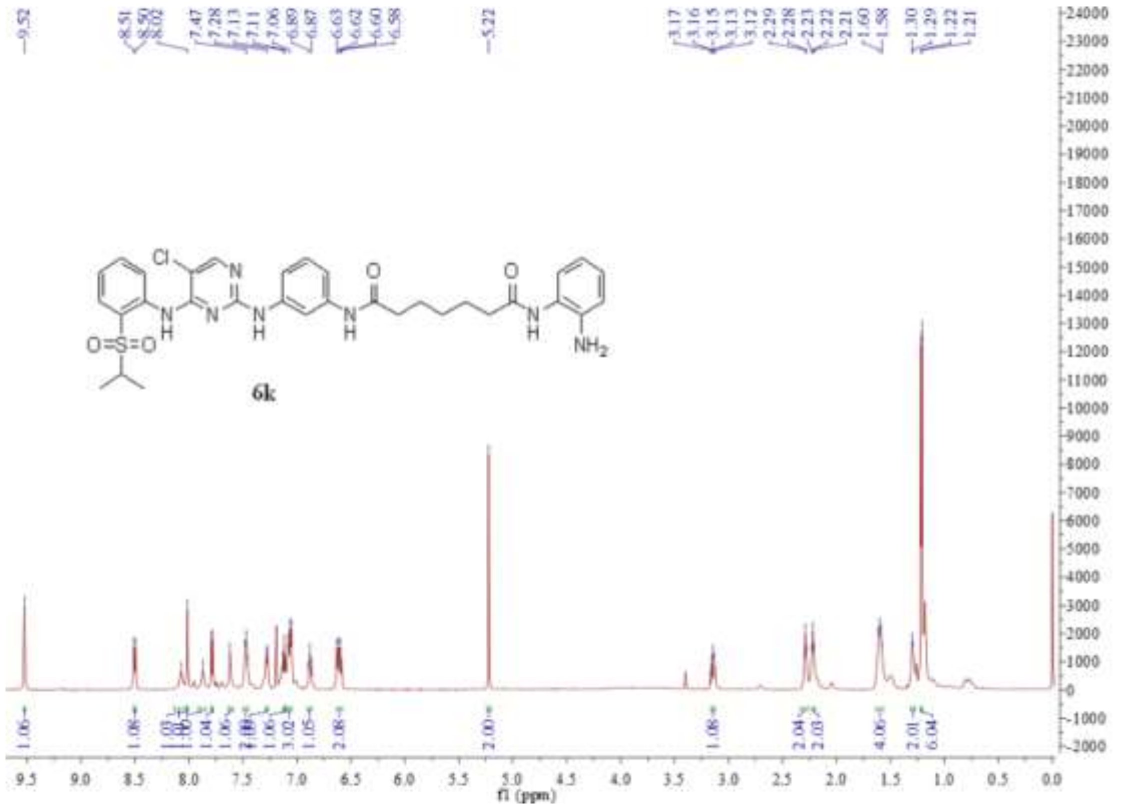

Figure 21. ^{1}H-NMR spectrum of **6k**

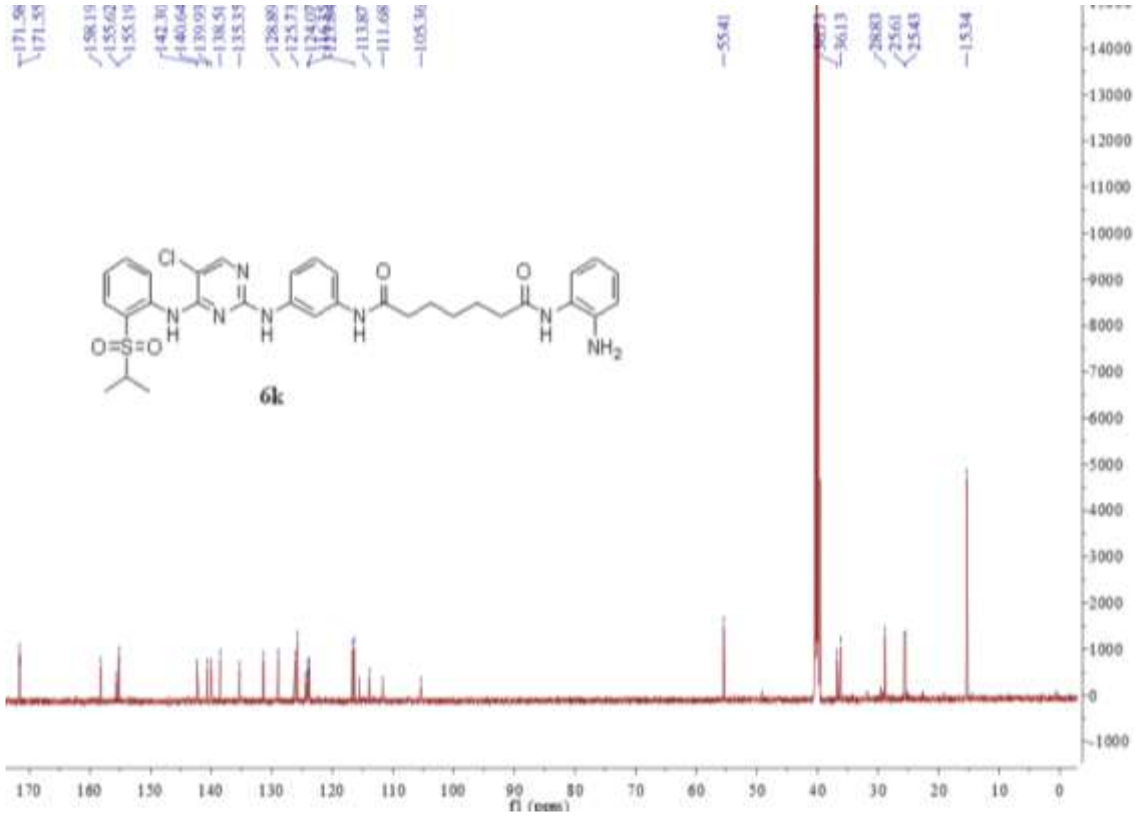

Figure 22. ^{13}C-NMR spectrum of **6k**

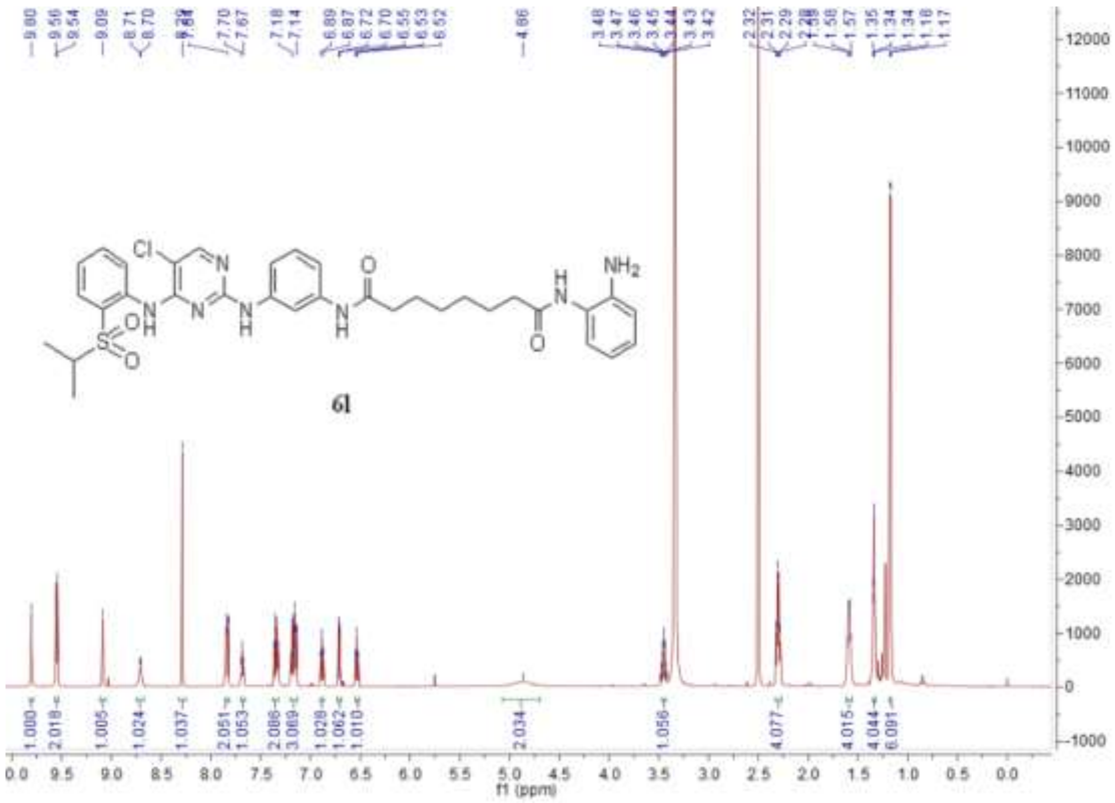

Figure 23. ^{1}H-NMR spectrum of **6l**

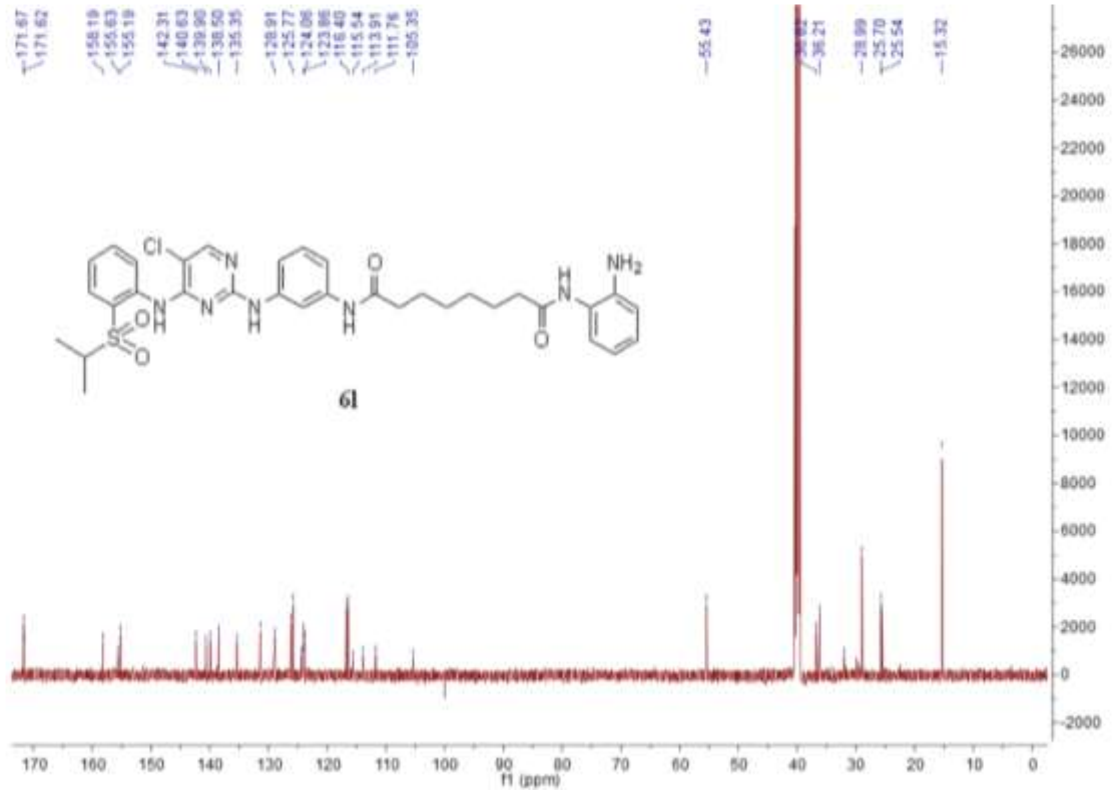

Figure 24. ^{13}C-NMR spectrum of **6l**

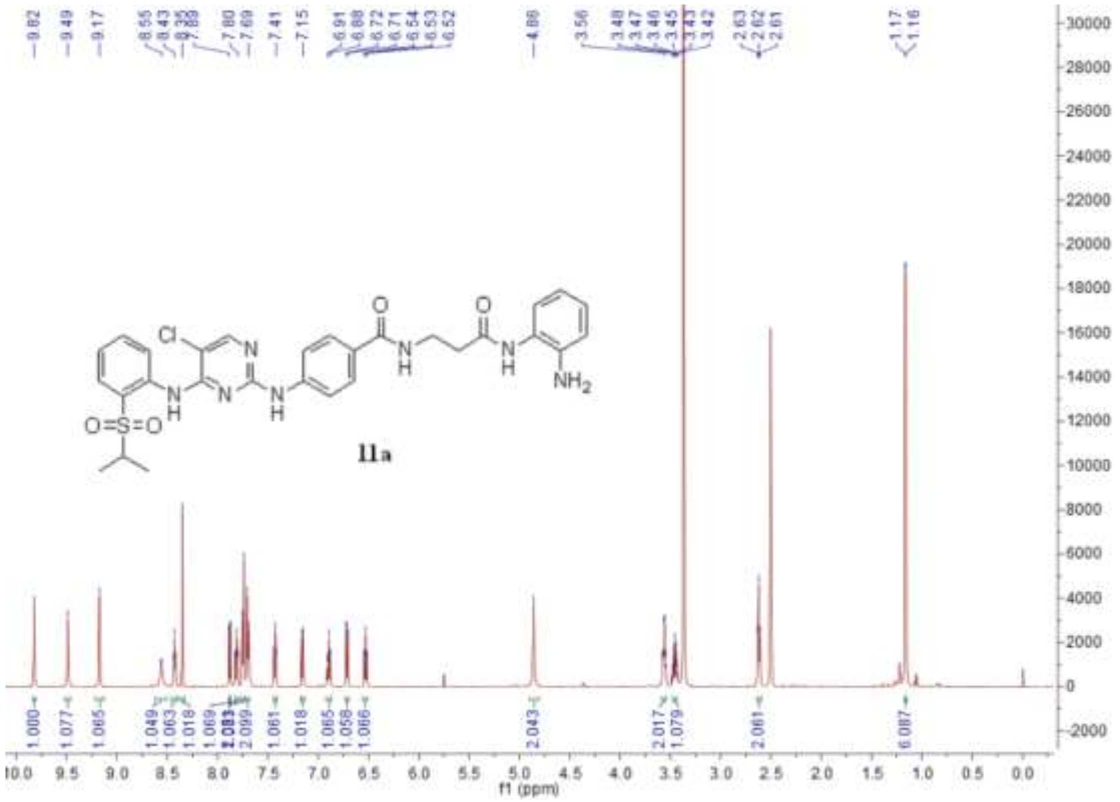

Figure 25. ^{1}H-NMR spectrum of **11a**

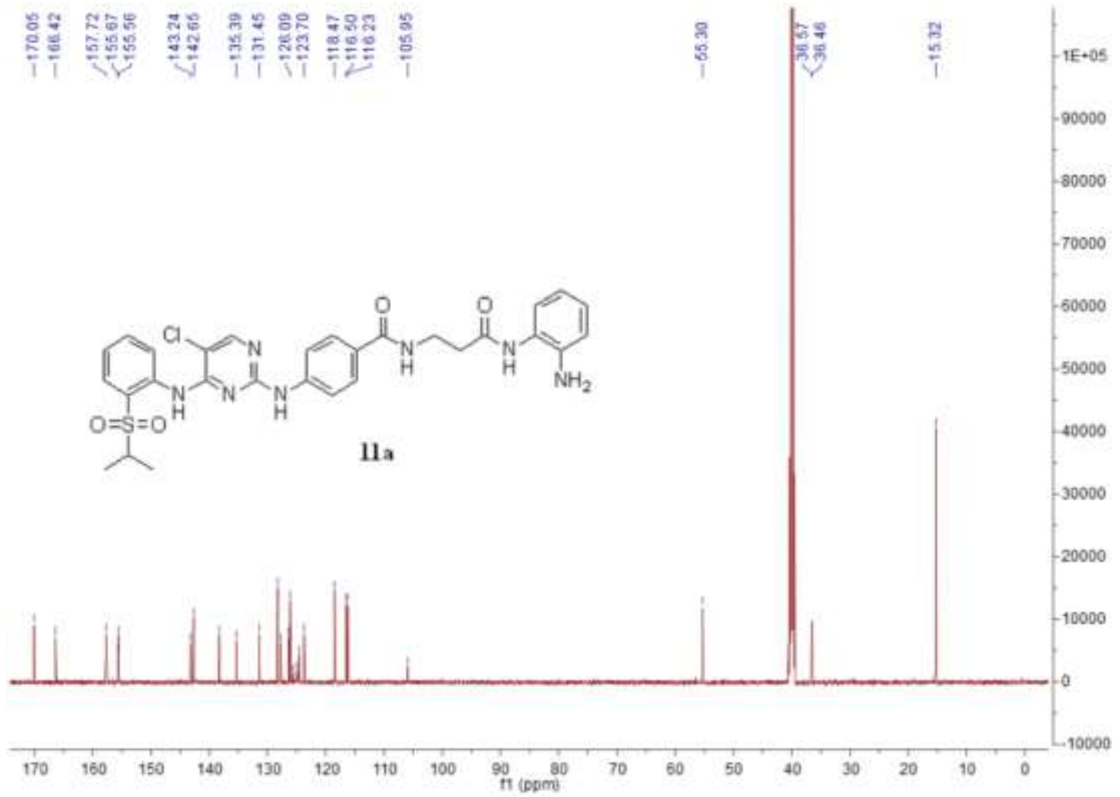

Figure 26. ^{13}C-NMR spectrum of **11a**

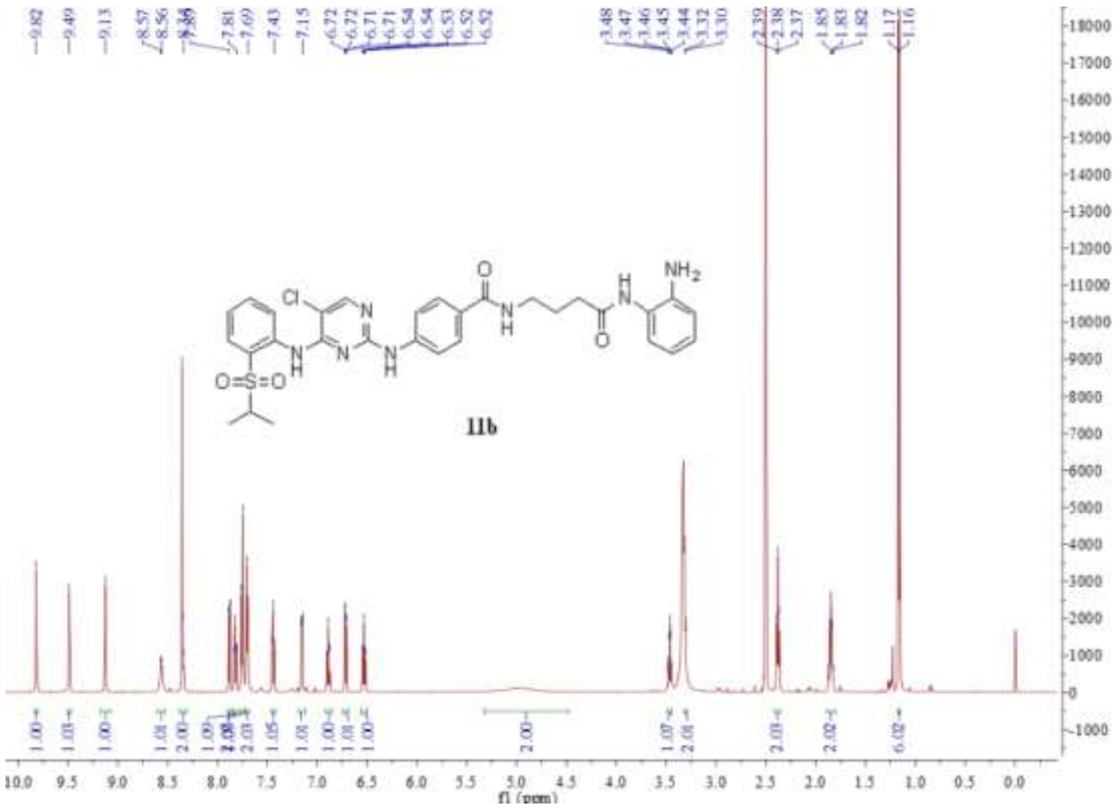

Figure 27. ^{1}H-NMR spectrum of **11b**

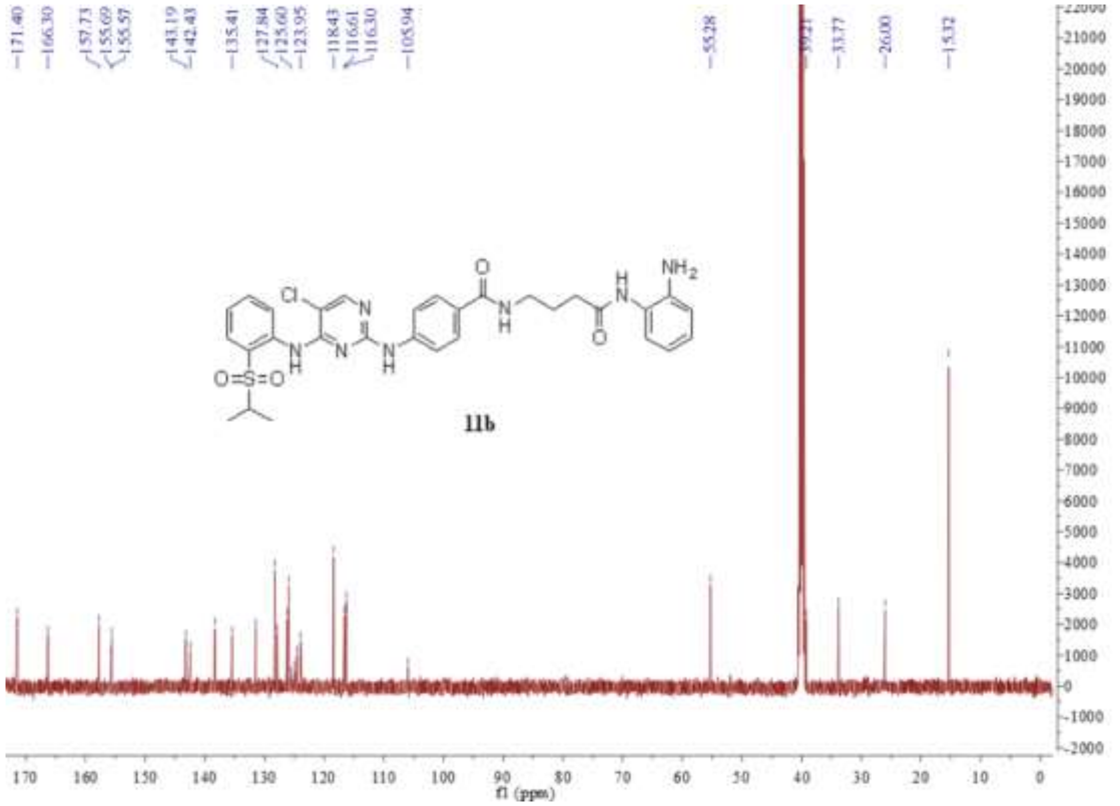

Figure 28. ^{13}C-NMR spectrum of **11b**

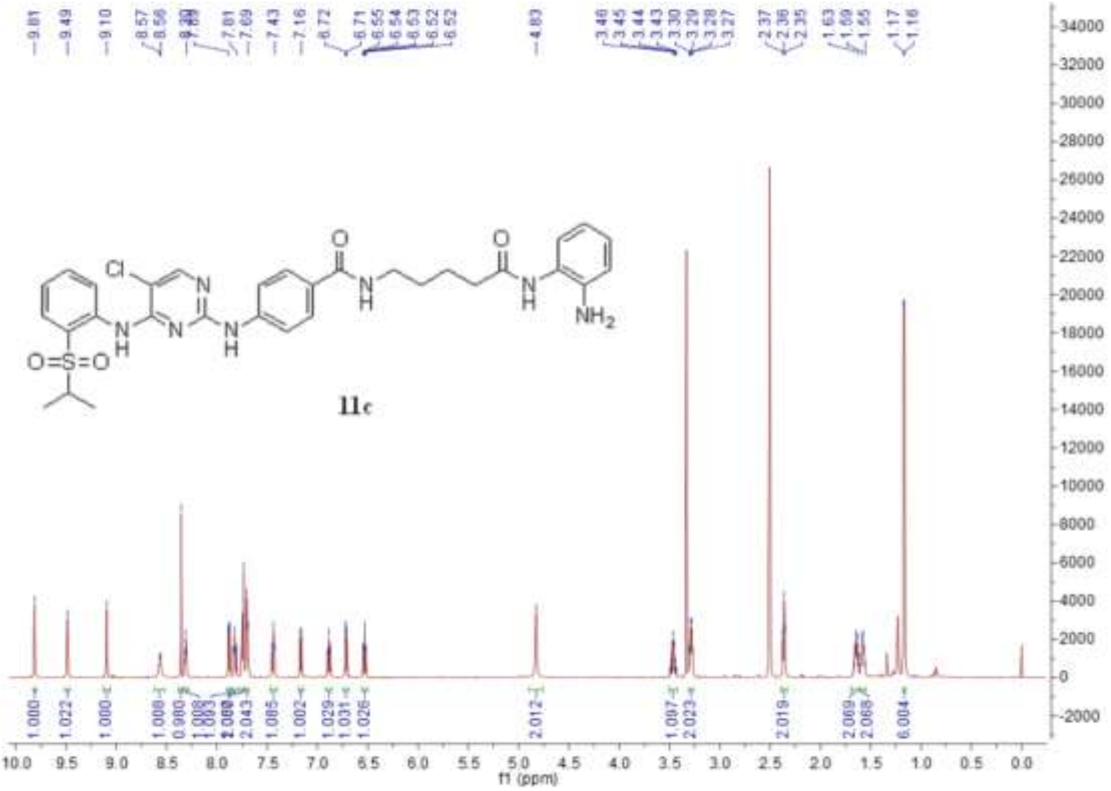

Figure 29. ^{1}H-NMR spectrum of **11c**

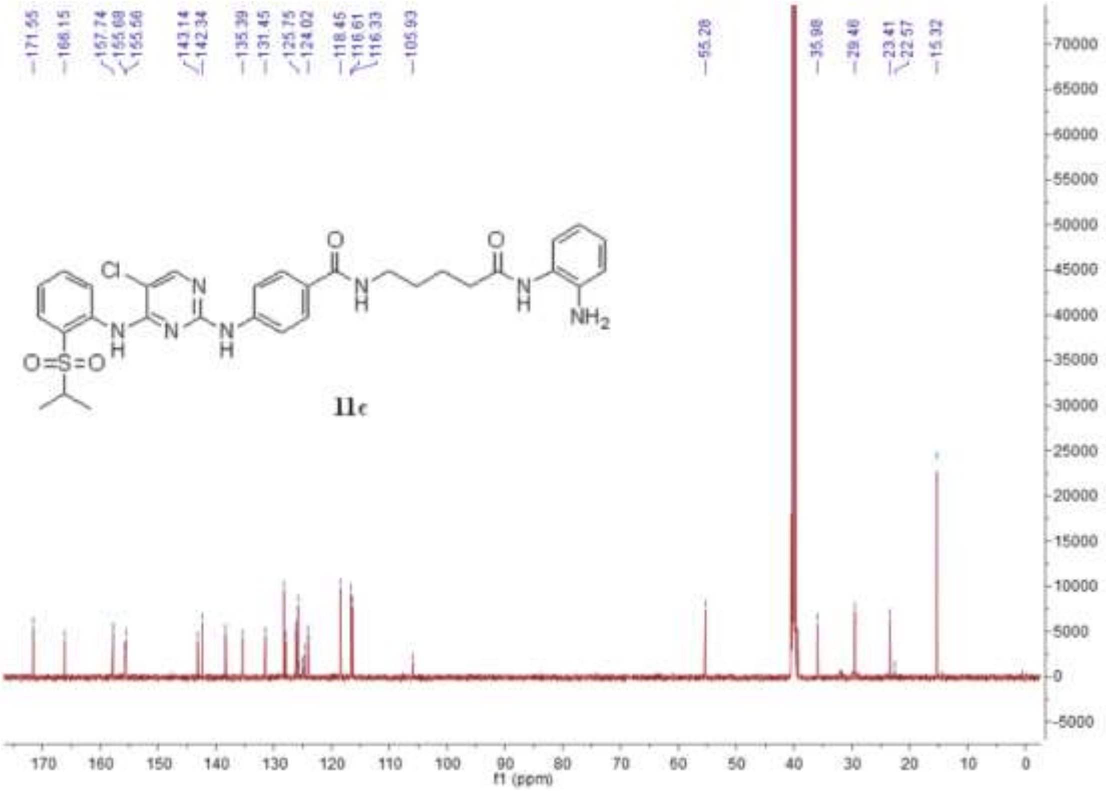

Figure 30. ^{13}C-NMR spectrum of **11c**

Information Classification: General

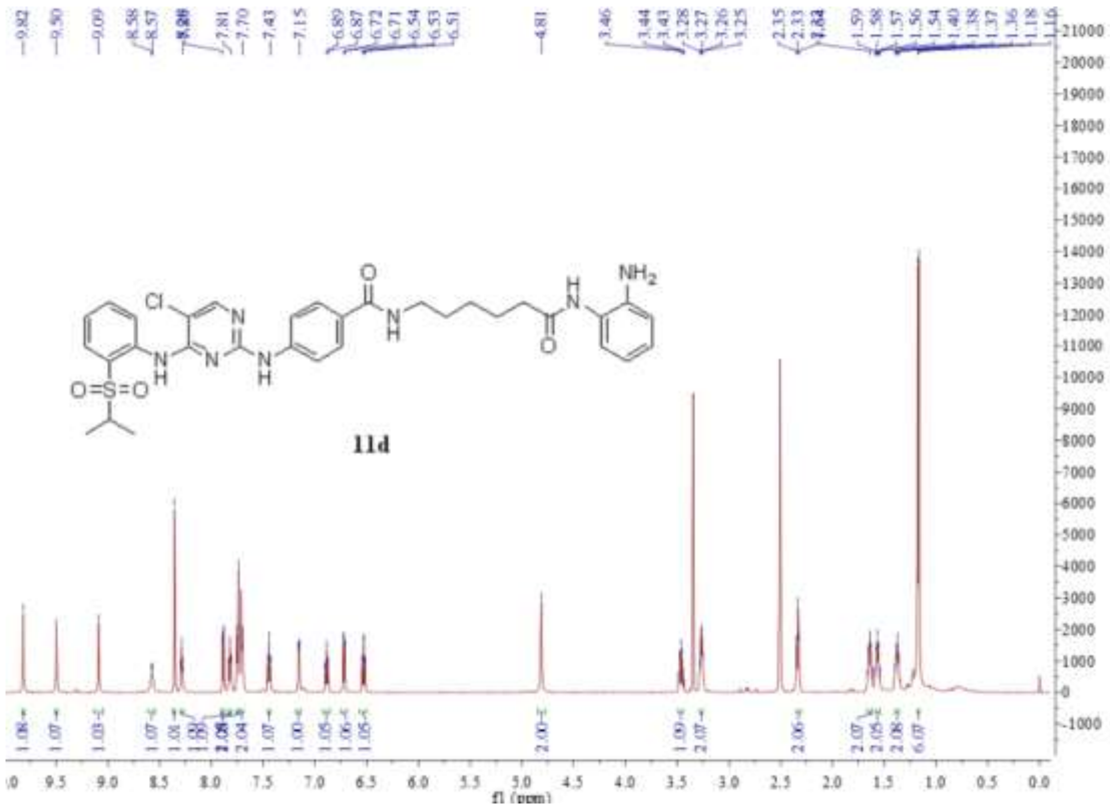

Figure 31. ^{1}H-NMR spectrum of **11d**

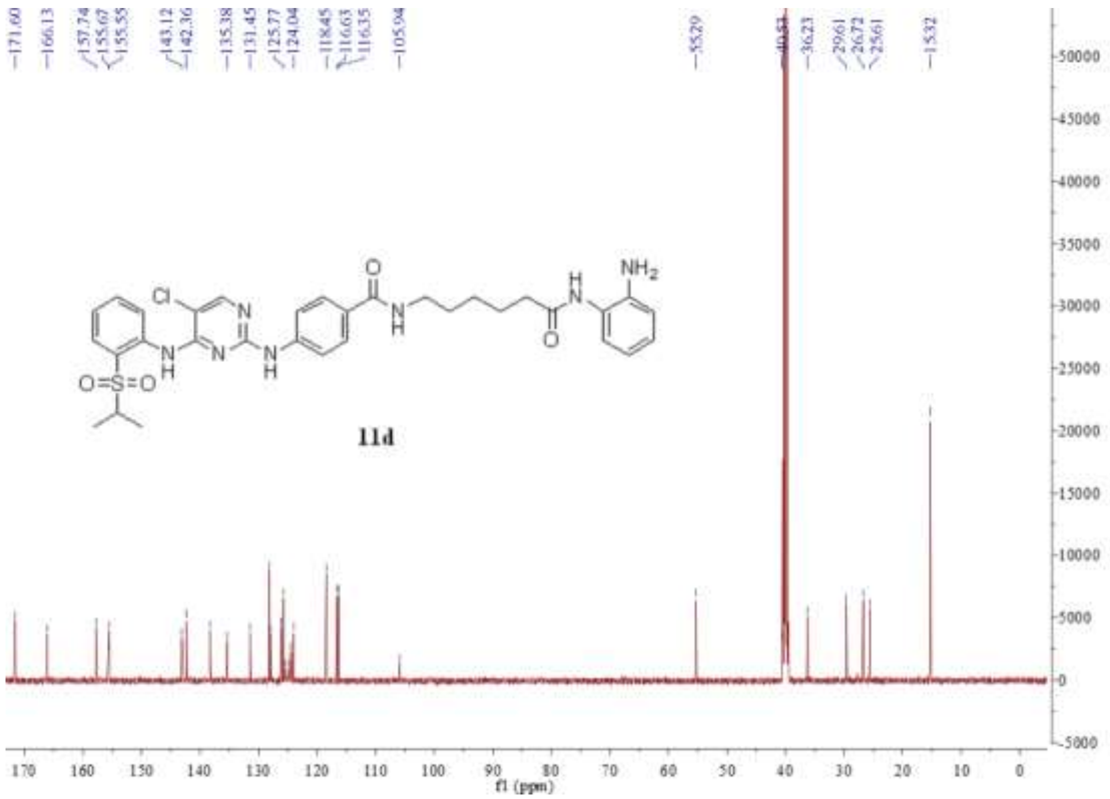

Figure 32. ^{13}C-NMR spectrum of **11d**

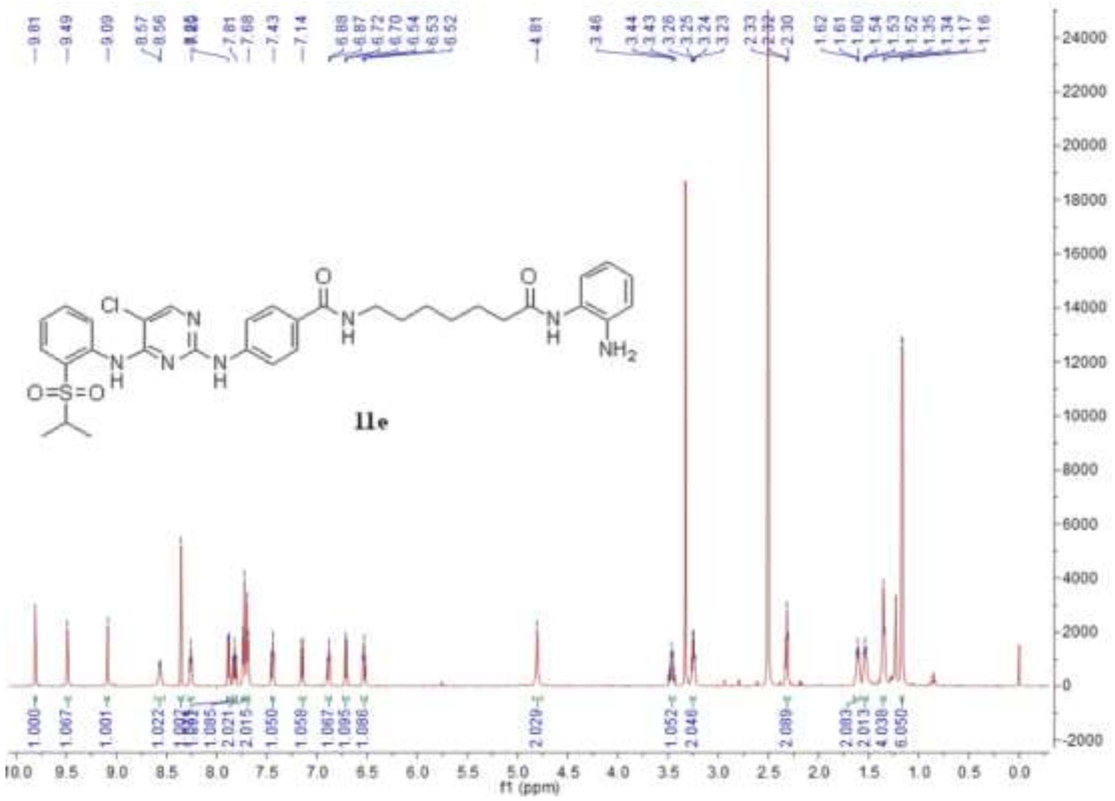

Figure 33. ^{1}H-NMR spectrum of **11e**

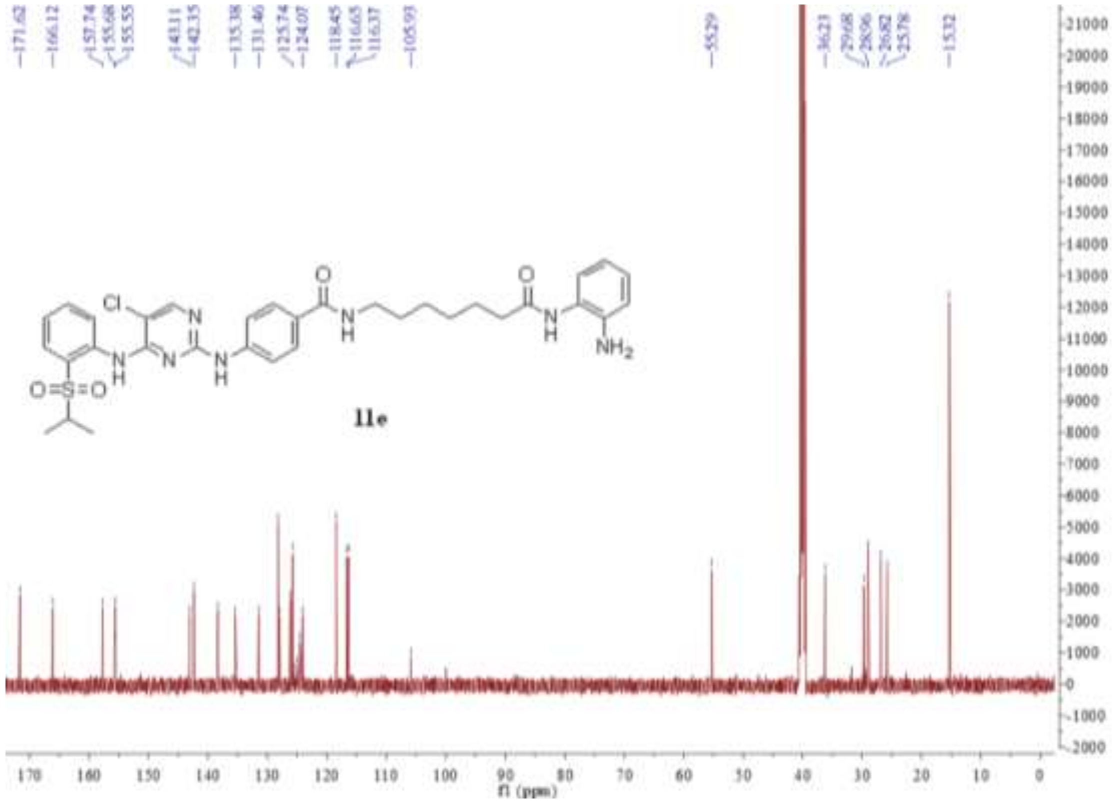

Figure 34. ^{13}C-NMR spectrum of **11e**

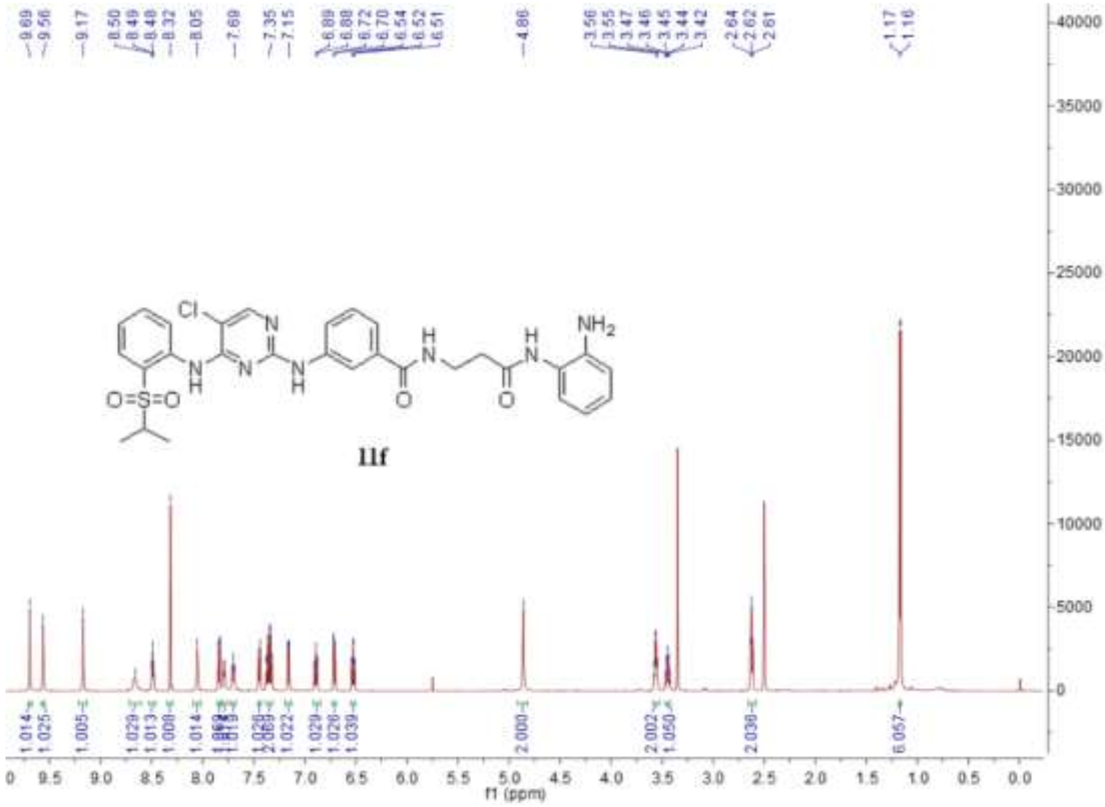

Figure 35. ^{1}H-NMR spectrum of **11f**

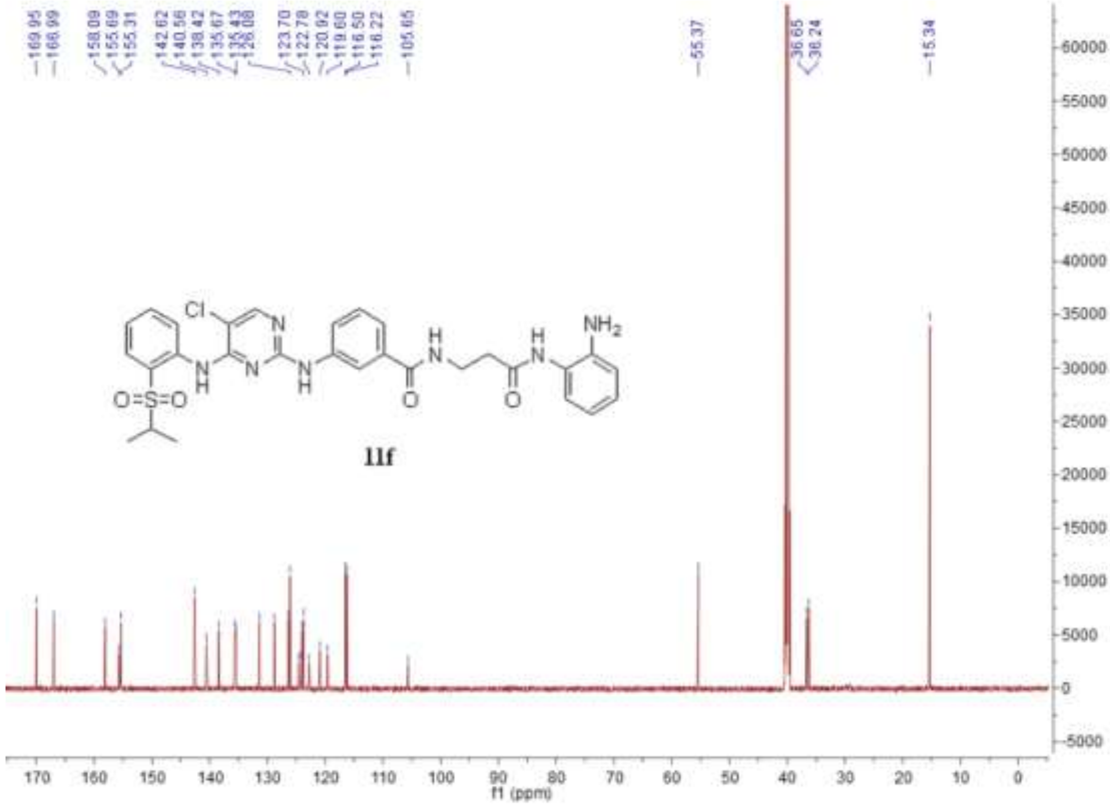

Figure 36. ^{13}C-NMR spectrum of **11f**

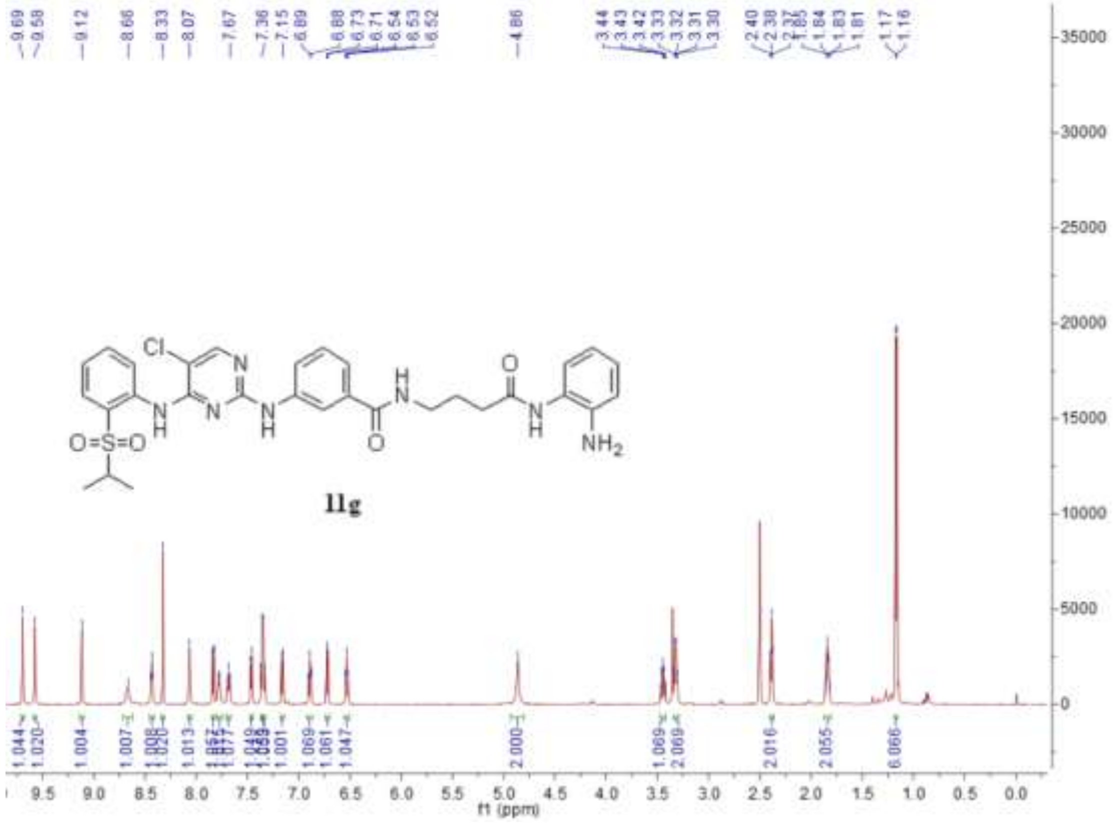

Figure 37. ^{1}H-NMR spectrum of **11g**

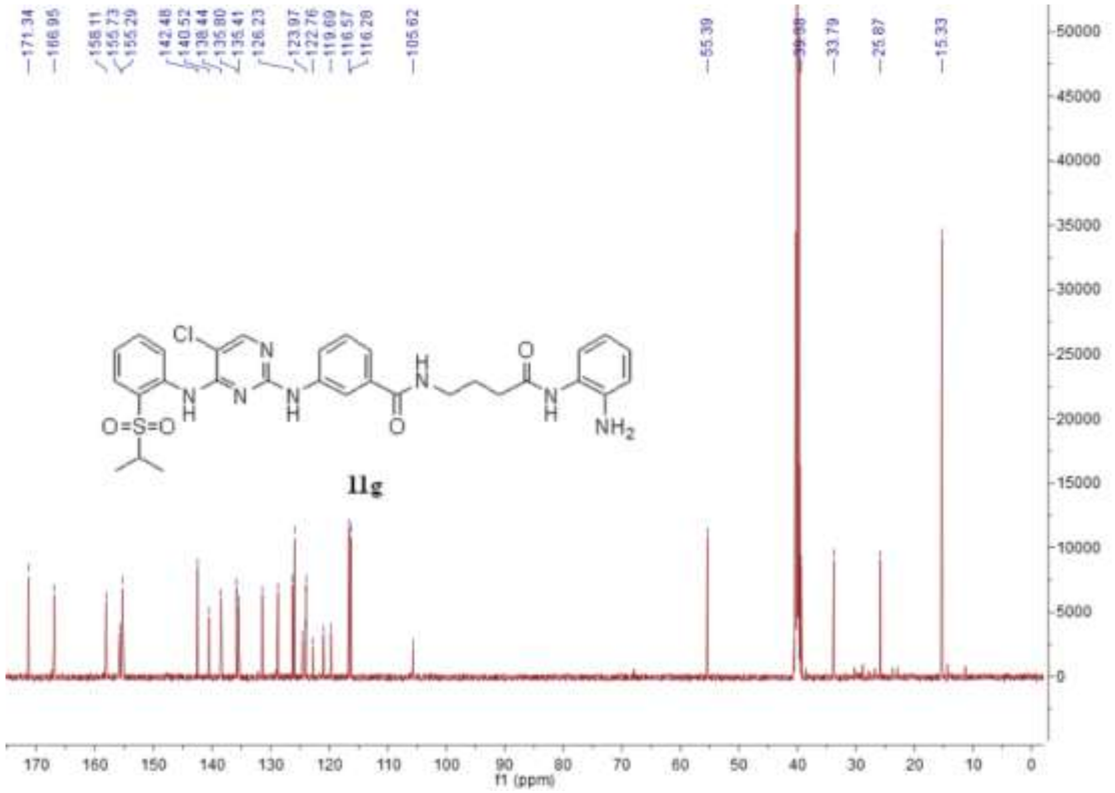

Figure 38. ^{13}C-NMR spectrum of **11g**

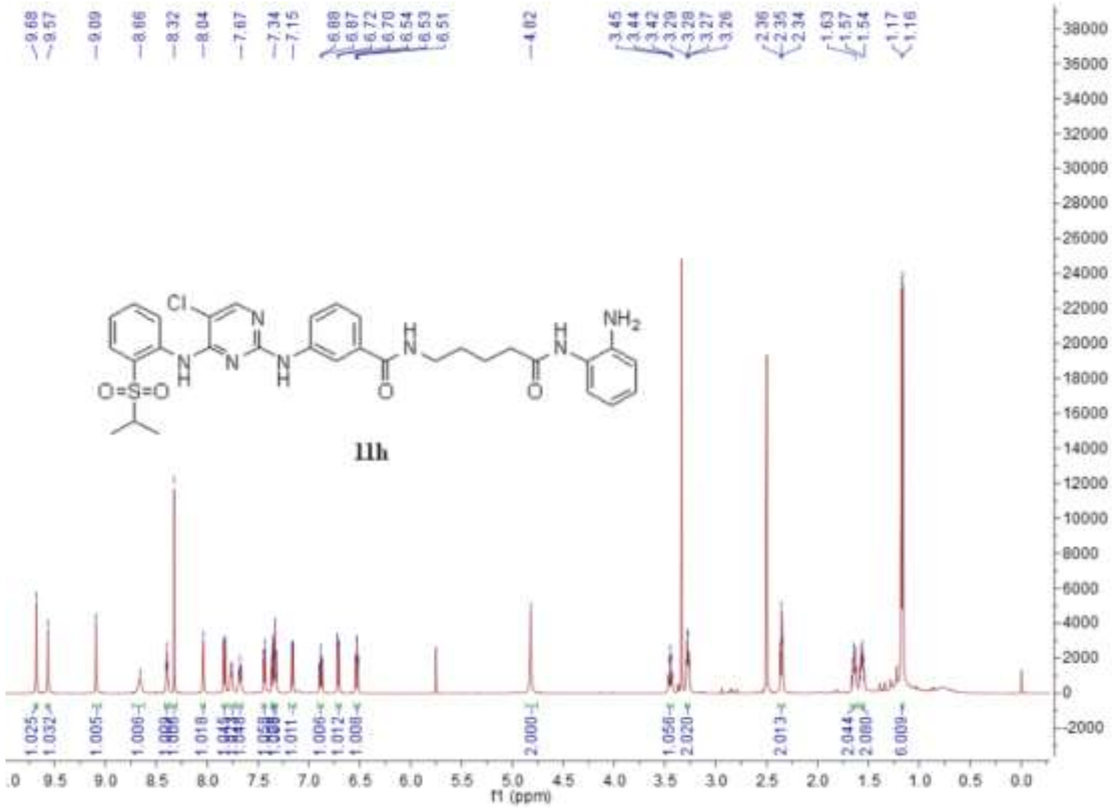

Figure 39. ^{1}H-NMR spectrum of **11h**

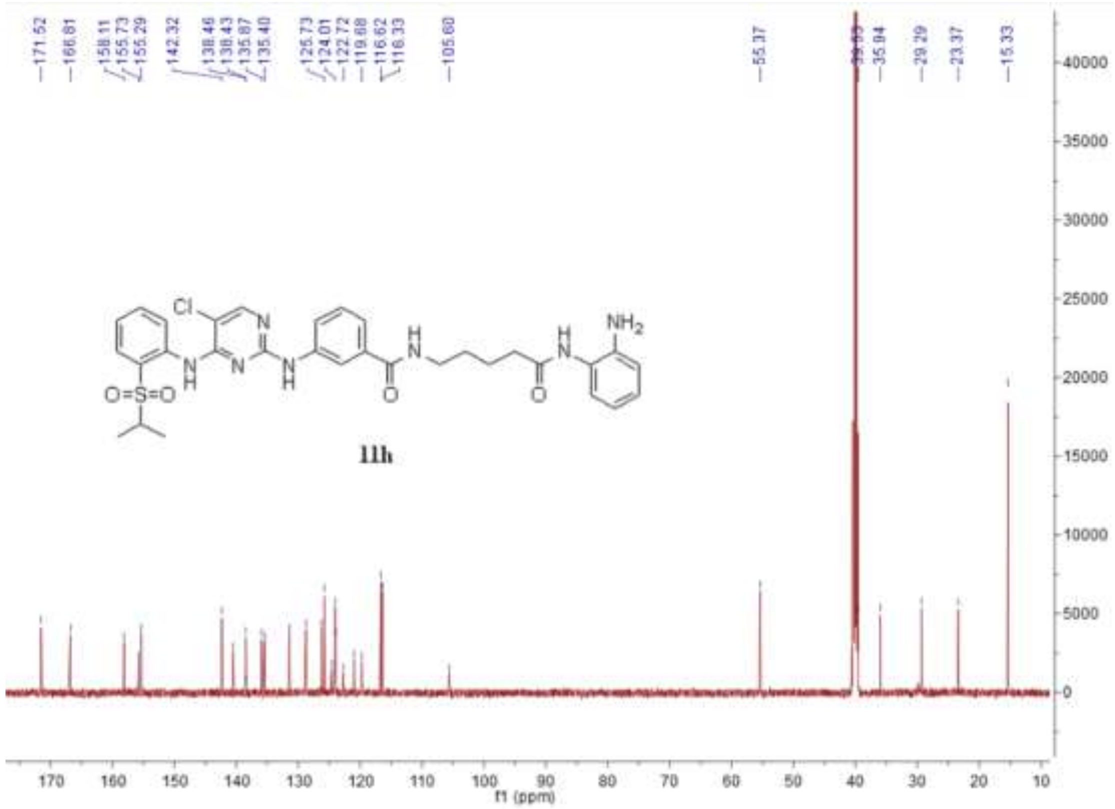

Figure 40. ^{13}C-NMR spectrum of **11h**

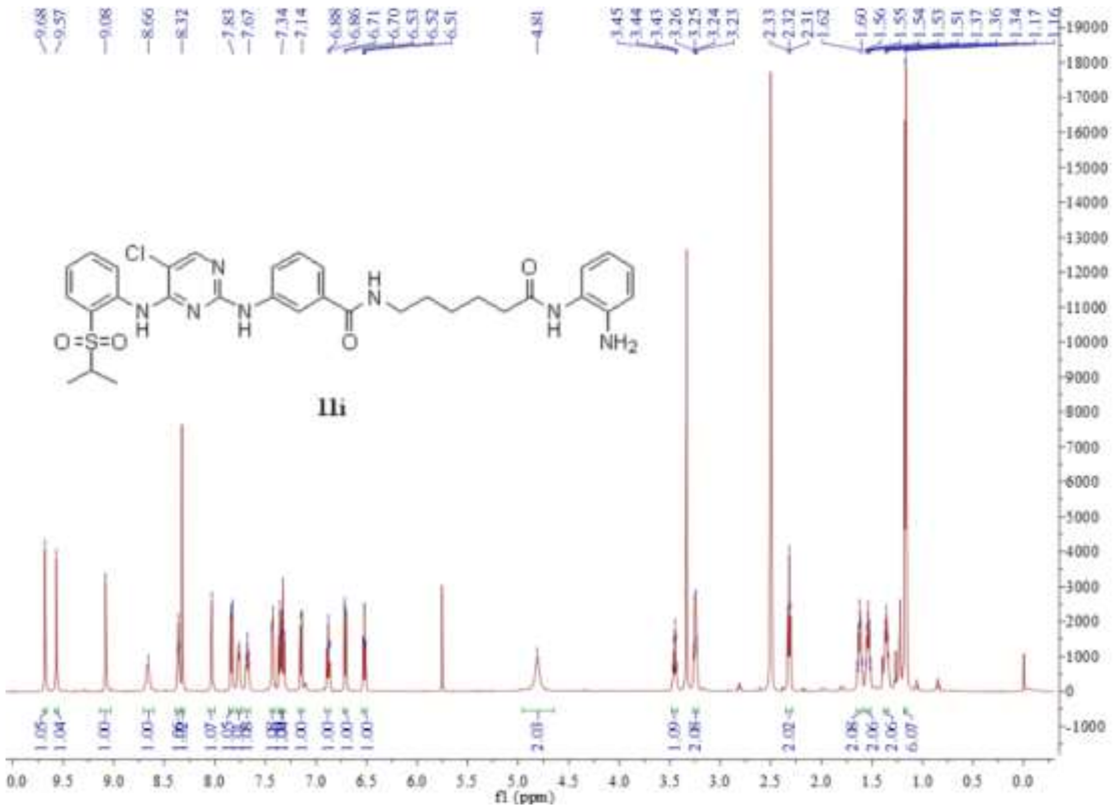

Figure 41. ^{1}H-NMR spectrum of **11i**

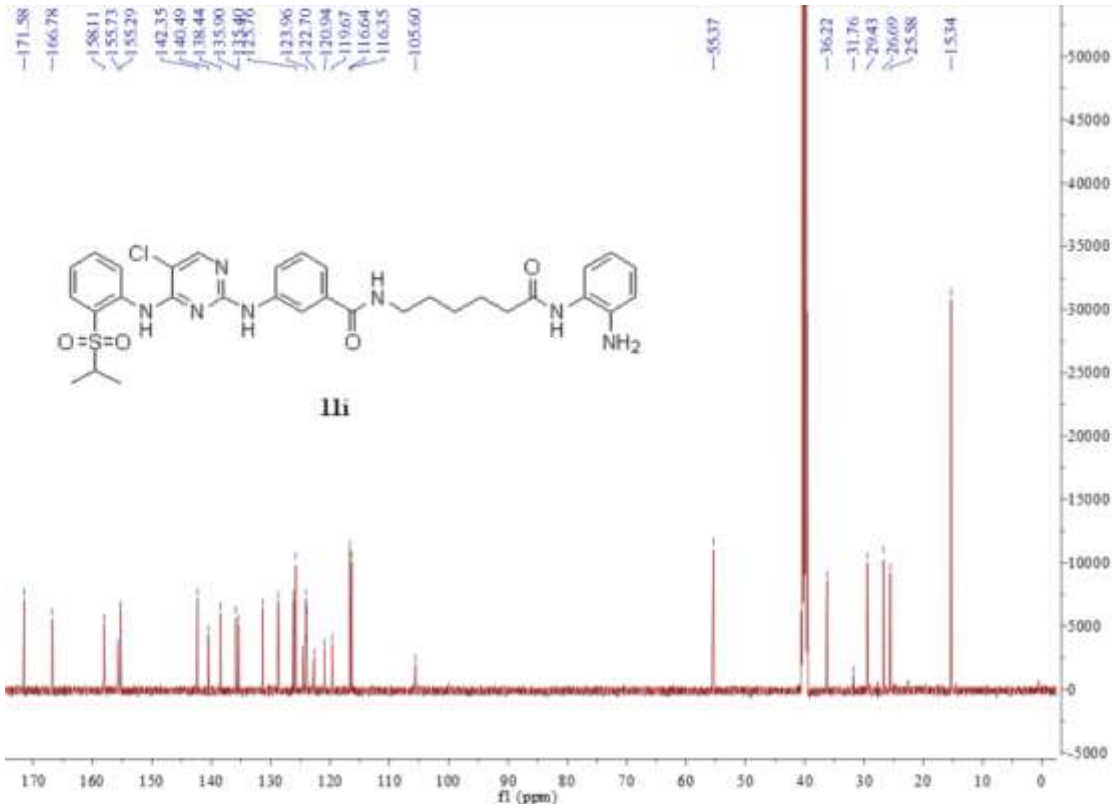

Figure 42. ^{13}C-NMR spectrum of **11i**

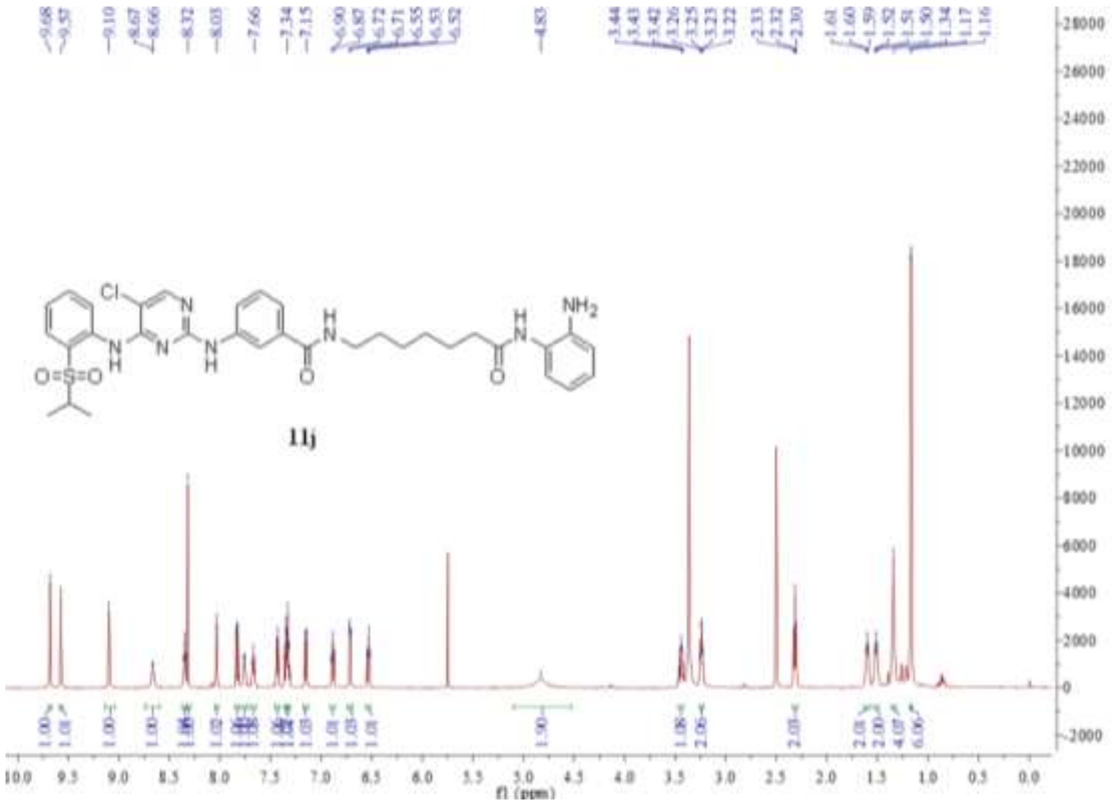

Figure 43. ^{1}H-NMR spectrum of **11j**

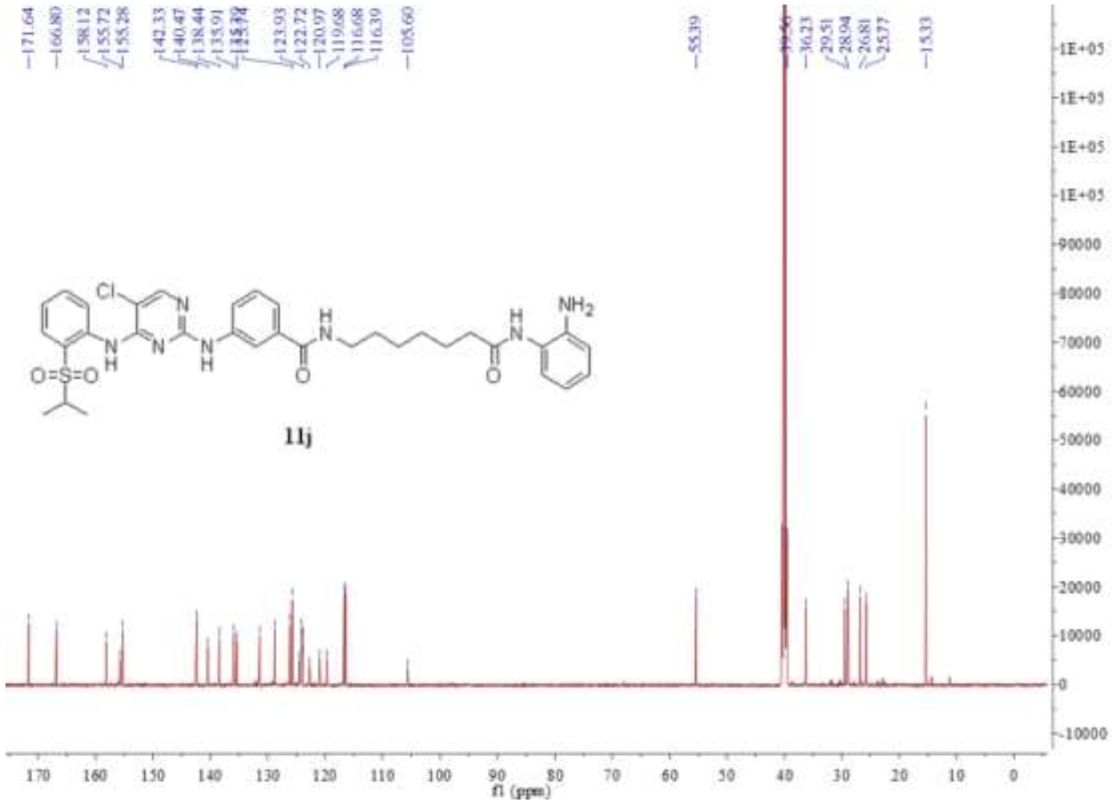

Figure 44. ^{13}C-NMR spectrum of **11j**

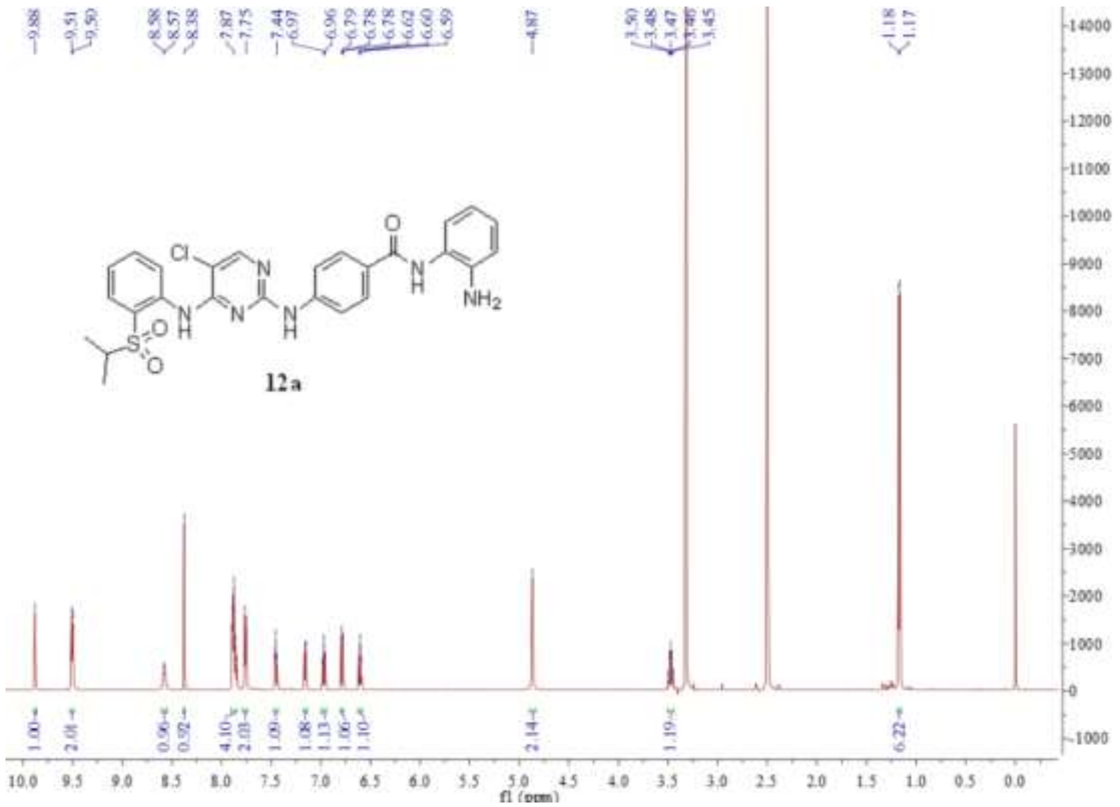

Figure 45. ^{1}H-NMR spectrum of **12a**

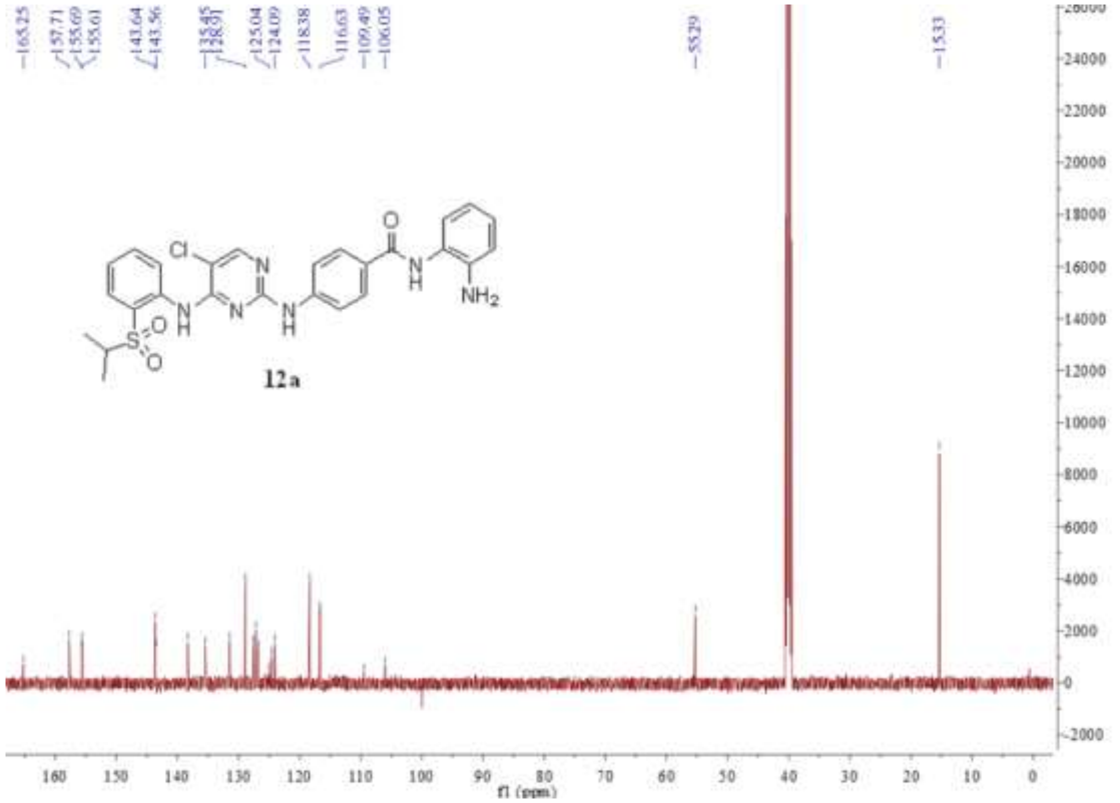

Figure 46. ^{13}C-NMR spectrum of **12a**

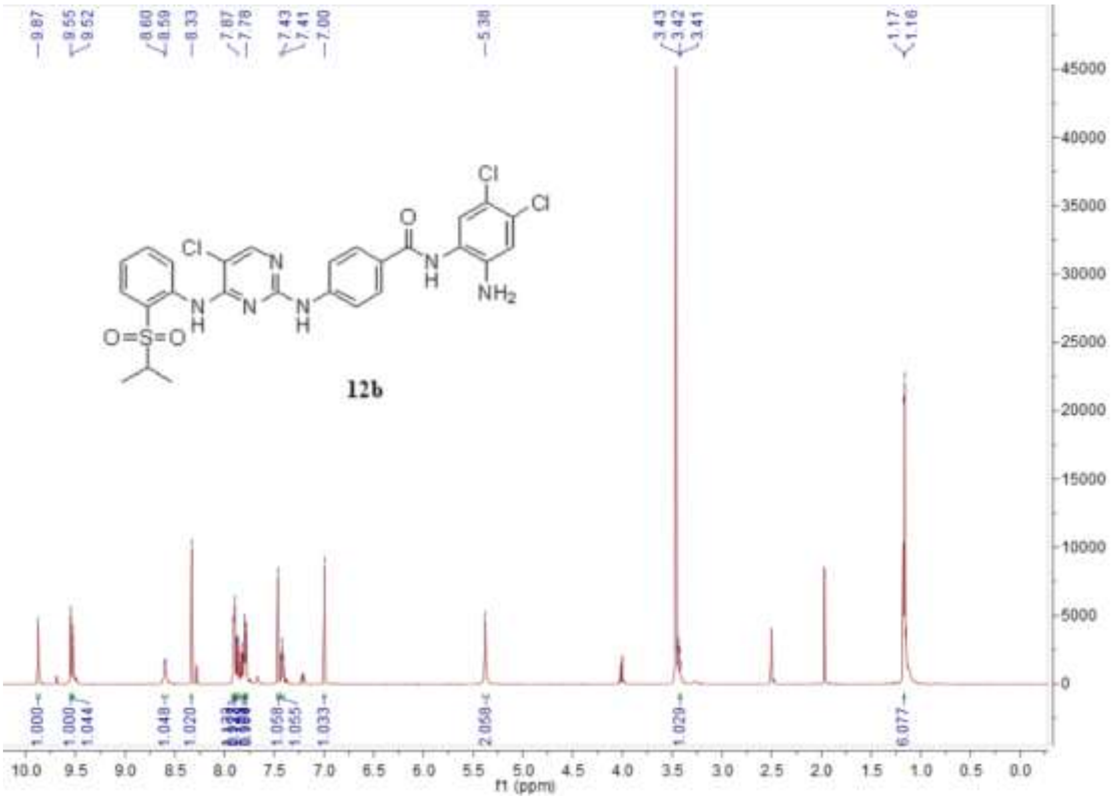

Figure 47. ^{1}H-NMR spectrum of **12b**

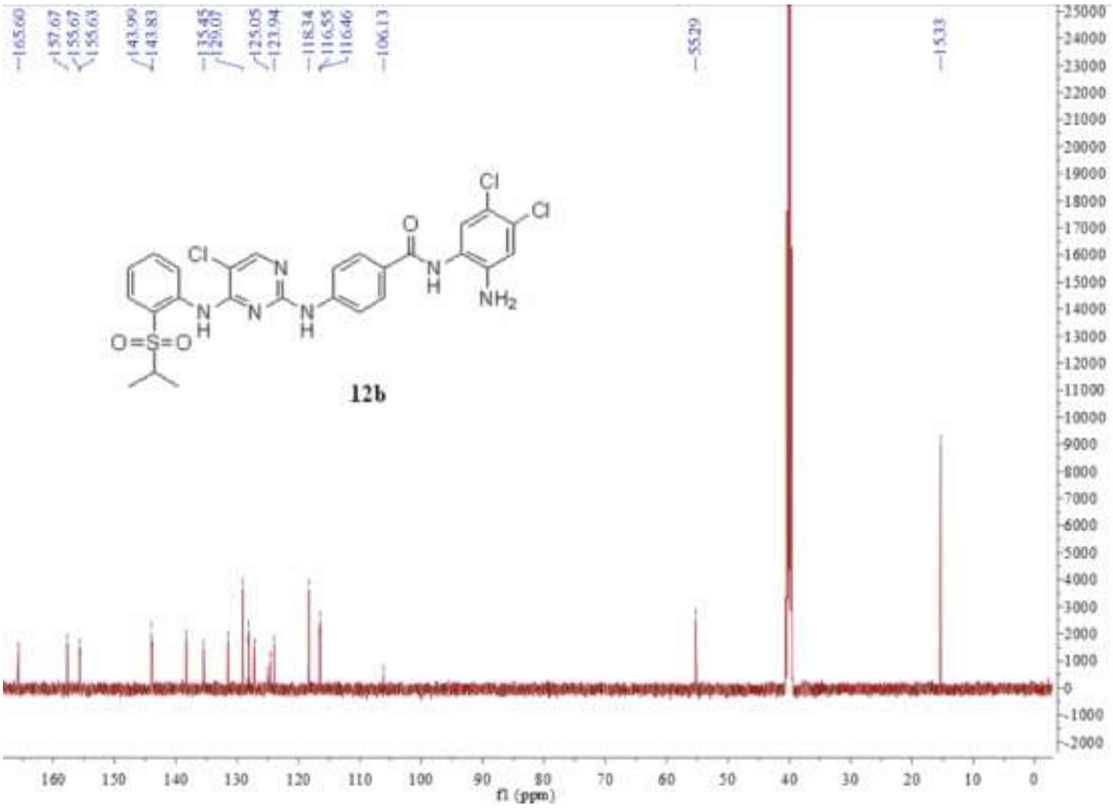

Figure 48. ^{13}C-NMR spectrum of **12b**

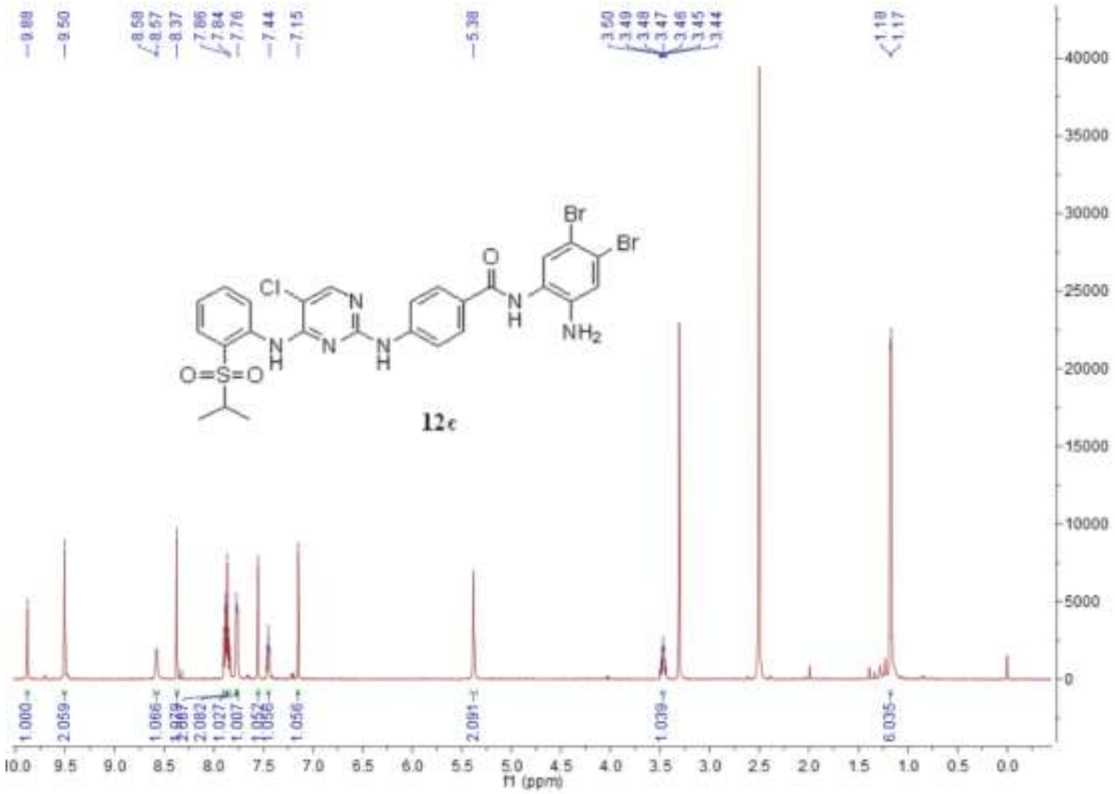

Figure 49. ^{1}H-NMR spectrum of **12c**

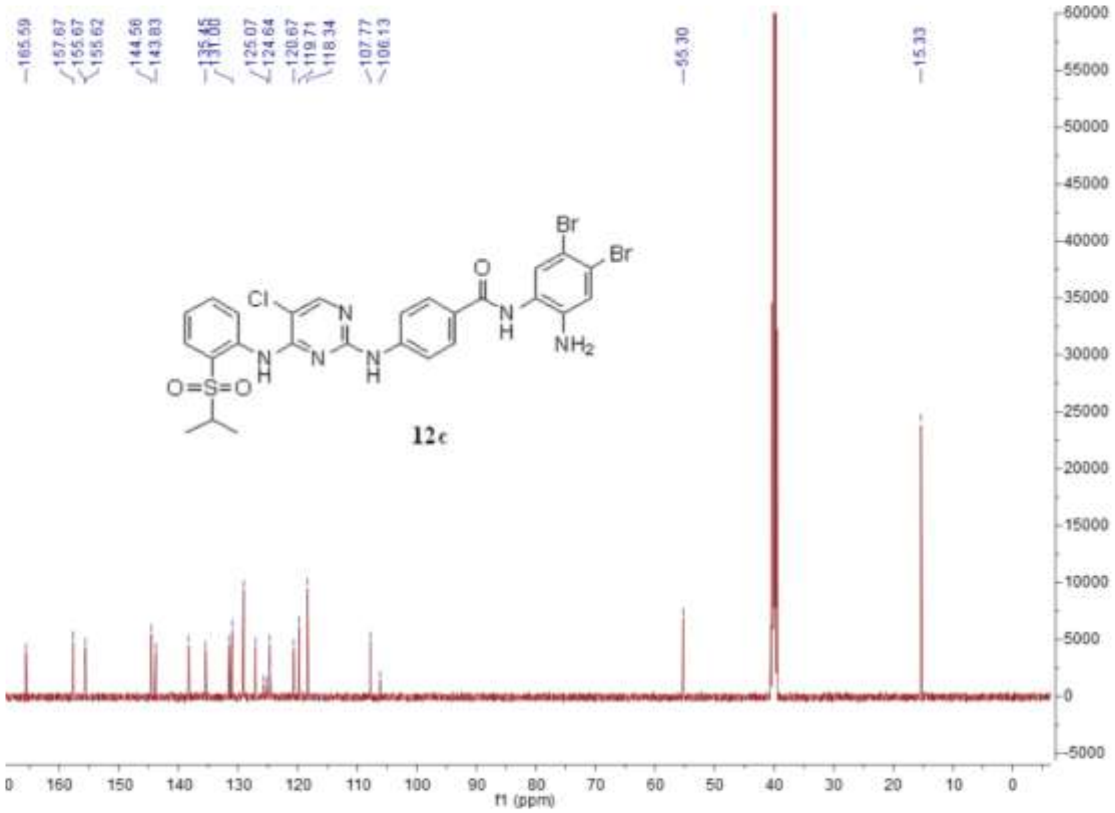

Figure 50. ^{13}C-NMR spectrum of **12c**

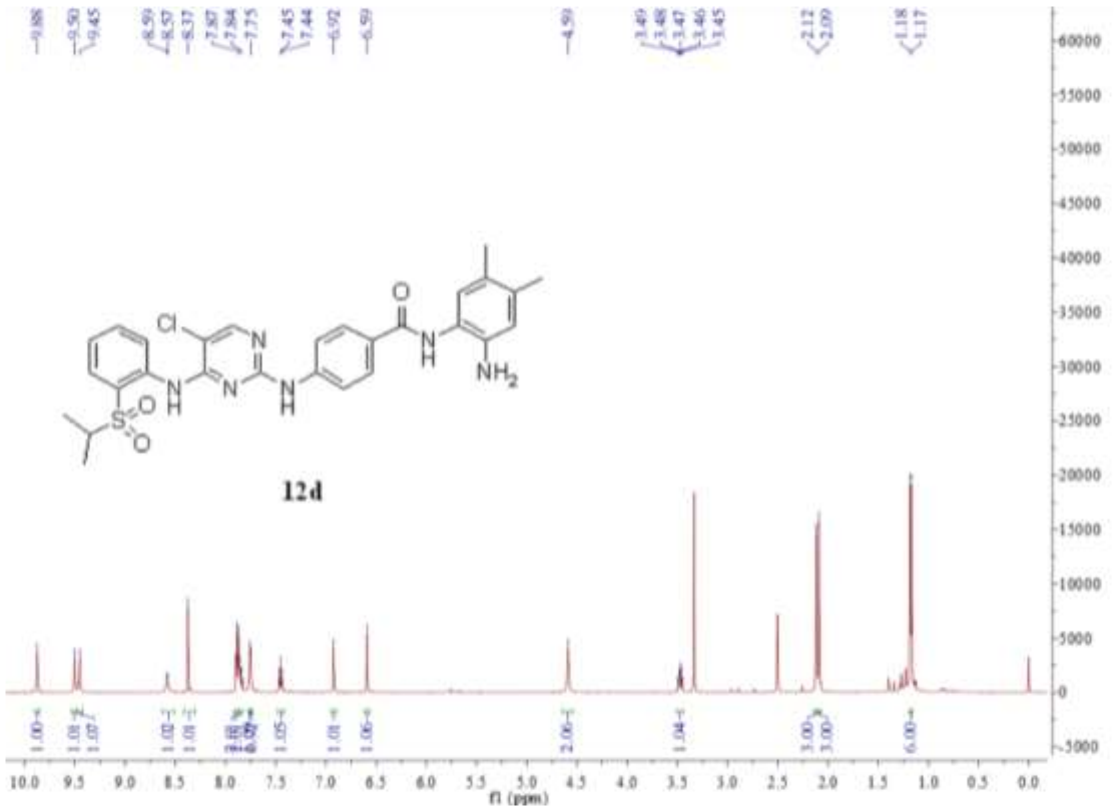

Figure 51. ^{1}H-NMR spectrum of **12d**

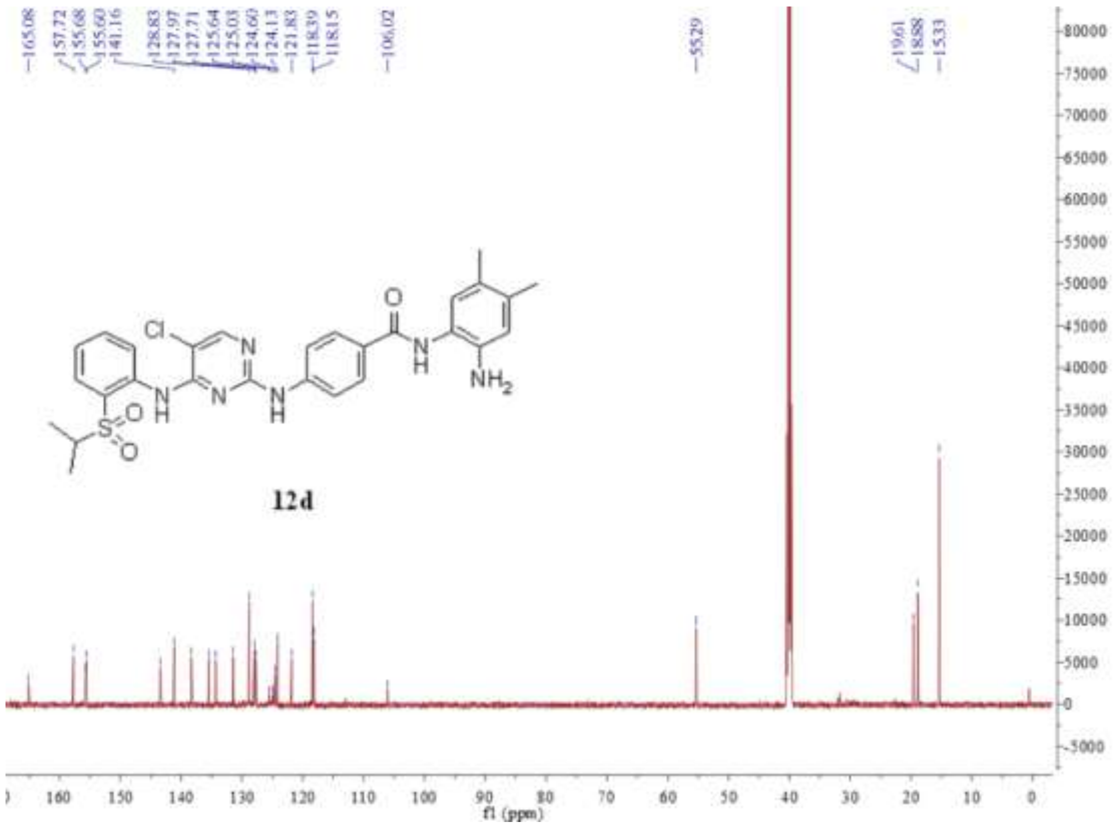

Figure 52. ^{13}C-NMR spectrum of **12d**

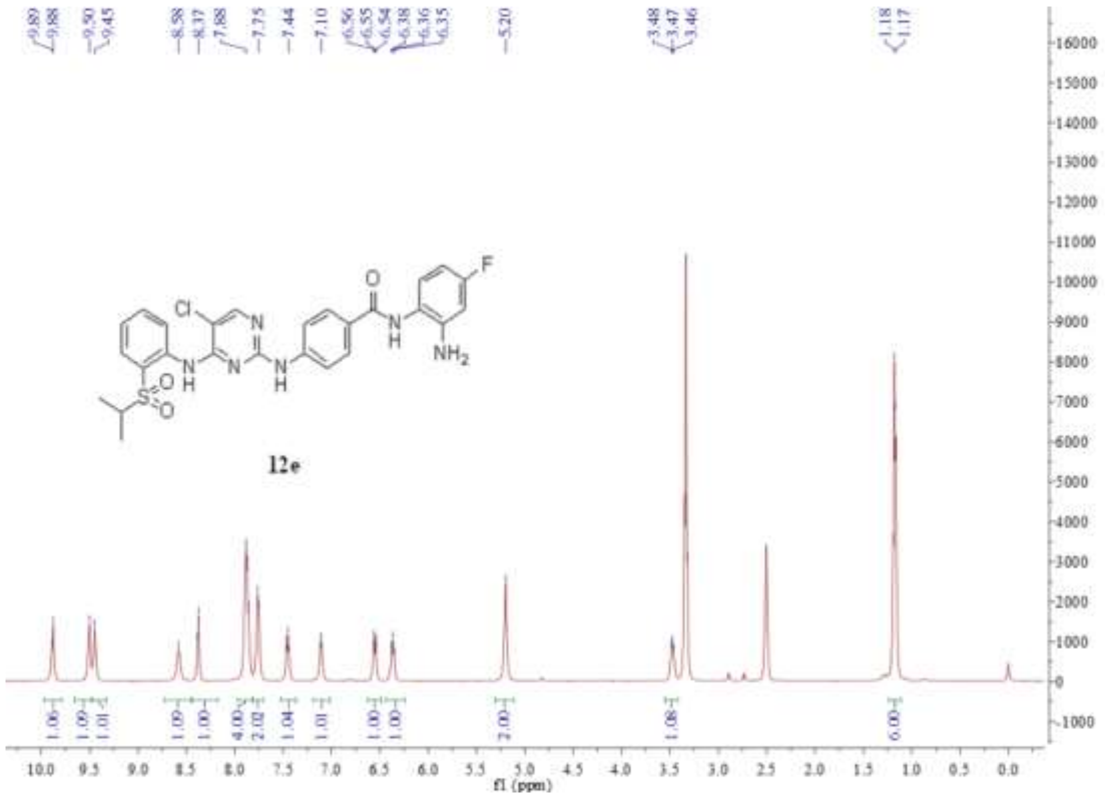

Figure 53. ^{1}H-NMR spectrum of **12e**

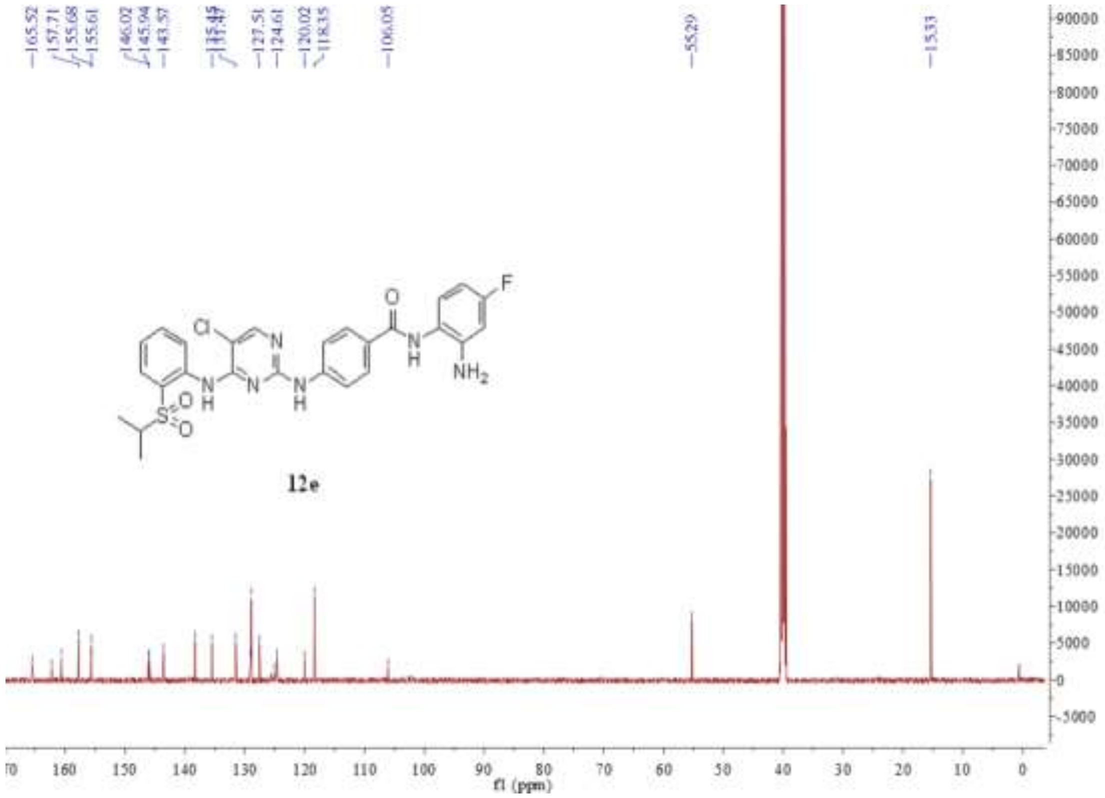

Figure 54. ^{13}C-NMR spectrum of **12e**

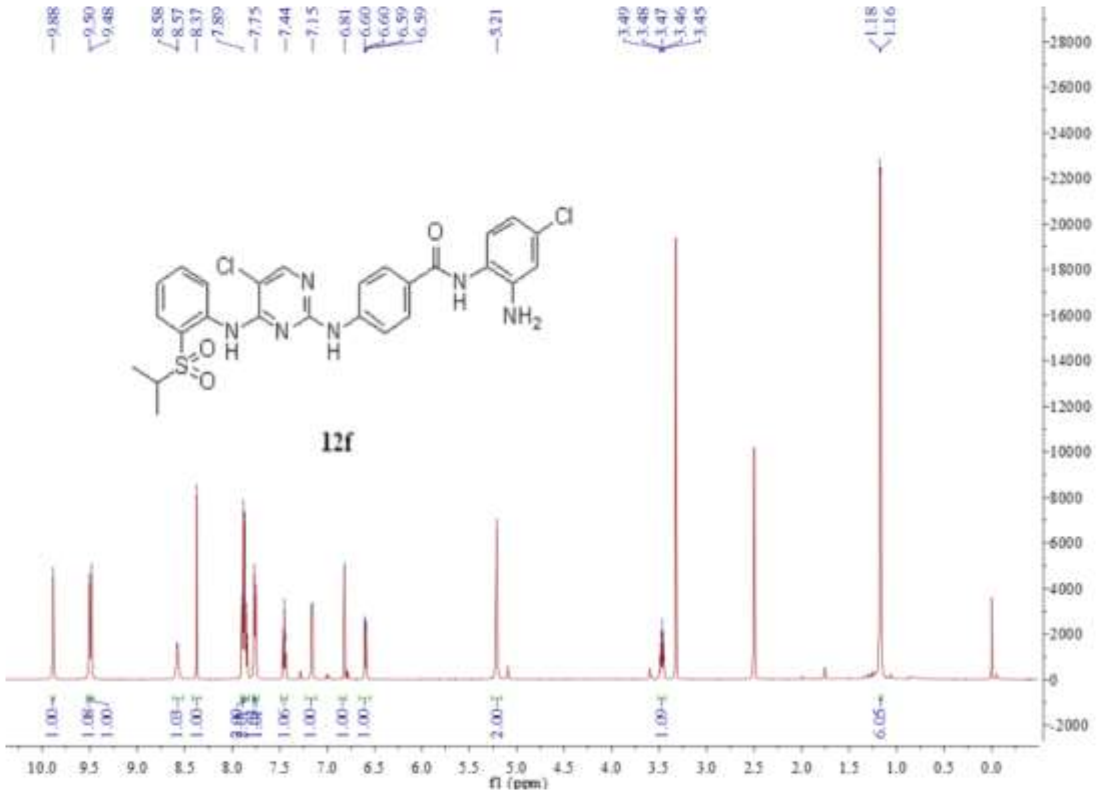

Figure 55. ^{1}H-NMR spectrum of **12f**

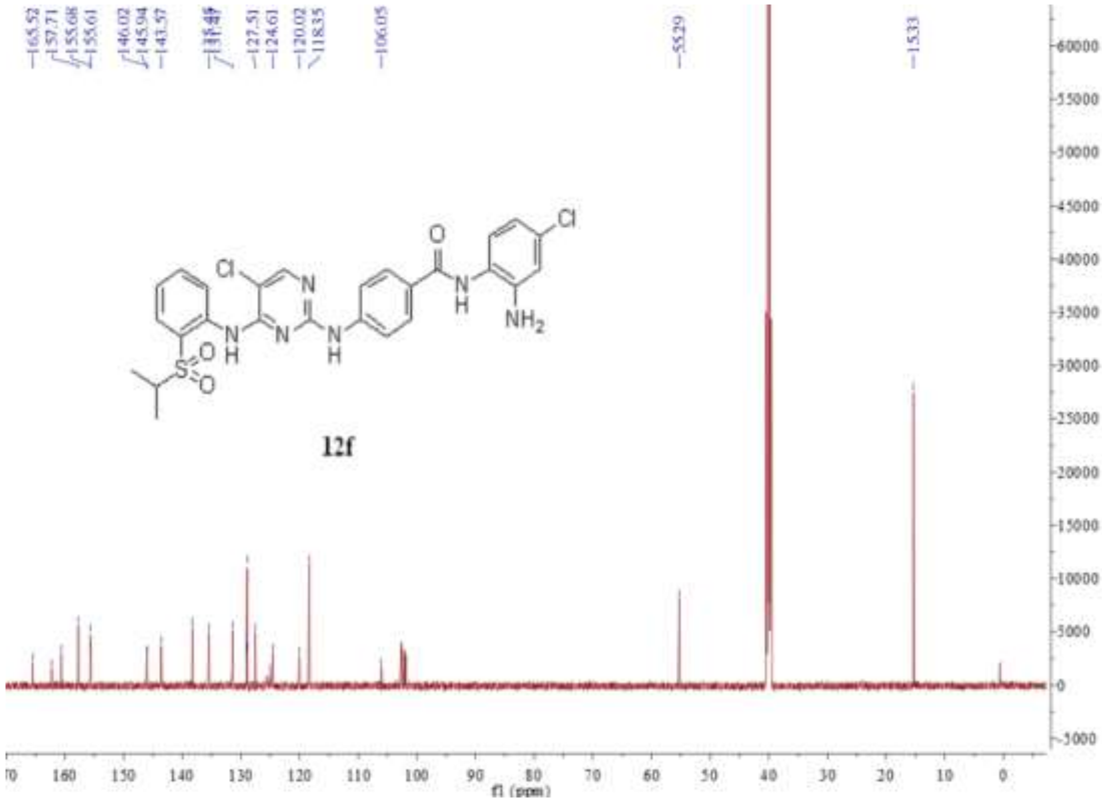

Figure 56. ^{13}C-NMR spectrum of **12f**

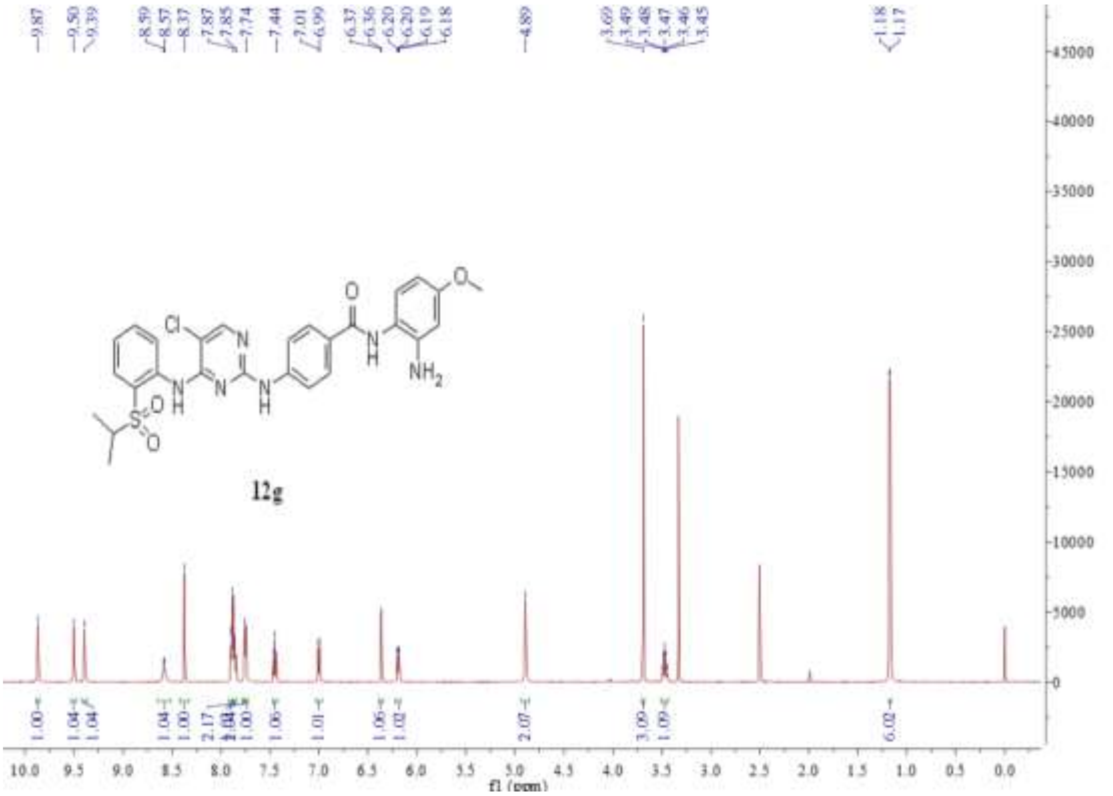

Figure 57. ^{1}H-NMR spectrum of **12g**

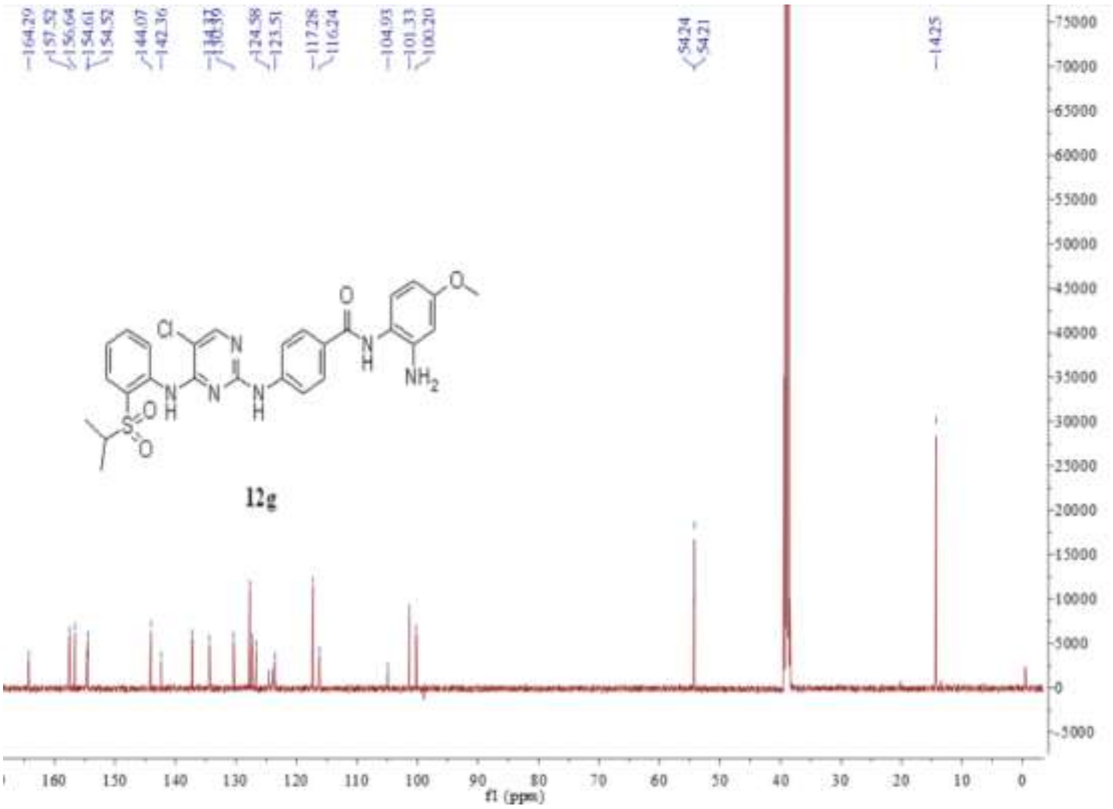

Figure 58. ^{13}C-NMR spectrum of **12g**

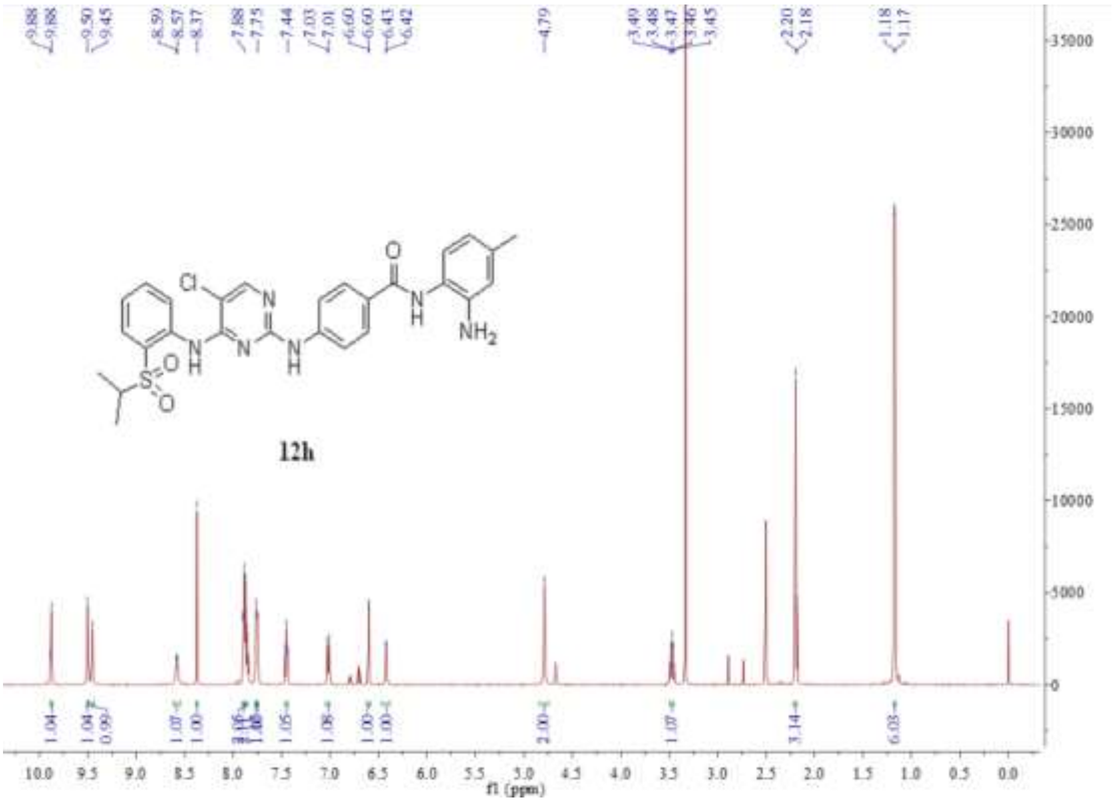

12h

Figure 59. ^{1}H-NMR spectrum of **12h**

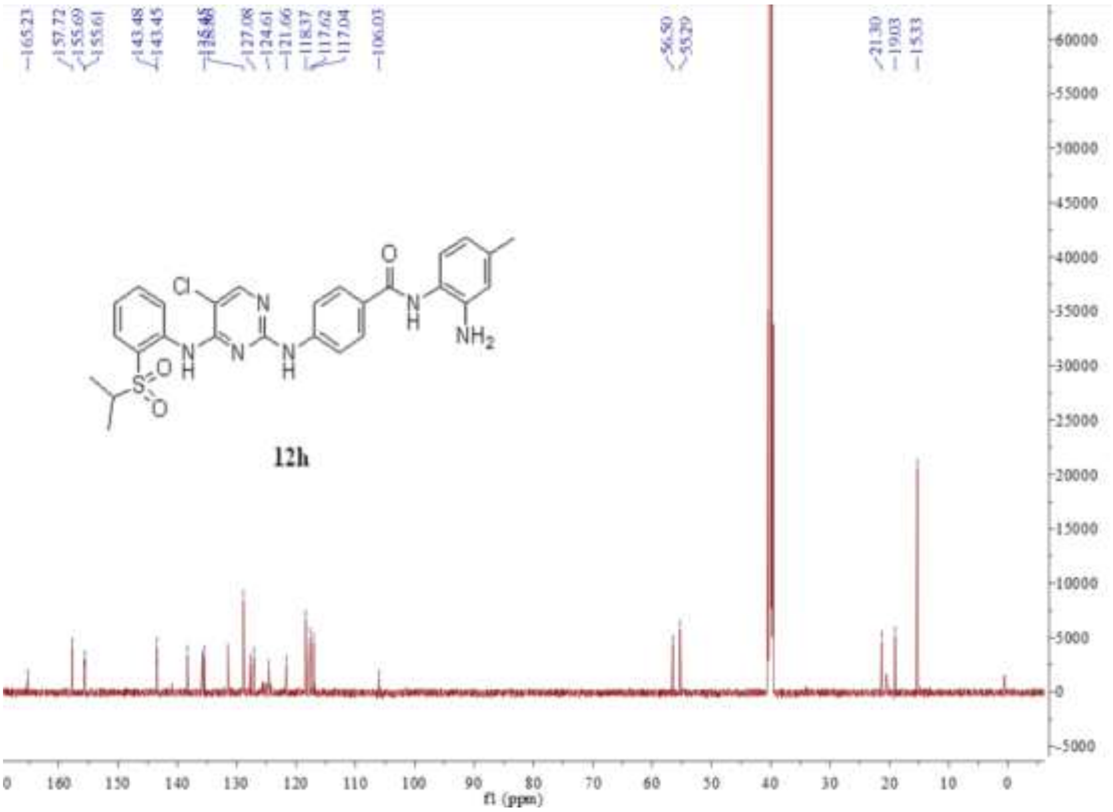

12h

Figure 60. ^{13}C-NMR spectrum of **12h**
